# The clinical and cost effectiveness of a STAndardised DIagnostic Assessment for children and adolescents with emotional difficulties: the STADIA multi‐centre randomised controlled trial

**DOI:** 10.1111/jcpp.14090

**Published:** 2025-01-07

**Authors:** Kapil Sayal, Laura Wyatt, Christopher Partlett, Colleen Ewart, Anupam Bhardwaj, Bernadka Dubicka, Tamsin Marshall, Julia Gledhill, Alexandra Lang, Kirsty Sprange, Louise Thomson, Sebastian Moody, Grace Holt, Helen Bould, Clare Upton, Matthew Keane, Edward Cox, Marilyn James, Alan Montgomery

**Affiliations:** ^1^ Unit of Mental Health & Clinical Neurosciences, School of Medicine University of Nottingham Nottingham UK; ^2^ Institute of Mental Health Nottinghamshire Healthcare NHS Foundation Trust Nottingham UK; ^3^ Nottingham Clinical Trials Unit, School of Medicine University of Nottingham Nottingham UK; ^4^ STADIA Patient and Public Involvement Co‐Lead Institute of Mental Health, University of Nottingham Nottingham UK; ^5^ Cambridgeshire and Peterborough NHS Foundation Trust Cambridge UK; ^6^ School of Medicine University of Cambridge Cambridge UK; ^7^ Hull and York Medical School University of York York UK; ^8^ Greater Manchester Mental Health Trust Manchester UK; ^9^ Pennine Care NHS Foundation Trust Ashton‐under‐Lyne UK; ^10^ Manchester Academic Health Sciences, Faculty of Biology Medicine and Health University of Manchester Manchester UK; ^11^ Berkshire Healthcare NHS Foundation Trust Bracknell UK; ^12^ Central and North West London NHS Foundation Trust London UK; ^13^ Human Factors Research Group, Faculty of Engineering University of Nottingham Nottingham UK; ^14^ Centre for Academic Mental Health, Population Health Sciences, Bristol Medical School University of Bristol Bristol UK; ^15^ MRC Integrative Epidemiology Unit, Bristol Medical School University of Bristol Bristol UK; ^16^ Gloucestershire Health and Care NHS Foundation Trust Gloucestershire UK

**Keywords:** RCT, standardised diagnostic assessment, diagnosis, emotional disorders, health economic evaluation, STADIA, Child and Adolescent Mental Health Services (CAMHS)

## Abstract

**Background:**

Standardised Diagnostic Assessment tools, such as the Development and Well‐Being Assessment (DAWBA), may aid detection and diagnosis of emotional disorders but there is limited real‐world evidence of their clinical or cost effectiveness.

**Methods:**

We conducted a multicentre, two‐arm parallel group randomised controlled trial in eight large National Health Service Trusts in England providing multidisciplinary specialist Child and Adolescent Mental Health Services (CAMHS). Participants (5–17 year‐olds with emotional difficulties referred to CAMHS) were randomly assigned (1:1), following referral receipt, to either receive the DAWBA and assessment‐as‐usual (intervention group) or assessment‐as‐usual (control group). Data were self‐reported by participants (parents and/or young person, depending on age) at baseline, 6‐ and 12‐month post‐randomisation and collected from clinical records up to 18 months post‐randomisation. The primary outcome was a clinician‐made diagnosis decision about the presence of an emotional disorder within 12 months of randomisation. Trial registration: ISRCTN15748675.

**Results:**

In total, 1,225 children and young people (58% female sex) were randomised (615 intervention; 610 control). Adherence to the intervention (full/partial completion) was 80% (494/615). At 12 months, 68 (11%) participants in the intervention group received an emotional disorder diagnosis versus 72 (12%) in the control group (adjusted risk ratio (RR) 0.94 [95% CI 0.70, 1.28]). The intervention was not cost effective. There was no evidence of any differences between groups for service‐related or participant‐reported secondary outcomes, for example, CAMHS acceptance of the index referral (intervention 277 (45%) versus control 262 (43%); RR: 1.06 [95% CI: 0.94, 1.19]) was similar between groups.

**Conclusions:**

As delivered in this pragmatic trial, we found no evidence for the effectiveness or cost effectiveness of using a Standardised Diagnostic Assessment tool in aiding the detection of emotional disorders or clinical outcomes in clinically referred children and young people. Despite regular efforts to encourage clinicians to view the DAWBA report and consider its findings as part of assessment and diagnosis, we did not collect data on usage and therefore cannot confirm the extent to which clinicians did this. As a pragmatic trial that aimed to test the effectiveness of incorporating the DAWBA into usual practice and clinical care, our study found that, in the format as delivered in this trial, there was no impact on diagnosis or clinical outcomes.

## Introduction

Emotional disorders such as depression and anxiety disorders are common in children and young people (Sadler et al., 2018). They result in significant distress and impact quality of life and functioning across a range of domains including friendships, family relationships, participation in daily activities, school attendance and attainment, and educational outcomes (Clayborne, Varin, & Colman, 2019; Costello & Maughan, 2015; Pollard et al., 2023). If unrecognised and untreated, these difficulties can persist into adulthood and are a risk factor for other mental health conditions, poor physical health, self‐harm and suicide. Although evidence‐based interventions are available, access to these requires timely and appropriate identification of difficulties. However, in many countries, service demand exceeds capacity and there are considerable barriers to receiving help. In the United Kingdom (UK), for example, these include long waiting lists and rejection of referrals by specialist Child and Adolescent Mental Health Services (CAMHS), particularly if insufficient information accompanies the referral. The evidence base to inform which referrals should be accepted is limited (Hansen, Kjaersdam Telléus, Mohr‐Jensen, Færk, & Lauritsen, 2023). If accepted for assessment, the process and purpose of assessment can vary considerably within and across services. The multi‐disciplinary nature of CAMHS in the UK means that assessments are conducted by clinicians from a range of professional backgrounds, often without formal training in making a diagnosis (Martin, Fishman, Baxter, & Ford, 2011; Michelson et al., 2011). This can lead to heterogeneous conceptualisations of presenting problems. Diagnosis is a controversial topic among mental health clinicians in the UK, reflecting concerns around the validity and role of diagnoses, stigma and labelling (Craddock et al., 2008; Martin et al., 2011; Michelson et al., 2011). In routine care, this often means that assessments do not aim to make, share or record a diagnosis, despite recommendations for using diagnostic assessments so that evidence‐based interventions are offered (Craddock et al., 2008; Simmons, Wilkinson, & Dubicka, 2015). Linked with CAMHS clinician uncertainty around the utility and value of diagnosing disorders in clinical practice, there is also clinician equipoise around the use of standardised diagnostic assessment (SDA) tools.

However, UK National Institute for Health and Care Excellence (NICE) guidelines for the care and treatment of emotional disorders are based on diagnostic classification systems; this implies that, to access evidence‐based interventions, emotional difficulties should be appropriately diagnosed. For depression, for example, NICE Quality Standards recommend that the diagnosis is confirmed and recorded (National Institute for Health and Care Excellence, 2013) but this is often not done in clinical practice (Fitzpatrick et al., 2012; Michelson et al., 2011). NICE guidelines also recommend the use of SDA tools as an adjunct to usual assessment care in detecting depression (National Institute for Health and Care Excellence, 2019). However, there is currently limited evidence about the effectiveness and cost effectiveness of using SDA tools to support routine CAMHS assessments.

Previous randomised controlled trials (RCTs) of SDA tools in CAMHS have tended to be single‐site and small‐scale. RCTs in Switzerland and England found that providing clinicians with diagnostic information from an SDA tool (the Development and Well‐Being Assessment (DAWBA); Goodman, Ford, Richards, Gatward, & Meltzer, 2000) increased agreement between DAWBA and clinical diagnoses of emotional disorders, suggesting that it could aid diagnostic decision‐making (Aebi et al., 2012; Ford et al., 2013). A feasibility trial in Denmark suggested that the DAWBA could also act as an adjunct to referral information and improve decision‐making around referral acceptance (Hansen et al., 2023). A recent study has confirmed the utility of the DAWBA for child and youth anxiety and depression (Amelio et al., 2024). In terms of the care pathway, therefore, locating SDA tools at the point that referrals are received by CAMHS could potentially optimise decisions around referral acceptance and clinician case allocation, and assessment outcomes (Last, Henley, Norman, Goodman, & Ford, 2014; Martin et al., 2011; Reeves, Charter, & Ford, 2016). This could enable a more efficient conclusion to assessments which reach a diagnostic decision, allow appropriate evidence‐based interventions to be offered, and result in better clinical outcomes. In a multicentre RCT, we aimed to investigate the effectiveness and cost effectiveness of an SDA tool for children and young people (CYP) with emotional difficulties referred to CAMHS.

## Methods

### Study design and setting

STADIA (STAndardised Diagnostic Assessment for children and adolescents with emotional difficulties) is a multicentre, two‐arm parallel group RCT (Day et al., 2022). We recruited patients from community‐based secondary care multi‐disciplinary CAMHS in eight geographically dispersed National Health Service (NHS) Trusts (organisational units serving a geographical area), covering urban and rural areas in England (Appendix S1: Table 1.1). Ethical approval was obtained from South Birmingham Research Ethics Committee (Ref. 19/WM/0133), all participants gave informed consent before taking part, and the full study protocol is available (Day et al., 2022). Changes to the protocol since the start of the trial are provided (Appendix S1: Table 1.2).

### Participants

Eligible participants were CYP, aged 5–17 years, with emotional difficulties referred to CAMHS (excluding emergency/urgent referrals requiring an expedited assessment). Participants were identified through the usual CAMHS referral pathways via Single/Central Point of Access triage teams which have a gatekeeping function. Referrals were screened by STADIA researchers (Appendix S1: Figure 1.1) and eligibility checked based on information provided within the referral letter. Potentially eligible participants were invited to participate in the trial and written information provided. Eligibility and verbal consent were confirmed during a telephone call and participants were provided with an e‐link to the online electronic informed consent/assent form to give written informed consent/assent. Participant flow is described in the Figure. CYP sex data (female/male) were collected from CAMHS clinical records by site researchers and gender (female/male/other) self‐reported by participants.

For CYP aged 5–15 years, parents/carers with parental responsibility provided informed consent and data (primary participant), with an option for 11–15 year‐olds (secondary participant) to provide assent and data. Young people aged 16–17 (primary participant) provided informed consent and data, and with their permission their parent/carer (secondary participant) could also participate (Appendix S1: Table 1.3). As all study procedures were completed electronically, participants needed access to the internet and email.

### Randomisation and masking

Participants were randomised in a 1:1 ratio to either the Development and Well‐Being Assessment (DAWBA) plus assessment‐as‐usual (intervention), or assessment‐as‐usual only (control). Allocation was assigned using a minimisation algorithm balanced by recruiting site, CYP age (5–10, 11–15 and 16–17 years) and sex, minimising imbalance with 80% probability. The allocation algorithm was created and concealed using a secure, automated web system operated by Nottingham Clinical Trials Unit.

Participants were randomised after submitting their baseline data and were informed of their allocation. An email also confirmed allocation, and instructions for DAWBA completion were included for the intervention group. Data were collected from clinical records (source data), using Case Report Forms (CRFs), by site researchers at 12‐ and 18‐month post‐randomisation.

It was not possible to mask participants, site researchers, clinicians and some trial staff to treatment allocation, nor was it possible to mask researchers collecting outcome data from records. However, any possible diagnoses identified from the CAMHS records were recorded verbatim on the CRF and subjected to adjudication by the outcome adjudication committee (members of the Trial Management Group). The adjudication committee and trial statisticians were masked to treatment allocation and participant ID. The outcome definition and adjudication procedures are described in Appendix S1: Table 1.4.

### Procedures

Participant‐reported data were collected through online questionnaires (parent/carer and CYP self‐report aged 11+), at baseline and 6‐ and 12‐month post‐randomisation. As per consent requirements, questionnaires were completed by the primary participant (parent/carer for CYP 5–15 years, and young people aged 16–17), with the option for the secondary participant to also contribute. Baseline measures collected included: socio‐demographic data, Mood and Feelings Questionnaire (MFQ), Revised Children's Anxiety and Depression Scale (RCADS), Strengths and Difficulties Questionnaire (SDQ) (a comprehensive list of assessments is included in Appendix S1: Table 1.5).

#### Intervention group

The trial intervention was the DAWBA (Goodman et al., 2000), completed by participants (after completion of the baseline questionnaire) via the secure online platform maintained by the DAWBA developer (Youth in Mind, 2012). We aimed for the DAWBA to be completed within 10 working days of referral receipt (Day et al., 2022). Participants were asked to complete all modules presented to them. Modules related to emotional and other specific comorbid disorders: separation anxiety disorder, specific phobia, social phobia, panic disorder and agoraphobia, generalised anxiety disorder, post‐traumatic stress disorder (PTSD), obsessive compulsive disorder, depression, oppositional defiant disorder and conduct disorder; no free‐text responses were collected. In conjunction with study Patient and Public Involvement panels, a trial‐specific DAWBA report template (Appendix S1: Figure 1.3) was developed. A DAWBA report was prepared for each participant, with algorithm‐derived diagnostic predictions (Goodman, Heiervang, Collishaw, & Goodman, 2011) used to highlight the likelihood of a CYP meeting ICD‐10 criteria (World Health Organisation, 1992) for the disorders assessed (close to average, slightly raised, high, very high). By basing the report entirely on the algorithm‐derived diagnostic predictions, the intervention was deliberately pragmatic in nature – so that, if found to be effective and cost effective, it could potentially be readily implemented into routine practice in services.

The DAWBA report was uploaded to the CAMHS clinical record (electronic) for clinicians to access, as an adjunct to usual clinical care (see below). We ensured that site researchers worked closely with and directly informed clinicians in the Single/Central Point of Access triage teams when the DAWBA report was available for their review so that they could proceed with their decision‐making around referral acceptance. As indicated in Appendix S1: Table 2.9, the median time from randomisation to referral decision was 12 calendar days (i.e. 8–10 working days).

The DAWBA report was also sent out to study participants and, as part of the covering letter, they were encouraged to share the report at any appointments that they might have with the CAMHS team or with any other healthcare professionals. Furthermore, as the DAWBA report was uploaded to the CAMHS clinical records, periodic reminders about its presence were added into the clinical notes (i.e. reminders that a DAWBA report is available to view in the clinical records). There were also regular (6 monthly) presentations from the site Principal Investigators to their local CAMHS teams to actively engage clinicians, maintain awareness of the trial and remind clinicians to check for DAWBA reports in the clinical records (these interactive meetings also included visual examples of what DAWBA reports look like, with information shared about how to understand and interpret a report).

#### Control group

Participants randomised to the control group received usual clinical care (i.e. referral review and assessment‐as‐usual).

### Outcomes

The primary outcome was a clinician‐made diagnosis decision about the presence of an emotional disorder within 12 months of randomisation. Eligible diagnoses were predefined (see Appendix S1: Table 1.6) using precise diagnostic terminology and reflected emotional or internalising disorders as outlined in ICD‐10/DSM‐IV. Classification of a diagnosis required the suffix ‘disorder’ for certain types of difficulties, for example ‘generalised anxiety disorder’, ‘obsessive compulsive disorder’. Where similar terminology was used, for example ‘anxiety’ without ‘disorder’, ‘symptoms of…’, ‘…‐type symptoms/behaviour,’ etc., these were referred for adjudication and were classified as not constituting a clinical diagnosis due to uncertainty around the presence of an emotional disorder. The diagnosis must have been documented in the CAMHS clinical records within 12 months of randomisation by a mental health services clinician in an NHS‐delivered or NHS‐funded service, that is, diagnosis data were captured from routine clinical records.

Secondary outcomes collected were service‐related or participant‐reported (full list of measures in Appendix S1: Table 1.7). During the trial, additional secondary outcomes were included to extend follow‐up to 18‐month post‐randomisation due to the impact of covid‐19 pandemic‐related delays to service access and receipt (Appendix S1: Table 1.2). Following the pandemic, the Children's Revised Impact of Event Scale (CRIES‐8) was added to enquire about PTSD symptoms.

Service‐related secondary outcomes over the 12‐ and 18‐month period from randomisation were: referral acceptance (index and subsequent); discharge from CAMHS; confirmed diagnosis decision; time from randomisation to diagnosis of an emotional disorder; decision to offer and start treatment/intervention: (a) for a diagnosed emotional disorder; (b) whether or not there is a documented diagnosis; and time to offer or start any treatment/intervention (in addition to recording diagnoses and all treatments/interventions given).

Participant‐reported secondary outcomes were participant–self‐reported diagnoses, depression symptoms (MFQ), anxiety symptoms (RCADS), oppositional defiant/conduct disorder symptoms and functional impairment (SDQ). For participants in the intervention group, the SDQ was part of the DAWBA. Self‐harm thoughts and behaviours were self‐reported by CYP. Parent‐reported depression and anxiety symptoms were collected (Patient Health Questionnaire, PHQ‐9 and Generalised Anxiety Disorder Assessment; GAD‐7, respectively). Health‐related quality of life measures collected for the economic evaluation were the EQ‐5D‐Y and the Child Health Utility 9D (CHU9D) for CYP and the EQ‐5D‐5L for parents/carers. Further details and references for the outcome measures are found in the Appendix S1: Table 1.7.

### Patient and public involvement

Patient and public involvement (PPI) has been strongly embedded throughout the lifecycle of this trial (Day et al., 2022), playing a vital and valuable role in formative and summative work, across a range of project workstreams. Our PPI parent/carer co‐investigator (CE) led a programme of workshops and ongoing engagement with our parent/carer and young person's PPI groups, retaining all 14 members over the 5‐year project duration. Our PPI lead and groups worked closely with the research team to develop the intervention, inform the choice of measures including the content of the resource use questionnaire, advise on recruitment and retention approaches, and interpret and advise on emerging findings.

### Sample size and data analysis

With 544 participants per group for analysis, a between‐group absolute difference of 10 percentage points in the primary outcome is detectable with 90% power and 5% two‐sided alpha. Allowing for up to 10% non‐collection of the primary outcome, we aimed to randomise 1,210 participants. Unpublished data obtained from trial sites suggested 45% of control participants would receive a confirmed diagnosis within 12 months; our target sample size enabled detection of smaller absolute effects if the observed diagnosis rate was lower than 45%.

Baseline characteristics of CYP and parents/carers were summarised using mean, standard deviation, median, lower and upper quartiles, minimum, maximum and number of observations for continuous data, and frequency counts and percentages for categorical data. The primary approach to between‐group comparative analyses was to include all participants with observed outcome data according to randomised allocation. The primary analysis employed a generalised linear mixed model to compare the proportions in each group with a clinician‐made diagnosis decision within 12 months of randomisation, adjusted for minimisation variables.

Secondary outcomes were analysed using appropriate mixed effect regression models dependent on data type and adjusted for minimisation factors and baseline value of the outcome where measured. Outcomes measured at multiple time points were analysed using a mixed model with a treatment by time interaction to obtain estimates of treatment effect at each follow‐up time.

Appropriate interaction terms were included in the primary regression analyses to conduct subgroup analyses according to CYP sex and age. Statistical analysis was conducted using Stata v18.0. The Statistical Analysis Plan was finalised and approved prior to database lock. A minor change was made following database lock and can be found in the Appendix S1. Data were periodically presented by arms to the Data Monitoring Committee.

### Health economic analysis

A detailed description of the health economics methods and results are contained in the Appendices (Section 3). The Health Economics Analysis Plan (HEAP) was finalised and approved prior to database lock (see Appendix S1). In line with NICE guidance, the primary viewpoint for the cost analysis was from an NHS and personal social service (PSS) perspective (National Institute for Health and Care Excellence, 2023). Secondary analyses considered costs from a broader societal perspective that included productivity losses and out‐of‐pocket expenses. The following resource use and costs were collected at baseline, 6 and 12 months using a tool designed in collaboration with our PPI groups: health service resource use (medication use and primary, secondary and community care utilisation), social care (e.g. social worker, home help), out‐of‐pocket expenses (e.g. travel and over‐the‐counter medications) and employment (productivity – time lost from paid employment). The DAWBA cost (£10) was applied at the participant‐level across the intervention group (Youth in Mind, 2012). Health‐related quality of life (HRQoL) preference scores for the EQ‐5D‐5L, EQ‐5D‐Y and CHU9D were derived using relevant population tariffs, and quality‐adjusted life years (QALY) estimated using an area under the curve approach and linear interpolation between EQ‐5D‐Y assessments (see Appendix S1: Section 3).

The economic evaluation took an incremental approach between the two groups using an intention‐to‐treat population and a 12‐month time horizon. Missing data were populated using Multiple Imputation by Chained Equations, which builds the inherent uncertainty associated with missing data by specifying a separate conditional distribution for each imputed variable (Royston & White, 2011; White, Royston, & Wood, 2011). Costs and outcomes were collected directly from 16 to 17 year‐old CYP; for 5–15 year‐olds, the parent/carer proxy response was used. Exploratory analyses investigated cost effectiveness with NHS and PSS costs, broader societal costs and EQ‐5D‐Y preference scores and associated QALYs on a complete case basis (i.e. costs and outcomes from participants who completed all necessary follow‐up information (see Appendix S1: Table 3.6).

Between‐group differences were estimated through seemingly unrelated regressions (Davidson & MacKinnon, 1993; see Appendix S1: Section 3). Uncertainty in between‐group cost and QALY differences were presented using scatter plots (Drummond, Schulpher, Claxton, Stoddart, & Torrance, 2015) and cost effectiveness acceptability curves showing the probability that the intervention is cost effective compared with control up to a £30,000 per QALY willingness to pay threshold (Fenwick, Claxton, & Sculpher, 2001; Appendix S1: Figures 3.1–3.4). Costs and QALYs were not discounted as they accrue within a 12‐month time‐horizon.

### Registration and role of the funding source

The study was prospectively registered as ISRCTN15748675. The funder had no role in study design, data collection, analysis or interpretation, or writing of the report.

## Results

### Participant flow and baseline data

**Figure 1:**
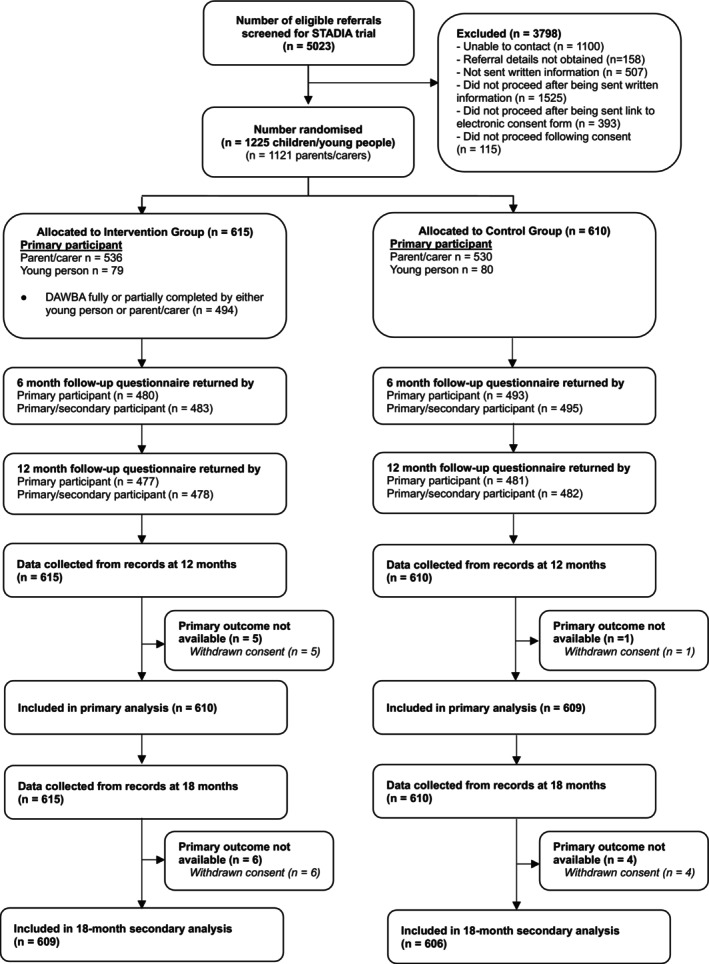
CONSORT flow diagram

We recruited 1,225 participants between August 27, 2019 and October 17, 2021 from eight NHS Trusts in England; 615 to the intervention group and 610 to the control group (Figure 1) and follow up was completed on April 17, 2023. The primary participant was the parent/carer in 87% of cases. Baseline characteristics were well balanced across randomised groups, for both CYP and parents/carers (Table 1).

**Table 1:** Baseline characteristics (minimisation factors italicized)

Characteristics	Intervention group	Control group
	(*n* = 615)	(*n* = 610)
Child/Young person baseline characteristics		
*Age at randomisation* (years)		
Mean [*SD*]	11.9 [3.1]	12 [3.1]
Median [25th, 75th centile]	12 [10, 14]	13 [9, 15]
Min, max	5, 17	5, 17
5–10	212 (34%)	210 (34%)
11–15	324 (53%)	320 (52%)
16–17	79 (13%)	80 (13%)
*Sex* ^a^		
Male	257 (42%)	254 (42%)
Female	358 (58%)	356 (58%)
Gender^b^		
Male	256 (42%)	252 (42%)
Female	344 (57%)	339 (57%)
Other	6 (1%)	7 (1%)
Missing	9	12
Ethnicity		
White	524 (86%)	516 (86%)
Indian	10 (2%)	12 (2%)
Pakistani	9 (1%)	6 (1%)
Bangladeshi	1 (<1%)	0 (0%)
Black Caribbean	6 (1%)	6 (1%)
Black African	0 (0%)	2 (<1%)
Black (other)	1 (<1%)	0 (0%)
Chinese	2 (<1%)	0 (0%)
Other Asian (Non‐Chinese)	6 (1%)	6 (1%)
Dual/mixed heritage	41 (7%)	45 (8%)
Other	7 (1%)	5 (1%)
Missing	8	12
Index of Multiple Deprivation quintile (child's primary residence)		
1st quintile (most deprived)	112 (18%)	102 (17%)
2nd quintile	116 (19%)	122 (20%)
3rd quintile	143 (23%)	132 (22%)
4th quintile	105 (17%)	104 (17%)
5th quintile (least deprived)	138 (22%)	149 (24%)
Missing	1	1
Prior CAMHS referral^c^		
No	416 (68%)	429 (70%)
Yes	199 (32%)	181 (30%)
Previous or existing diagnosis of an emotional disorder in CAMHS records		
No	577 (94%)	572 (94%)
Yes	38 (6%)	38 (6%)
Parent/Carer baseline characteristics		
Relationship to child		
Mother	510 (91%)	521 (93%)
Father	41 (7%)	27 (5%)
Grandparent	6 (1%)	4 (1%)
Other	2 (<1%)	6 (1%)
Missing	1	3
Age at randomisation (years)		
<20	1 (<1%)	2 (<1%)
20–29	22 (4%)	31 (6%)
30–39	208 (37%)	176 (31%)
40–49	249 (44%)	254 (45%)
50–59	76 (14%)	88 (16%)
60 or over	4 (1%)	8 (1%)
Missing	0	2
Gender		
Male	44 (8%)	31 (6%)
Female	516 (92%)	528 (94%)
Missing	0	2
Ethnicity		
White	514 (92%)	508 (91%)
Indian	8 (1%)	13 (2%)
Pakistani	8 (1%)	6 (1%)
Black Caribbean	6 (1%)	6 (1%)
Black African	0 (0%)	1 (<1%)
Black (other)	0 (0%)	2 (<1%)
Chinese	2 (<1%)	0 (0%)
Other Asian (Non‐Chinese)	4 (1%)	6 (1%)
Dual/mixed heritage	11 (2%)	10 (2%)
Other	6 (1%)	6 (1%)
Missing	1	3

All data are *N* (%) unless otherwise indicated. CAMHS, Child and Adolescent Mental Health Services.

^a^ Collected from CAMHS records.

^b^ Self‐reported by participant.

^c^ Predominantly self‐reported by participant, however if missing it was augmented using data from CAMHS records.

Adherence to the intervention was high with 494 (80%) participants either fully or partially completing the DAWBA, of whom 332 (67%) scored ‘very high’ in at least one emotional disorder domain, most commonly depression and generalised anxiety (Table 2).

**Table 2:** DAWBA summary

	*n*	Close to average	Slightly raised	High	Very high
Separation anxiety	461	223 (48%)	178 (39%)	1 (<1%)	59 (13%)
Specific phobias	479	323 (67%)	1 (<1%)	52 (11%)	103 (22%)
Social phobia	478	187 (39%)	90 (19%)	87 (18%)	114 (24%)
Panic attacks	478	298 (62%)	84 (18%)	65 (14%)	31 (6%)
Agoraphobia	478	277 (58%)	97 (20%)	81 (17%)	23 (5%)
Generalised anxiety	479	48 (10%)	141 (29%)	116 (24%)	174 (36%)
Obsessive compulsive disorder	480	368 (77%)	41 (9%)	46 (10%)	25 (5%)
Post‐traumatic stress disorder	477	410 (86%)	20 (4%)	34 (7%)	13 (3%)
Depression	484	186 (38%)	10 (2%)	93 (19%)	195 (40%)
At least one emotional disorder domain scoring ‘Very High’^a^	494				332 (67%)
Oppositional defiant disorder	428	74 (17%)	101 (24%)	90 (21%)	163 (38%)
Conduct disorder	474	235 (50%)	132 (28%)	40 (8%)	67 (14%)

*Note*: All data are *N* (%) unless otherwise indicated. NB: DAWBA questions reflect ICD‐10 and DSM‐IV; the algorithm is based on ICD‐10 diagnostic criteria.

^a^ Oppositional Defiant Disorder and Conduct Disorder not included and denominator is the number of participants who completed/partially completed a DAWBA.

### Outcomes

There was a high retention for the primary outcome (>99%); six participants (five intervention, one control) withdrew consent to access CAMHS records within 12 months and prior to any emotional disorder diagnosis, therefore we included 610 in the intervention group and 609 in the control group in the primary analysis.

There was no evidence of a difference between groups for the primary outcome; 68 (11%) participants in the intervention group received an emotional disorder diagnosis within 12 months versus 72 (12%) in the control group (Table 3). There was no evidence that between‐group effects differed according to CYP's age or sex (Table 4) or of any differences between groups for any of the secondary outcomes from CAMHS records (Table 3).

**Table 3:** Outcomes from CAMHS records

Outcome	Time point	Intervention group (*n* = 615)	Control group (*n* = 610)	Adjusted^a^ treatment effect (95% CI)
Primary outcome				
Clinician‐made diagnosis decision about the presence of an emotional disorder	12 months	68/610 (11%)	72/609 (12%)	RR: 0.94 (0.70, 1.28); *p*‐value = .71 RD: −0.63 (−3.99, 2.72)
Secondary outcomes				
Clinician‐made diagnosis decision about the presence of an emotional disorder	18 months	84/609 (14%)	91/606 (15%)	RR: 0.92 (0.71, 1.19) RD: −1.25 (−5.06, 2.57)
Diagnosis of emotional disorder (categorical)	12 months			
(1) Emotional disorder diagnosis documented in clinical records		68/610 (11%)	72/609 (12%)	
(2) Having a clearly documented absence of an emotional disorder		26/610 (4%)	17/609 (3%)	
(3) Uncertainty in presence of an emotional disorder		170/610 (28%)	135/609 (22%)	
(4) No diagnostic information		346/610 (57%)	385/609 (63%)	
Diagnosis of emotional disorder (categorical)	18 months			
(1) Emotional disorder diagnosis documented in clinical records		84/609 (14%)	91/606 (15%)	
(2) Having a clearly documented absence of an emotional disorder		32/609 (5%)	22/606 (4%)	
(3) Uncertainty in presence of an emotional disorder		179/609 (29%)	144/606 (24%)	
(4) No diagnostic information		314/609 (52%)	349/606 (58%)	
Confirmed diagnosis decision (diagnosis of an emotional disorder or confirmed absence of an emotional disorder)	12 months	94/610 (15%)	89/609 (15%)	RR: 1.07 (0.82, 1.39) RD: 0.92 (−2.77, 4.61)
	18 months	116/609 (19%)	113/606 (19%)	RR: 1.03 (0.82, 1.29) RD: 0.55 (−3.55, 4.65)
Time from randomisation to diagnosis of emotional disorder (days)	12 months			HR: 0.92 (0.66, 1.29)
Mean [*SD*]		108.1 [109.7]	99.8 [104.3]	
Median [25th, 75th centile]		54.5 [26, 168.5]	47.5 [27, 143.5]	
Time from randomisation to diagnosis of emotional disorder (days)	18 months			HR: 0. 91 (0.67, 1.22)
Mean [*SD*]		139.5 [143.1]	140.1 [151.9]	
Median [25th, 75th centile]		69.5 [31.5, 248]	62 [32, 233]	
Acceptance of index referral	12 months	277/612 (45%)	262/610 (43%)	RR: 1.06 (0.94, 1.19) RD: 2.58 (−2.72, 7.89)
Acceptance of any referral	12 months	349/612 (57%)	337/610 (55%)	RR: 1.03 (0.94, 1.13) RD: 1.97 (−3.31, 7.25)
	18 months	374/611 (61%)	352/608 (58%)	RR: 1.06 (0.97, 1.16) RD: 3.58 (−1.67, 8.83)
Discharge from CAMHS	12 months	125/349 (36%)	122/337 (36%)	
	18 months	195/374 (52%)	172/352 (49%)	
Re‐referral to CAMHS	12 months	174/610 (29%)	155/609 (25%)	
	18 months	227/609 (37%)	201/606 (33%)	
Any treatment/intervention offered for a diagnosed emotional disorder	12 months	33/610 (5%)	38/609 (6%)	RR: 0.87 (0.59, 1.27) RD: −0.93 (−3.49, 1.63)
	18 months	44/609 (7%)	49/606 (8%)	RR: 0.89 (0.66, 1.21) RD: −1.19 (−4.47, 2.10)
Time from randomisation to the decision to offer treatment/intervention for a diagnosed emotional disorder (weeks)	12 months			HR: 0.81 (0.51, 1.30)
Mean [*SD*]		26.2 [14.4]	19.6 [14.5]	
Median [25th, 75th centile]		23 [14, 37]	15 [7, 33]	
*n*		33	38	
	18 months			HR: 0.84 (0.56, 1.27)
Mean [*SD*]		35.5 [20.9]	29.2 [22.3]	
Median [25th, 75th centile]		34 [16, 52]	22 [9, 44]	
*n*		44	49	
Any treatment/intervention started for a diagnosed emotional disorder	12 months	16/610 (3%)	25/609 (4%)	RR: 0.63 (0.36, 1.12) RD: −1.78 (−4.42, 0.87)
	18 months	23/609 (4%)	31/606 (5%)	RR: 0.73 (0.45, 1.17) RD: −1.75 (−4.64, 1.15)
Time from randomisation to start of treatment/intervention for a diagnosed emotional disorder (weeks)	12 months			HR: 0.59 (0.31, 1.10)
Mean[*SD*]		31.1 [14.3]	27.8 [17.2]	
Median [25th, 75th centile]		32 [20, 45]	31 [12, 44]	
*n*		16	25	
Mean [*SD*]	18 months	40.6 [19.3]	35.1 [21.7]	HR: 0.68 (0.39, 1.17)
Median [25th, 75th centile]		43 [28, 53]	38 [13, 51]	
*n*		23	31	
Any treatment/intervention offered	12 months	254/610 (42%)	238/609 (39%)	RR: 1.05 (0.93, 1.18) RD: 2.34 (−3.07, 7.74)
	18 months	292/609 (48%)	278/606 (46%)	RR: 1.03 (0.93, 1.15) RD: 1.73 (−3.62, 7.08)
Time from randomisation to the decision to offer any treatment/intervention (weeks)	12 months			HR: 1.05 (0.88, 1.26)
Mean [*SD*]		19.3 [14.9]	17.1 [13.7]	
Median [25th, 75th centile]		16.5 [6, 32]	13.5 [6, 25]	
*n*		254	238	
Mean [*SD*]	18 months	25.2 [20.9]	23.7 [20.8]	HR: 1.04 (0.88, 1.23)
Median [25th, 75th centile]		19.5 [8, 38.5]	17 [7, 37]	
*n*		292	278	
Any treatment/intervention started	12 months	164/610 (27%)	169/609 (28%)	RR: 0.97 (0.81, 1.16) RD: −0.75 (−5.70, 4.20)
	18 months	198/609 (33%)	195/606 (32%)	RR: 1.01 (0.86, 1.19) RD: 0.39 (−4.76, 5.55)
Time from randomisation to start of any treatment/intervention (weeks)	12 months			HR: 0.95 (0.77, 1.18)
Mean [*SD*]		22.1 [14.4]	20.4 [13.6]	
Median [25th, 75th centile]		20 [10, 33.5]	19 [9, 32]	
*n*		164	169	
Mean [*SD*]	18 months	29.4 [21]	26.1 [19.4]	HR: 1.00 (0.82, 1.21)
Median [25th, 75th centile]		25.5 [12, 44]	21 [10, 39]	
*n*		198	195	

CAMHS, Child and Adolescent Mental Health Services; HR, hazard ratio; RD, risk difference; RR, risk ratio.

^a^ Adjusted by site and other minimisation factors.

**Table 4:** Subgroup analysis of primary outcome

	Intervention group	Control group	Adjusted risk ratio^a^	Adjusted interaction effect^b^	*p*‐Value for interaction
	(*n* = 615)	(*n* = 610)	(95% CI)	(95% CI)	
By sex^c^					
Female	47/355 (13%)	51 /355 (14%)	0.93 (0.65, 1.34)	0.97 (0.42, 2.11)	.891
Male	21/255 (8%)	21/254 (8%)	0.97 (0.56, 1.68)		
By age^d^					
5–10 years	7/212 (3%)	12/209 (6%)	0.65 (0.27, 1.58)	1.62 (0.56, 4.62)	.371
11–17 years	61/398 (15%)	60/400 (15%)	1.01 (0.73, 1.39)		

^a^ Adjusted by site and other minimisation factors.

^b^ Odds ratio taken from interaction term within the model.

^c^ Missing outcome data for one female in the control group, two males and three females in the intervention group.

^d^ Missing outcome data for one 5–10‐year‐old in the control group and five 11–17‐year‐olds in the intervention group.

At 12 months, questionnaire completion was 77% from parents/carers and 62% from CYP, with returns similar between the randomised groups (Figure 1 and Table 5). Mean scores for CYP depression and anxiety symptoms remained high at 12 months in both intervention and control groups, but there was no evidence of any between‐group differences for any of the participant‐reported secondary outcomes (Table 5).

**Table 5:** Secondary outcomes from participants

	Baseline	6 months	12 months
	Mean (*SD*)	Mean (*SD*)	Mean (*SD*)
CYP's symptoms (self‐report)			
Depression symptoms (MFQ)			
Intervention Group (*n* = 252)	38.7 [13.8] (*n* = 237)	36.8 [16] (*n* = 135)	34.2 [15.9] (*n* = 147)
Control Group (*n* = 250)	37.7 [13.3] (*n* = 236)	34.4 [14.9] (*n* = 120)	33 [16.5] (*n* = 146)
Adjusted difference in means^a^ (95% CI)		2.19 (−1.10, 5.48)	−0.06 (−3.51, 3.39)
Anxiety symptoms (RCADS)			
Intervention Group (*n* = 252)	55.8 [21.9] (*n* = 237)	54.2 [23.8] (*n* = 135)	51.6 [24.3] (*n* = 144)
Control Group (*n* = 250)	57.7 [20.6] (*n* = 236)	54.9 [22.2] (*n* = 119)	52.1 [22.8] (*n* = 145)
Adjusted difference in means^a^ (95% CI)		−2.92 (−7.70, 1.87)	−1.80 (−6.52, 2.92)
Oppositional defiant/conduct disorder subscale (SDQ conduct problems)^b^			
Intervention Group (*n* = 252)	3.2 [2.1] (*n* = 150)	3 [2.1] (*n* = 135)	3.2 [2.2] (*n* = 147)
Control Group (*n* = 250)	2.9 [2.1] (*n* = 234)	2.6 [2] (*n* = 125)	2.5 [2] (*n* = 150)
Adjusted difference in means^a^ (95% CI)		0.36 (−0.09, 0.81)	0.41 (−0.03, 0.84)
Functional Impairment (SDQ impact supplement)^b^			
Intervention Group (*n* = 252)	4.9 [2.6] (*n* = 139)	3.8 [2.8] (*n* = 132)	3.8 [3.1] (*n* = 147)
Control Group (*n* = 250)	3.9 [2.5] (*n* = 231)	3.8 [2.8] (*n* = 122)	3.7 [2.9] (*n* = 147)
Adjusted difference in means^a^ (95% CI)		−0.03 (−0.66, 0.59)	0.07 (−0.57, 0.72)
CYP's symptoms (Parent/carer reported)			
Depression symptoms (MFQ)			
Intervention Group (*n* = 560)	31.6 [13.3] (*n* = 556)	25.3 [14.7] (*n* = 404)	23.5 [15.7] (404)
Control Group (*n* = 561)	31.5 [14.1] (*n* = 554)	26.6 [15.4] (*n* = 405)	23.9 [15.2] (*n* = 404)
Adjusted difference in means^a^ (95% CI)		−1.02 (−2.89, 0.86)	−0.30 (−2.29, 1.68)
Anxiety symptoms (RCADS)			
Intervention Group (*n* = 560)	45.3 [19.7] (*n* = 553)	41.2 [21.4] (*n* = 393)	39.3 [22] (*n* = 400)
Control Group (*n* = 561)	46.6 [20.8] (*n* = 548)	42.3 [21.6] (*n* = 391)	39.7 [22.3] (*n* = 403)
Adjusted difference in means^a^ (95% CI)		−1.50 (−4.17, 1.18)	−0.63 (−3.38, 2.11)
Oppositional defiant/conduct disorder subscale (SDQ conduct problems)			
Intervention Group (*n* = 560)	3.5 [2.3] (*n* = 425)	3.3 [2.4] (*n* = 410)	3.3 [2.4] (*n* = 412)
Control Group (*n* = 561)	3.5 [2.5] (*n* = 554)	3.1 [2.4] (*n* = 417)	3.1 [2.5] (*n* = 412)
Adjusted difference in means^a^ (95% CI)		0.09 (−0.21, 0.38)	0.09 (−0.21, 0.39)
Functional Impairment (SDQ impact supplement)			
Intervention Group (*n* = 560)	5.8 [2.8] (*n* = 424)	4.7 [3.1] (*n* = 405)	4.4 [3.2] (*n* = 405)
Control Group (*n* = 561)	4.9 [2.9] (*n* = 551)	4.8 [3.1] (*n* = 410)	4.5 [3.3] (*n* = 406)
Adjusted difference in means^a^ (95% CI)		−0.06 (−0.45, 0.33)	−0.07 (−0.49, 0.34)
Parent/carer self‐reported symptoms			
Depression symptoms (PHQ‐9)			
Intervention Group (*n* = 560)	9.5 [6.6] (*n* = 557)	7.9 [6.1] (*n* = 385)	7.6 [6.2] (*n* = 402)
Control Group (*n* = 561)	9.1 [6.3] (*n* = 555)	8.1 [6.2] (*n* = 388)	7.8 [6.2] (*n* = 401)
Adjusted difference in means^a^ (95% CI)		−0.26 (−1.08, 0.56)	−0.22 (−1.04, 0.60)
Anxiety symptoms (GAD‐7)			
Intervention Group (*n* = 560)	8.7 [6] (*n* = 559)	7 [5.6] (*n* = 383)	6.7 [5.5] (*n* = 400)
Control Group (*n* = 561)	8.3 [5.9] (*n* = 556)	7.2 [5.6] (*n* = 386)	6.7 [5.6] (*n* = 401)
Adjusted difference in means^a^ (95% CI)		−0.16 (−0.92, 0.59)	0.01 (−0.73, 0.75)

CYP, Child/Young Person; GAD‐7, Generalised Anxiety Disorder; MFQ, Mood and Feelings Questionnaire; PHQ‐9, Patient Health Questionnaire‐9; RCADS, Revised Children's Anxiety and Depression Scale.

^a^ Adjusted by site and other minimisation factors.

^b^ Higher proportion of missing data in the intervention group for the Strengths and Difficulties Questionnaire (SDQ) because participants in the intervention group completed the SDQ post‐randomisation as part of the Development and Well‐being Assessment (DAWBA).

Child‐safety outcomes are reported in Appendix S1: Tables 2.35–2.40. At 6 and 12 months, around a quarter of CYP reported a significant deterioration in depression compared to baseline, and 34–41% reported hurting themselves on purpose, at least once, during the previous 6 months.

### Costs and cost effectiveness analyses

Table 6 presents imputed health service and broader societal costs, EQ‐5D‐Y and QALY outcomes and estimated between‐group differences. Both average and estimated between‐group differences found costs and QALYs were not in favour of using the DAWBA intervention, showing higher costs for no improvement in outcome.

**Table 6:** Health economic imputed analysis results

	Intervention group	Control group	Difference (95% confidence interval)
	Mean values	Mean values	
Outcomes			
EQ‐5D‐Y baseline	0.4862	0.4968	−0.0106 (−0.0505, 0.0293)
EQ‐5D‐Y 6 months	0.5679	0.5993	−0.0313 (−0.0742, 0.0116)
EQ‐5D‐Y 12 months	0.6072	0.6269	−0.0197 (−0.0616, 0. 0221)
QALYs	0.5573	0.5805	−0.0232 (−0.0572, 0. 0107)
Estimated QALY differences			−0.0181 (−0.0427, 0.0065)
NHS and PSS costs			
Intervention	£10.00	£0.00	£10.00
Inpatient care	£48.50	£28.58	£19.93 (−18.83, 58.69)
Outpatient care	£901.33	£872.49	£28.84 (−176.06, 233.74)
Community care including social care services	£152.25	£165.32	−£13.06 (−93.17, 67.04)
Primary care	£467.34	£486.42	−£19.07 (−100.69, 62.54)
Medication	£16.81	£11.44	£5.37 (−5.25, 16.00)
Broader costs			
Productivity losses	£638.94	£468.54	£170.40 (−103.42, 444.21)
Over‐the‐counter medication	£15.20	£18.61	−£3.41 (−9.91, 3.09)
Out‐of‐pocket expenses	£706.08	£841.62	−£135.53 (−599.16, 328.09)
Total costs			
Total NHS and PSS costs	£1,596.24	£1,564.23	£32.00 (−251.96, 315.96)
Estimated NHS and PSS cost differences			£28.80 (−249.41, 307.01)
Total broader costs	£1,360.22	£1,328.77	£31.45 (−516.27, 579.17)
Total societal costs (NHS + broader)	£2,956.45	£2,893.00	£63.46 (−618.53, 745.44)
Estimated total societal cost differences			£68.98 (−607.66, 745.62)

See Appendix S1: Table 3.1 for what is encapsulated in NHS and PSS. EQ‐5D‐Y, EuroQol‐5 Dimensions‐Youth; NHS, National Health Service; PSS, personal social services; QALYs, quality‐adjusted life years.

The average imputed EQ‐5D‐Y preference scores were lower in the intervention group at baseline and over the course of the trial compared with the control group. This translated into QALY losses for the intervention group with and without adjustments made for baseline differences and other factors. Average CHU9D preference scores were higher than EQ‐5D‐Y scores but comparable between groups (Appendix S1: Table 3.5). Average imputed costs were slightly higher in the intervention than control group from both an NHS & PSS perspective, and a broader societal perspective (Table 6). The main cost drivers in both arms were in outpatient care costs, productivity losses and out‐of‐pocket expenses. Estimated cost differences were consistent with average imputed results. Between group differences tended to be small, statistically insignificant and subject to a high degree of uncertainty, as illustrated by the cost effectiveness planes (Appendix S1: Figures 3.1 and 3.2). The probability that the intervention was cost‐effective compared to control did not exceed 50% across all cost effectiveness thresholds and costing perspectives considered (Appendix S1: Figures 3.3 and 3.4). Complete case analyses did not alter base conclusions (Appendix S1: Table 3.6). Lower costs in the intervention arm did not compensate for associated health losses at a £20,000 per QALY threshold. A complete set of results tables, as detailed in the Statistical and Health Economic Analysis Plans, are in the Appendices.

## Discussion

In this large, multi‐centre, pragmatic RCT we found no evidence for the clinical or cost effectiveness for this method of integrating an SDA tool into usual clinical care for CYP with emotional difficulties referred to CAMHS – this was despite considerable efforts to encourage clinicians to view the DAWBA report and consider its findings as part of assessment and diagnosis. There was no discernible effect on the primary outcome (clinical diagnosis of an emotional disorder) or secondary outcomes (either participant‐reported or service‐related). There are several possible reasons why this might have been the case. First, 20% did not complete the intervention. Second, the intervention involving the completion of an online assessment tool was perhaps too ‘light touch’ to change clinician behaviour, particularly as the intervention was completed at the level of the parent and/or young person but its impact assessed at the level of a different individual (i.e. the clinician). Third, the DAWBA report was added to the clinical records soon after referral receipt and although clinicians in the Single/Central Point of Access triage teams were informed that it was available for their review, it may not have been seen by the subsequent (more downstream) clinician carrying out the clinical assessment, often many months later. We did not collect data on clinicians' access and use of the DAWBA report and therefore cannot confirm the extent to which they viewed and used the report. Fourth, referral acceptance was only 56% by 12 months which limited the opportunity to receive a diagnosis from CAMHS; nevertheless, the pattern of findings was consistent across primary and secondary outcomes. In terms of the economic evaluation, we found no evidence to suggest that the intervention impacted health‐service utilisation, broader societal costs or quality of life outcomes for CYP or their parents/carers.

Although previous trials (Aebi et al., 2012; Ford et al., 2013; Hansen et al., 2023) have suggested that SDA tools might have potential in routine care, these were relatively small studies based in single sites and local contextual factors may limit their generalisability to a wider range of CAMHS settings. The two previous RCTs (Aebi et al., 2012; Ford et al., 2013) focused on comparisons of agreement levels between DAWBA and clinician‐made diagnoses. In contrast, the STADIA trial was situated across eight large NHS Trusts enabling a broad real‐world evaluation of introducing an SDA tool at the point of referral receipt. Several aspects of our findings are particularly noteworthy. First, clinical diagnosis rates were much lower than expected and not dissimilar to community prevalence rates (Sadler et al., 2018). One might expect higher rates of disorder in CYP referred to secondary care CAMHS compared with population samples, which is borne out by the finding that 67% of those completing the DAWBA scored very high for at least one emotional disorder, particularly depression and generalised anxiety. Although our pre‐trial data suggested an expected clinical diagnosis rate of 45%, this figure was based on service evaluation or audit data which did not necessarily reflect the use of precise diagnostic terminology consistent with ICD and DSM classification systems, as used in the trial. It is of note, however, that when combining the firm and uncertain diagnoses in our study, these total 34–39% at 12 months and 39–43% at 18 months, closer to the originally expected figure. It is possible that the use of the DAWBA contributed to a shift from no diagnostic information being recorded to an attempt to record some information, albeit these being uncertain or unclear.

Second, the intervention was situated at the point of referral receipt, in line with recommendations from previous research (Ford et al., 2013; Reeves et al., 2016). Referral acceptance was therefore a proximal outcome in the care pathway timeline and, although there was no significant effect, there was a suggestion of a consistent pattern that the DAWBA might have had a very small influence (by 3–6%) on increasing the likelihood of acceptance of the index referral or of any referral by 12 or 18 months.

Third, our findings highlight the very high levels of need experienced by our sample in terms of severity of mental health difficulties and associated functional impairment. Despite this, only 44% of index referrals were accepted, and over one‐third of our sample were re‐referred to CAMHS over the 18‐month period, resulting in delays in receiving help. Less than half the sample were offered any treatment/intervention during the 18‐month follow‐up period. Even though some regression to the mean might be expected, particularly given that referrals are most likely to be made at a point of greatest severity, it is striking that mental health difficulties remained persistent at 12‐month follow‐up. Our sample had high levels of self‐and parent‐reported mental health symptoms, functional impairment, and self‐harm thoughts and behaviour, even at 12‐month follow‐up. These findings suggest that current CAMHS provision, availability and capacity appear to be insufficient to meet clinical demand and need.

In terms of methodological issues, our operationalisation of the primary outcome adopted a strict approach whereby clinically recorded diagnoses had to match ICD or DSM diagnostic terminology, for example, requiring the term ‘disorder’ as a suffix for different types of anxiety difficulties, for example, separation anxiety. There was a suggestion that clinicians often used diagnostic terminology imprecisely, for example, not using the term ‘disorder’. These descriptions were categorised as being uncertain diagnoses and all such diagnoses were subjected to adjudication. The proportion of uncertain diagnoses was 28% in the intervention arm and 22% in the control arm at 12 months. This might reflect that the DAWBA report was careful not to imply that diagnostic criteria were fully met and used language accordingly, highlighting that the ratings were a guide to the level of difficulties present for each assessed domain.

In terms of wider learning from the trial, our data highlight that 80% of participants completed the DAWBA suggesting that it was acceptable to a diverse sample of families. Almost all participants completed the DAWBA online rather than taking up the option of completion by telephone. These findings suggest that online/digital approaches to diagnostic assessment are highly acceptable to families and young people who have been referred to CAMHS.

To our knowledge, this is one of the largest RCTs involving CYP who have been referred to mental health services and has a number of notable strengths. This rigorously conducted trial had a national spread of sites with good geographical representation across England to ensure diversity and inclusion. Our sample was representative of the target population of CYP seen by CAMHS in terms of demographics, including sex and ethnicity (Edbrooke‐Childs, Rashid, Ritchie, & Deighton, 2022). Trial procedures were conducted remotely, enabling recruitment and retention to continue throughout the COVID‐19 pandemic period. Follow‐up and retention rates were excellent, with primary outcome data collected for >99% of participants. Adherence to the intervention was good (80%) and participant‐reported questionnaire completion remained high at 6‐ and 12‐month post‐randomisation. Qualitative data from the process evaluation (manuscript under review) showed that young people and parents/carers valued the information provided by the DAWBA report and showed high levels of engagement with the intervention and research study. Crucially, PPI was very strongly embedded throughout the life cycle of the research.

In terms of limitations, we were unable to mask participants, researchers collecting source data or clinicians to treatment allocation; however, we did have robust adjudication procedures in place. Due to the pragmatic nature of the trial we did not ask clinicians to record whether they saw the DAWBA report in the CAMHS clinical records. As we did not measure this, we do not know how many assessing/diagnosing clinicians looked at the DAWBA report and therefore there is uncertainty about the extent to which the DAWBA reports were reviewed by assessing/diagnosing clinicians. The complexity of the electronic clinical records (multiple progress notes/running records tabs and correspondence and letters) may have decreased visibility of the DAWBA report. We did, however, ensure that site researchers informed clinicians in the Single/Central Point of Access triage teams that the report was available for their review and added periodic reminders into the clinical records, in addition to regular presentations from the site Principal Investigators to their local CAMHS teams to maintain awareness of the trial, engage clinicians and remind them to check for DAWBA reports in the clinical records.

Overall, recorded clinical diagnosis rates were similar to prevalence rates in the community (Sadler et al., 2018), which suggests clinical under‐diagnosis and possible under‐recognition of emotional disorders. Through qualitative interviews, we identified clinician reluctance to make diagnoses (manuscript under review). Our findings have important implications for research – if clinical diagnoses are not made and recorded, it is challenging to rely on clinical records to study treatment response and outcomes in routine care. NICE clinical guidelines for the assessment and treatment of various emotional disorders are based on diagnostic categories. However, from an individual patient perspective, it remains unclear whether receipt of a clinical diagnosis matters and makes a difference to clinical outcomes. It is possible that the same type of help might be received regardless of whether a diagnosis of emotional disorder is made.

These findings, in the context of the SDA, show a high prevalence of likely emotional disorders in CYP referred to CAMHS. Some CYP started interventions for an emotional disorder, despite not having a confirmed diagnosis. This may reflect the controversy around the use of diagnoses amongst CAMHS clinicians, and/or the grade, training and professional background of clinicians carrying out assessments and treatment. The intervention seems to have been insufficient to enable clinician behaviour change, particularly in terms of views towards making, sharing and recording diagnosis in the clinical records. The results from this study suggest that more intensive in‐service training and continuing professional development approaches are needed to ensure effective training and maintenance of clinician engagement. In terms of implications for clinicians, service funders and policymakers, although the provision and completion of a remotely offered SDA tool (the DAWBA) is a relatively inexpensive intervention and acceptable for young people and their parents/carers, when administered at the point of referral receipt and delivered in the format used in this trial, there was no evidence for its effectiveness or cost effectiveness. However, it should be noted that this is within the context of services with a high threshold for referral acceptance which therefore limits the opportunities for CYP to be offered treatment/intervention by CAMHS.

## Conclusion

Despite some promising evidence from previous single‐site studies, the findings from this pragmatic trial do not support the wider roll‐out and implementation of SDA tools, in the format as delivered here, in the assessment of child and youth emotional disorders in routine practice in community‐based multi‐disciplinary CAMHS settings. However, we did not collect data on clinician usage of the DAWBA report and therefore cannot confirm or refute the extent to which it was considered by clinicians within the assessment and diagnosis process. Our findings should also be interpreted within the context of services which have a high threshold for accepting referrals.

## Ethical considerations

Ethical approval was obtained from the South Birmingham Research Ethics Committee (Ref. 19/WM/0133); all participants gave informed consent before taking part.

## Trial registration

The trial was prospectively registered as ISRCTN15748675.

**Key points**

- There is clinician uncertainty about the value of diagnosing emotional disorders and the use of standardised diagnostic assessment (SDA) tools in routine practice.
- Mixed findings from single‐site studies have highlighted the need for a definitive effectiveness and cost effectiveness multi‐site RCT to investigate whether an SDA tool improves the detection of emotional disorders and outcomes in children and young people.
- Online/digital approaches to diagnostic assessment are highly acceptable to families and young people who have been referred to CAMHS.
- Although a remotely‐delivered SDA intervention offered at the point of referral receipt by CAMHS was well accepted by young people and parents/carers, this study found no evidence for its effectiveness or cost effectiveness.
- The findings from this trial do not support the roll‐out and routine implementation of SDA tools at the point of referral receipt in the assessment of child and youth emotional disorders in multi‐disciplinary CAMHS settings, in services with a high threshold for referral acceptance.

## Supporting information


Appendix S1
**Table 1.1.** Table of recruiting sites.
**Table 1.2.** Changes to the protocol since the start of the study.
**Table 1.3.** Consent and participation.
**Table 1.4.** Outcome definition and adjudication plan.
**Table 1.5.** Summary of assessments.
**Table 1.6.** Eligible emotional disorder diagnoses.
**Table 1.7.** Secondary Outcome Measures.
**Table 2.1.** Analysis set definitions and numbers in each group.
**Table 2.2.** Trial recruitment by intervention arm and participating site.
**Table 2.3.** Child baseline characteristics data (minimisation factors highlighted).
**Table 2.4.** Parent/carer baseline characteristics data.
**Table 2.5.** Child baseline assessment data (self‐reported).
**Table 2.6.** Child baseline assessment data (parent/carer‐reported).
**Table 2.7.** Parent/carer self‐report baseline assessment data.
**Table 2.8.** Child self‐harm (self‐report) at baseline.
**Table 2.9.** Process outcomes.
**Table 2.10.** Adherence to intervention.
**Table 2.11.** DAWBA Summary.
**Table 2.12.** Summary of baseline and follow up questionnaires for child/young person.
**Table 2.13.** Summary of baseline and follow up questionnaires for parent/carer.
**Table 2.14.** Completeness of baseline and follow up questionnaires for child/young person (self‐reported – 11+).
**Table 2.15.** Completeness of baseline and follow up questionnaires for child/young person (parent/carer‐reported).
**Table 2.16.** Completeness of baseline and follow up questionnaires for parent/carers.
**Table 2.17.** Completeness of health economics questionnaires for child/young person (self‐ or parent/carer‐ completed).
**Table 2.18.** Primary outcome.
**Table 2.19.** Subgroup analysis of primary outcome.
**Table 2.20.** Secondary analysis of primary outcome (ordinal analysis).
**Table 2.21.** Clinician‐made diagnosis decision about the presence of an emotional disorder within 18 months of randomisation.
**Table 2.22.** Secondary outcomes – referral acceptance within 12 months.
**Table 2.23.** Secondary outcomes – referral acceptance within 18 months.
**Table 2.24.** Secondary outcomes – treatment within 12 months.
**Table 2.25.** Secondary outcomes – treatment within 18 months.
**Table 2.26.** Child symptoms and functional impairment (self‐reported).
**Table 2.27.** Child symptoms and functional impairment (parent/carer‐reported).
**Table 2.28.** Parent/carer self‐reported outcomes.
**Table 2.29.** Secondary outcomes for the child – other.
**Table 2.30.** Imputation of Child symptoms and functional impairment (self‐reported).
**Table 2.31.** Imputation of Child symptoms and functional impairment (parent/carer‐reported).
**Table 2.32.** Imputation of Parent/carer self‐reported outcomes.
**Table 2.33.** Post‐traumatic stress disorder symptoms in the child/young person (self‐reported).
**Table 2.34.** Post‐traumatic stress disorder symptoms in the child/young person (parent/carer‐reported).
**Table 2.35.** Summary of child safety outcomes (self‐report).
**Table 2.36.** Summary of child safety outcomes (parent/carer reported).
**Table 2.37.** Summary of child safety outcomes (reported by either child or parent/carer).
**Table 2.38.** Summary of parent/carer self‐reported safety outcomes.
**Table 2.39.** Summary of safety outcomes from records within 12 months.
**Table 2.40.** Summary of safety outcomes from records within 18 months.
**Table 2.41.** Withdrawal summary.
**Table 2.42.** Adherence to intervention (self‐reported DAWBA – 11–15).
**Table 2.43.** Adherence to intervention (self‐reported DAWBA – 16–17).
**Table 2.44.** Adherence to intervention (parent/carer‐reported DAWBA).
**Table 2.45.** Secondary outcomes – diagnoses of emotional disorders from records within 12 and 18 months.
**Table 2.46.** Secondary outcomes – child/young person and parent/carer reported diagnoses within 12 months.
**Table 2.47.** Secondary outcomes – Treatments offered for a diagnosed emotional disorder within 12 and 18 months.
**Table 2.48.** Secondary outcomes – Treatments/interventions started for a diagnosed emotional disorder within 12 and 18 months.
**Table 2.49.** Secondary outcomes – Any treatments/interventions offered within 12 and 18 months.
**Table 2.50.** Secondary outcomes – Any treatments/interventions started within 12 and 18 months.
**Table 2.51.** Secondary outcomes – Medications offered* for an emotional disorder within 12 and 18 months.
**Table 2.52.** Secondary outcomes – Any medications offered* within 12 and 18 months.
**Table 2.53.** Protocol deviations.
**Table 3.1.** Resource use and cost sources.
**Table 3.2.** Unit cost.
**Table 3.3.** Standard occupational classifications and costs (ONS, 2021) [18].
**Table 3.4.** Missingness in costs and outcomes.
**Table 3.5.** Base case outcome table.
**Table 3.6.** Complete case results.
**Table 3.7.** Base case seemingly‐unrelated regression analysis (NHS & PSS cost perspective).
**Table 3.8.** Broader societal seemingly‐unrelated regression analysis.
**Table 3.9.** HEAP deviations.
**Table 3.10.** Medication unit costs.
**Figure 1.1.** Screening forms.
**Figure 1.2.** Participant flow.
**Figure 1.3.** DAWBA report template.
**Figure 2.1.** CONSORT flow diagram.
**Figure 2.2.** Time to first diagnosis of an emotional disorder within 12 months.
**Figure 2.3.** Time to first diagnosis of an emotional disorder within 18 months.
**Figure 2.4.** Time to first offered treatment/intervention for an emotional disorder within 12 months.
**Figure 2.5.** Time to start of first treatment/intervention for an emotional disorder within 12 months.
**Figure 2.6.** Time to first starting any treatment/intervention within 12 months.
**Figure 2.7.** Time to first offered any treatment/intervention within 12 months.
**Figure 2.8.** Time to first offered treatment/intervention for an emotional disorder within 18 months.
**Figure 2.9.** Time to start of first treatment/intervention for an emotional disorder within 18 months.
**Figure 2.10.** Time to first offered any treatment/intervention within 18 months.
**Figure 2.11.** Time to first starting any treatment/intervention within 18 months.
**Figure 3.1.** Base case cost‐effectiveness scatter plot.
**Figure 3.2.** Societal perspective scatter plot.
**Figure 3.3.** Base case Cost‐Effectiveness Acceptability Curve.
**Figure 3.4.** Societal perspective Cost‐Effectiveness Acceptability Curve.

## Data Availability

Anonymised trial data may be shared with researchers external to the trial research team in accordance with the NCTU's data sharing procedure. The datasets containing individual participant data analysed during the STADIA trial will be available upon request from the NCTU (ctu@nottingham.ac.uk) a minimum of 6 months after publication of the NIHR threaded publication. Access to the data will be subject to review of a data sharing and use request by a committee including the Chief Investigator and Sponsor, and should reflect a proposed collaboration with the STADIA trial team, and will only be granted upon receipt of a data sharing and use agreement. Any data shared will be anonymised which may impact on the reproducibility of published analyses.
