# Supplementary material for: The clinical and cost effectiveness of a STAndardised DIagnostic Assessment for children and adolescents with emotional difficulties: the STADIA multi‐centre randomised controlled trial
Source: J Child Psychol Psychiatry. 2025 Jan 7;66(6):805–20. doi: 10.1111/jcpp.14090 (PMC12062850; doi:10.1111/jcpp.14090)
Supplement: Supplementary file 1 — Appendix S1 Table 1.1. Table of recruiting sites. Table 1.2. Changes to the protocol since the start of the study. Table 1.3. Consent and participation. Table 1.4. Outcome definition and adjudication plan. Table 1.5. Summary of assessments. Table 1.6. Eligible emotional disorder diagnoses. Table 1.7. Secondary Outcome Measures. Table 2.1. Analysis set definitions and numbers in each group. Table 2.2. Trial recruitment by intervention arm and participating site. Table 2.3. Child baseline characteristics data (minimisation factors highlighted). Table 2.4. Parent/carer baseline characteristics data. Table 2.5. Child baseline assessment data (self‐reported). Table 2.6. Child baseline assessment data (parent/carer‐reported). Table 2.7. Parent/carer self‐report baseline assessment data. Table 2.8. Child self‐harm (self‐report) at baseline. Table 2.9. Process outcomes. Table 2.10. Adherence to intervention. Table 2.11. DAWBA Summary. Table 2.12. Summary of baseline and follow up questionnaires for child/young person. Table 2.13. Summary of baseline and follow up questionnaires for parent/carer. Table 2.14. Completeness of baseline and follow up questionnaires for child/young person (self‐reported – 11+). Table 2.15. Completeness of baseline and follow up questionnaires for child/young person (parent/carer‐reported). Table 2.16. Completeness of baseline and follow up questionnaires for parent/carers. Table 2.17. Completeness of health economics questionnaires for child/young person (self‐ or parent/carer‐ completed). Table 2.18. Primary outcome. Table 2.19. Subgroup analysis of primary outcome. Table 2.20. Secondary analysis of primary outcome (ordinal analysis). Table 2.21. Clinician‐made diagnosis decision about the presence of an emotional disorder within 18 months of randomisation. Table 2.22. Secondary outcomes – referral acceptance within 12 months. Table 2.23. Secondary outcomes – referral acceptance within 18 months. Table 2.24. Secondary outcomes – treatment within [file JCPP-66-805-s001.docx]

**APPENDIX**

**List of Tables**

[Table 1.1: Table of recruiting sites 4](#_Toc184824950)

[Table 1.2: Changes to the protocol since the start of the study 4](#_Toc184824951)

[Table 1.3: Consent and participation 17](#_Toc184824952)

[Table 1.4: Outcome definition and adjudication plan 18](#_Toc184824953)

[Table 1.5: Summary of assessments 22](#_Toc184824954)

[Table 1.6: Eligible emotional disorder diagnoses 25](#_Toc184824955)

[Table 1.7: Secondary Outcome Measures 26](#_Toc184824956)

[**Table 2.1: Analysis set definitions and numbers in each group** 35](#_Toc184824957)

[Table 2.2: Trial recruitment by intervention arm and participating site 35](#_Toc184824958)

[**Table 2.3: Child baseline characteristics data (minimisation factors highlighted)** 36](#_Toc184824959)

[**Table 2.4: Parent/carer baseline characteristics data** 37](#_Toc184824960)

[Table 2.5: Child baseline assessment data (self-reported) 38](#_Toc184824961)

[Table 2.6: Child baseline assessment data (parent/carer-reported) 40](#_Toc184824962)

[Table 2.7: Parent/carer self-report baseline assessment data 43](#_Toc184824963)

[Table 2.8: Child self-harm (self-report) at baseline 43](#_Toc184824964)

[Table 2.9: Process outcomes 44](#_Toc184824965)

[**Table 2.10: Adherence to intervention** 45](#_Toc184824966)

[**Table 2.11: DAWBA Summary** 46](#_Toc184824967)

[**Table 2.12: Summary of baseline and follow up questionnaires for child/young person** 47](#_Toc184824968)

[**Table 2.13: Summary of baseline and follow up questionnaires for parent/carer** 48](#_Toc184824969)

[**Table 2.14: Completeness of baseline and follow up questionnaires for child/young person (self-reported – 11+)** 49](#_Toc184824970)

[**Table 2.15: Completeness of baseline and follow up questionnaires for child/young person (parent/carer-reported)** 49](#_Toc184824971)

[**Table 2.16: Completeness of baseline and follow up questionnaires for parent/carers** 49](#_Toc184824972)

[**Table 2.17: Completeness of health economics questionnaires for child/young person (self- or parent/carer- completed)** 50](#_Toc184824973)

[Table 2.18: Primary outcome 51](#_Toc184824974)

[Table 2.19: Subgroup analysis of primary outcome 52](#_Toc184824975)

[Table 2.20: Secondary analysis of primary outcome (ordinal analysis) 53](#_Toc184824976)

[Table 2.21: Clinician-made diagnosis decision about the presence of an emotional disorder within 18 months of randomisation 54](#_Toc184824977)

[Table 2.22: Secondary outcomes – referral acceptance within 12 months 55](#_Toc184824978)

[**Table 2.23: Secondary outcomes – referral acceptance within 18 months** 56](#_Toc184824979)

[Table 2.24: Secondary outcomes – treatment within 12 months 57](#_Toc184824980)

[Table 2.25: Secondary outcomes – treatment within 18 months 59](#_Toc184824981)

[Table 2.26: Child symptoms and functional impairment (self-reported) 61](#_Toc184824982)

[Table 2.27: Child symptoms and functional impairment (parent/carer-reported) 63](#_Toc184824983)

[Table 2.28: Parent/carer self-reported outcomes 65](#_Toc184824984)

[Table 2.29: Secondary outcomes for the child – other* 65](#_Toc184824985)

[Table 2.30: Imputation of Child symptoms and functional impairment (self-reported) 66](#_Toc184824986)

[Table 2.31: Imputation of Child symptoms and functional impairment (parent/carer-reported) 67](#_Toc184824987)

[Table 2.32: Imputation of Parent/carer self-reported outcomes 67](#_Toc184824988)

[Table 2.33: Post-traumatic stress disorder symptoms in the child/young person (self-reported) 68](#_Toc184824989)

[Table 2.34: Post-traumatic stress disorder symptoms in the child/young person (parent/carer-reported) 68](#_Toc184824990)

[Table 2.35: Summary of child safety outcomes (self-report) 68](#_Toc184824991)

[Table 2.36: Summary of child safety outcomes (parent/carer reported) 69](#_Toc184824992)

[Table 2.37: Summary of child safety outcomes (reported by either child or parent/carer)** 69](#_Toc184824993)

[Table 2.38: Summary of parent/carer self-reported safety outcomes 69](#_Toc184824994)

[Table 2.39: Summary of safety outcomes from records within 12 months 69](#_Toc184824995)

[Table 2.40: Summary of safety outcomes from records within 18 months* 70](#_Toc184824996)

[**Table 2.41: Withdrawal summary** 70](#_Toc184824997)

[Table 2.42: Adherence to intervention (self-reported DAWBA – 11-15) 71](#_Toc184824998)

[Table 2.43: Adherence to intervention (self-reported DAWBA – 16-17) 72](#_Toc184824999)

[Table 2.44: Adherence to intervention (parent/carer-reported DAWBA) 73](#_Toc184825000)

[Table 2.45: Secondary outcomes – diagnoses of emotional disorders from records within 12 and 18 months 74](#_Toc184825001)

[Table 2.46: Secondary outcomes – child/young person and parent/carer reported diagnoses within 12 months 75](#_Toc184825002)

[Table 2.47: Secondary outcomes – Treatments offered for a diagnosed emotional disorder within 12 and 18 months 76](#_Toc184825003)

[Table 2.48: Secondary outcomes – Treatments/interventions started for a diagnosed emotional disorder within 12 and 18 months 77](#_Toc184825004)

[Table 2.49: Secondary outcomes – Any treatments/interventions offered within 12 and 18 months 78](#_Toc184825005)

[Table 2.50: Secondary outcomes – Any treatments/interventions started within 12 and 18 months 80](#_Toc184825006)

[Table 2.51: Secondary outcomes – Medications offered* for an emotional disorder within 12 and 18 months 81](#_Toc184825007)

[Table 2.52: Secondary outcomes – Any medications offered* within 12 and 18 months 82](#_Toc184825008)

[Table 2.53: Protocol deviations 87](#_Toc184825009)

[Table 3.1: Resource use and cost sources 88](#_Toc184825010)

[Table 3.2: Unit cost 90](#_Toc184825011)

[Table 3.3: Standard occupational classifications and costs (ONS, 2021) [18] 91](#_Toc184825012)

[Table 3.4: Missingness in costs & outcomes 93](#_Toc184825013)

[Table 3.5: Base case outcome table 94](#_Toc184825014)

[Table 3.6: Complete case results 95](#_Toc184825015)

[Table 3.7: Base case seemingly-unrelated regression analysis (NHS & PSS cost perspective) 100](#_Toc184825016)

[Table 3.8: Broader societal seemingly-unrelated regression analysis 101](#_Toc184825017)

[Table 3.9: HEAP deviations 102](#_Toc184825018)

[Table 3.10: Medication unit costs 103](#_Toc184825019)

**List of figures**

[Figure 1.1: Screening forms 4](#_Toc184825020)

[Figure 1.2: Participant flow 15](#_Toc184825021)

[Figure 1.3: DAWBA report template 23](#_Toc184825022)

[Figure 2.1: Participant flow diagram (CONSORT) 34](#_Toc184825023)

[Figure 2.2: Time to first diagnosis of an emotional disorder within 12 months 52](#_Toc184825024)

[Figure 2.3: Time to first diagnosis of an emotional disorder within 18 months 55](#_Toc184825025)

[Figure 2.4: Time to first offered treatment/intervention for an emotional disorder within 12 months 83](#_Toc184825026)

[Figure 2.5: Time to start of first treatment/intervention for an emotional disorder within 12 months 83](#_Toc184825027)

[Figure 2.6: Time to first offer of any treatment/intervention within 12 months 84](#_Toc184825028)

[Figure 2.7: Time to first starting any treatment/intervention within 12 months 84](#_Toc184825029)

[Figure 2.8: Time to first offered treatment/intervention for an emotional disorder within 18 months 85](#_Toc184825030)

[Figure 2.9: Time to start of first treatment/intervention for an emotional disorder within 18 months 85](#_Toc184825031)

[Figure 2.10: Time to first offered any treatment/intervention within 18 months 86](#_Toc184825032)

[Figure 2.11: Time to first starting any treatment/intervention within 18 months 86](#_Toc184825033)

[Figure 3.1: Base case cost-effectiveness scatter plot 97](#_Toc184825034)

[Figure 3.2: Societal perspective scatter plot 98](#_Toc184825035)

[Figure 3.3: Base case Cost-Effectiveness Acceptability Curve 98](#_Toc184825036)

[Figure 3.4: Societal perspective Cost-Effectiveness Acceptability Curve 99](#_Toc184825037)

# STADIA Trial

Table 1.1: Table of recruiting sites

| **Recruiting site** | **Principal Investigator** |
| --- | --- |
| Nottinghamshire Healthcare NHS Foundation Trust | Kapil Sayal (also the Chief Investigator for the trial) |
| Berkshire Healthcare NHS Foundation Trust | Tamsin Marshall |
| Cambridgeshire and Peterborough NHS Foundation Trust | Anupam Bhardwaj |
| Central and North West London NHS Foundation Trust | Julia Gledhill |
| Pennine Care NHS Foundation Trust | Bernadka Dubicka |
| Gloucestershire Health and Care NHS Foundation Trust | Helen Bould |
| Surrey and Borders Partnership NHS Foundation Trust | Asifa Zainab |
| Rotherham Doncaster and South Humber NHS Foundation Trust | Abdullah Kraam (Jun 2021-Jul 2022)  Michael Seneviratne (from Jul 2022) |

Table 1.2: Changes to the protocol since the start of the study

| **Protocol** | **Date** | **Summary of changes** |
| --- | --- | --- |
| 2.0 | 16-Dec-2019 | - Confirmation of re-consent procedure for 16 year olds during follow-up. - Clarification regarding outcome definitions. - Minor corrections and clarifications throughout. |
| 3.0 | 13-Aug-2020 | - Additional participant questionnaire (CRIES-8). |
| 4.0 | 03-Feb-2021 | - Implementation of Study Within A Trial (SWAT) and amendment to arrangements to voucher payments to participants. |
| 4.1 | 01-Aug-2022 | - Extension of data collection up to 18-months post-randomisation. - Minor updates to administrative information - Addition of eligible emotional disorder (trichotillomania) |

Figure 1.1: Screening forms


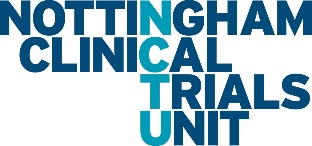


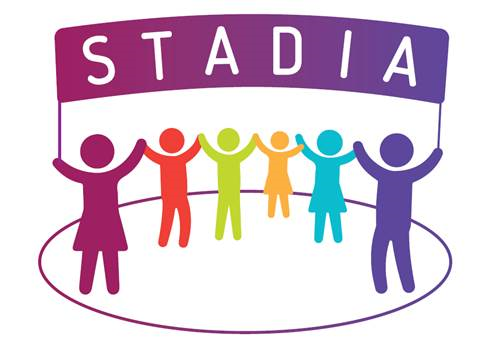


**STAndardised DIagnostic Assessment for children and adolescents with emotional difficulties (STADIA): a multi-centre randomised controlled trial**

**SCREENING**

| **Site Number:** | \|  \|  \| \| --- \| --- \| \|  \|  \| |
| --- | --- | --- | --- | --- | --- |
| **Screening Number:** | \|  \|  \|  \| \| --- \| --- \| --- \| \|  \|  \|  \| |
| **Sponsor:** | **Nottinghamshire Healthcare NHS Foundation Trust** |
| **CRF Version:** | **Final v1.0 – 30 May 2019** |

| **REFERRAL SCREENING** | | | |
| --- | --- | --- | --- |
| *Complete for all referrals screened for eligibility:* | | | |
| NHS Number  *Local use only* |  | | |
| Trust Number  *Local use only* |  | | |
| Date of referral receipt  (dd-mmm-yyyy) | \|  \|  \|  \|  \|  \|  \|  \|  \|  \|  \|  \| \| --- \| --- \| --- \| --- \| --- \| --- \| --- \| --- \| --- \| --- \| --- \| \|  \|  \| - \|  \|  \|  \| - \|  \|  \|  \|  \| | | |
| Date of screening  (dd-mmm-yyyy) | \|  \|  \|  \|  \|  \|  \|  \|  \|  \|  \|  \| \| --- \| --- \| --- \| --- \| --- \| --- \| --- \| --- \| --- \| --- \| --- \| \|  \|  \| - \|  \|  \|  \| - \|  \|  \|  \|  \| | | |
| Young person’s sex | | Male  Female |  |
| Young person’s age  *If <5 or >17 do not proceed* | | \|  \|  \| \| --- \| --- \| \|  \|  \| | |
| Has the young person been previously enrolled and randomised in the STADIA trial?  *If yes, do not proceed* | | Yes  No | |
| Does the referral mention emotional difficulties*?  *If no, do not proceed* | | Yes  No | |
| Is this an emergency or urgent referral (according to local CAMHS triage / SPA team risk assessment)?  *If yes, do not proceed* | | Yes  No | |
| Does the young person have severe learning disability (e.g., the referral mentions this or that they attend a special school for children with severe learning difficulties)?  *If ‘yes’ do not proceed*  *If not known, confirm during telephone eligibility check at enrolment* | | Yes  No  Not known | |
| If the young person is <16:  Does the referral information include contact details for a named parent/carer?  *If ‘no’ await parent/carer contact details before proceeding* | | Yes  No  N/A | |
| If the young person is <16:  Is the named parent/carer a local authority representative designated to care for the child/young person?  *If ‘yes’ do not proceed*  *If not known, confirm during telephone eligibility check at enrolment* | | Yes  No  Not known  N/A | |
| If the young person is aged 16 or 17:  Whose contact details are given on the referral form?  *If young person contact details are provided, they should be contacted in the first instance* | | Young person  Parent/carer  Both  N/A | |

| **EMOTIONAL DIFFICULTIES** | |
| --- | --- |
| **Emotional difficulties may be indicated by the use of any of the following key words or phrases.*  *Tick all that apply. If ‘other’ record details and seek advice from the PI or NCTU before proceeding.* | |
| None |  |
| Agitated / agitation |  |
| Anger |  |
| Anxiety / anxious / generalised anxiety |  |
| Avoids things/people/places |  |
| Can’t leave the house |  |
| Completing rituals / asking parents to carry out rituals |  |
| Compulsions |  |
| Depressed / depression / low / low mood / sad |  |
| Difficulties sleeping |  |
| Distress |  |
| Fears and worries / fears relating to safety (germs, fire) |  |
| Feeling low |  |
| Feels flat / empty / blank |  |
| Feels hopeless |  |
| Feels worthless / stupid |  |
| Flashbacks |  |
| Hypervigilance |  |
| Irritable |  |
| Low motivation |  |
| Low self-esteem / Hates self |  |
| Mood swings / moody |  |
| Negative thoughts |  |
| Nightmares (if trauma also present) |  |
| No (or loss of) energy |  |
| No (or loss of) interest in things / gave up… / lack of wanting to do things |  |
| Not going to school / unable to go to school |  |
| Not sleeping / poor sleep |  |
| Obsessions |  |
| OCD |  |
| Phobia |  |
| Panic / panic attacks |  |
| PTSD |  |
| Self-harm / DSH / Cutting |  |
| Suicidal |  |
| Suicidal thoughts / thoughts of ending life / thinks about killing self |  |
| Tearful |  |
| Thoughts of death |  |
| Tiredness / fatigue |  |
| Touching objects |  |
| Trauma |  |
| Weepy |  |
| Withdrawal / withdrawn |  |
| Worried / worrying (incl. worries/concerns about their appearance |  |
| Other (please specify) |  |

| *FOR ALL REFERRALS SCREENED, ENTER SUMMARY DATA ON THE SCREENING & ENROLMENT LOG.*  *IF THE YOUNG PERSON APPEARS TO BE ELIGIBLE PROCEED TO THE INVITATION TELEPHONE CALL (CALL 1) AND ENTER DETAILS ON THE TRIAL DATABASE.* |
| --- |

| **SIGN-OFF STATEMENT** | |
| --- | --- |
| *Completed by the researcher conducting the referral screening.* | |
| To the best of my knowledge, I confirm that I have made every reasonable effort to ensure that ALL of the data in this Case Record Form is a true, accurate and complete report. | |
| Print Name |  |
| Signature |  |
| Date | \|  \|  \|  \|  \|  \|  \|  \|  \|  \|  \|  \| \| --- \| --- \| --- \| --- \| --- \| --- \| --- \| --- \| --- \| --- \| --- \| \|  \|  \| - \|  \|  \|  \| - \|  \|  \|  \|  \| |


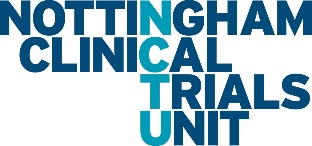


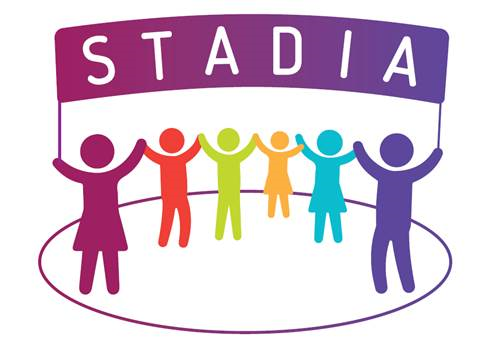


**STAndardised DIagnostic Assessment for children and adolescents with emotional difficulties (STADIA): a multi-centre randomised controlled trial**

**SCREENING**

| **Site Number:** | \|  \|  \| \| --- \| --- \| \|  \|  \| |
| --- | --- | --- | --- | --- | --- |
| **Screening Number:** | \|  \|  \|  \| \| --- \| --- \| --- \| \|  \|  \|  \| |
| **Sponsor:** | **Nottinghamshire Healthcare NHS Foundation Trust** |
| **CRF Version:** | **Final v1.1 – 30 April 2020** |

| **REFERRAL SCREENING** | | |
| --- | --- | --- |
| *Complete for all referrals screened for eligibility:* | | |
| NHS Number  *Local use only* |  | |
| Trust Number  *Local use only* |  | |
| Date of referral receipt  (dd-mmm-yyyy) | \|  \|  \|  \|  \|  \|  \|  \|  \|  \|  \|  \| \| --- \| --- \| --- \| --- \| --- \| --- \| --- \| --- \| --- \| --- \| --- \| \|  \|  \| - \|  \|  \|  \| - \|  \|  \|  \|  \| | |
| Date of screening  (dd-mmm-yyyy) | \|  \|  \|  \|  \|  \|  \|  \|  \|  \|  \|  \| \| --- \| --- \| --- \| --- \| --- \| --- \| --- \| --- \| --- \| --- \| --- \| \|  \|  \| - \|  \|  \|  \| - \|  \|  \|  \|  \| | |
| Young person’s sex | | Male  Female |
| Young person’s age  *If <5 or >17 do not proceed* | | \|  \|  \| \| --- \| --- \| \|  \|  \| |
| Has the young person been previously enrolled and randomised in the STADIA trial?  *If yes, do not proceed* | | Yes  No |
| Does the referral mention any of the following Covid-19 related words/phrases?  *Tick all that apply.*  Covid-19 / Covid  Coronavirus  Lockdown  School closure / exams cancelled | |  |
| Does the referral mention emotional difficulties*?  *If no, do not proceed* | | Yes  No |
| Is this an emergency or urgent referral (according to local CAMHS triage / SPA team risk assessment)?  *If yes, do not proceed* | | Yes  No |
| Does the young person have severe learning disability (e.g., the referral mentions this or that they attend a special school for children with severe learning difficulties)?  *If ‘yes’ do not proceed*  *If not known, confirm during telephone eligibility check at enrolment* | | Yes  No  Not known |
| If the young person is <16:  Does the referral information include contact details for a named parent/carer?  *If ‘no’ await parent/carer contact details before proceeding* | | Yes  No  N/A |
| If the young person is <16:  Is the named parent/carer a local authority representative designated to care for the child/young person?  *If ‘yes’ do not proceed*  *If not known, confirm during telephone eligibility check at enrolment* | | Yes  No  Not known  N/A |
| If the young person is aged 16 or 17:  Whose contact details are given on the referral form?  *If young person contact details are provided, they should be contacted in the first instance* | | Young person  Parent/carer  Both  N/A |

| **EMOTIONAL DIFFICULTIES** | |
| --- | --- |
| **Emotional difficulties may be indicated by the use of any of the following key words or phrases.*  *Tick all that apply. If ‘other’ record details and seek advice from the PI or NCTU before proceeding.* | |
| None |  |
| Agitated / agitation |  |
| Anger |  |
| Anxiety / anxious / generalised anxiety |  |
| Avoids things/people/places |  |
| Can’t leave the house |  |
| Completing rituals / asking parents to carry out rituals |  |
| Compulsions |  |
| Depressed / depression / low / low mood / sad |  |
| Difficulties sleeping |  |
| Distress |  |
| Fears and worries / fears relating to safety (germs, fire) |  |
| Feeling low |  |
| Feels flat / empty / blank |  |
| Feels hopeless |  |
| Feels worthless / stupid |  |
| Flashbacks |  |
| Hypervigilance |  |
| Irritable |  |
| Low motivation |  |
| Low self-esteem / Hates self |  |
| Mood swings / moody |  |
| Negative thoughts |  |
| Nightmares (if trauma also present) |  |
| No (or loss of) energy |  |
| No (or loss of) interest in things / gave up… / lack of wanting to do things |  |
| Not going to school / unable to go to school |  |
| Not sleeping / poor sleep |  |
| Obsessions |  |
| OCD |  |
| Phobia |  |
| Panic / panic attacks |  |
| PTSD |  |
| Self-harm / DSH / Cutting |  |
| Suicidal |  |
| Suicidal thoughts / thoughts of ending life / thinks about killing self |  |
| Tearful |  |
| Thoughts of death |  |
| Tiredness / fatigue |  |
| Touching objects |  |
| Trauma |  |
| Weepy |  |
| Withdrawal / withdrawn |  |
| Worried / worrying (incl. worries/concerns about their appearance |  |
| Other (please specify) |  |

| *FOR ALL REFERRALS SCREENED, ENTER SUMMARY DATA ON THE SCREENING & ENROLMENT LOG.*  *IF THE YOUNG PERSON APPEARS TO BE ELIGIBLE PROCEED TO THE INVITATION TELEPHONE CALL (CALL 1) AND ENTER DETAILS ON THE TRIAL DATABASE.* |
| --- |

| **SIGN-OFF STATEMENT** | |
| --- | --- |
| *Completed by the researcher conducting the referral screening.* | |
| To the best of my knowledge, I confirm that I have made every reasonable effort to ensure that ALL of the data in this Case Record Form is a true, accurate and complete report. | |
| Print Name |  |
| Signature |  |
| Date | \|  \|  \|  \|  \|  \|  \|  \|  \|  \|  \|  \| \| --- \| --- \| --- \| --- \| --- \| --- \| --- \| --- \| --- \| --- \| --- \| \|  \|  \| - \|  \|  \|  \| - \|  \|  \|  \|  \| |

Changes were made to the original screening form (v1.0 30-May-2019) during the covid-19 pandemic. This was superseded by v1.1 30-Apr-2020.

Figure 1.2: Participant flow


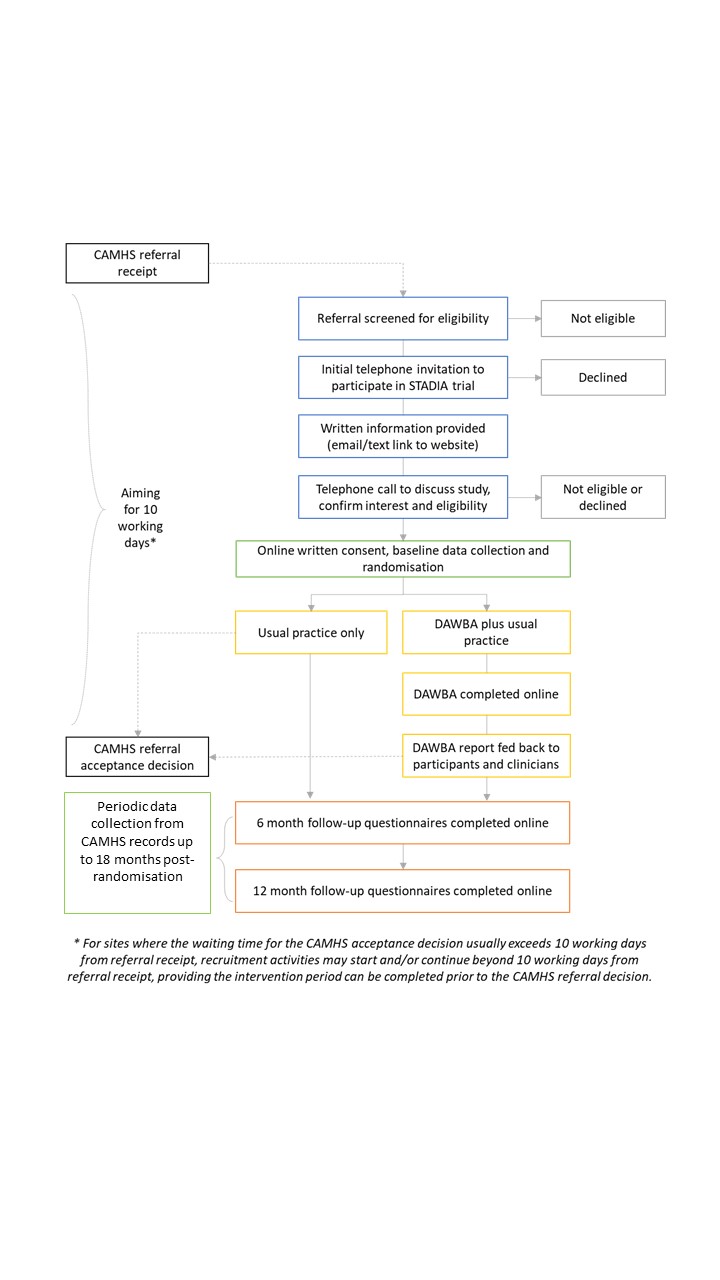


Table 1.3: Consent and participation

| **Age of CYP referred to CAMHS:** | **CYP aged <11** | **CYP aged 11-15** | | **CYP aged 16-17** | |
| --- | --- | --- | --- | --- | --- |
| **Initial contact with:** | Parent/carer | | | Depends on contact details provided with the CAMHS referral* | |
| **Consent provided by:** | Parent/carer | Parent/carer | Parent/carer | CYP AND parent/carer (optional) | CYP |
| **Assent provided by:** | None | CYP (optional) | None | None | None |
| **Participant(s):** | Parent/carer only | CYP and parent/carer dyad | Parent/carer only | CYP and parent/carer dyad | CYP only |
| **Primary participant:**** | Parent/carer | Parent/carer | Parent/carer | CYP | CYP |
| **Secondary participant:** | None | CYP | Non | Parent/carer | None |
| **DAWBA completed by:** | Parent/carer | Parent/carer AND  CYP | Parent/carer | CYP AND parent/carer | CYP |
| **Research questionnaires completed by:** | Parent/carer report on CYP  Parent/carer self-report | Parent/carer report on CYP  Parent/carer self-report  CYP self-report | Parent/carer report on CYP  Parent/carer self-report | CYP self-report Parent/carer report on CYP  Parent/carer self-report | CYP self-report |
| For all CYP aged <16 the initial contact about the study will be with the parent/carer. The involvement of CYP aged 11-15 will be at the discretion of the parent/carer.  * For CYP aged 16-17 if the CYP’s contact details are provided on the CAMHS referral the first contact about the study will be with the CYP who can choose to nominate a parent/carer to participate in the trial alongside them or participate alone. If the parent/carer’s contact details only are available the first contact will be with the parent/carer and the parent/carer will be asked whether the CYP can also be contacted but may choose to refuse this. The parent/carer will not be able to participate in the STADIA trial without the involvement or consent of the CYP.  ** The primary participant is the person who must provide consent as a minimum requirement in order for randomisation to take place. Assent (of CYP aged 11-15) and parental consent (for CYP aged 16 and 17) may also be sought but is not mandatory and therefore will not be required prior to randomisation. | | | | | |

Table 1.4: Outcome definition and adjudication plan


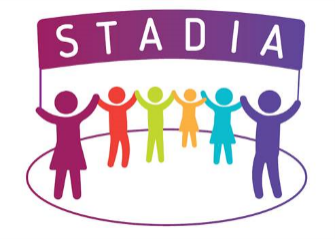


**STAndardised DIagnostic Assessment for children and adolescents with emotional difficulties (STADIA):**

**A multi-centre randomised controlled trial**

**OUTCOME DEFINITION & ADJUDICATION PLAN**

**Final 1.0 – 25 February 2020**

**EMOTIONAL DISORDER DIAGNOSES RECORDED IN THE 12 MONTHS POST-RANDOMISATION**

| **CONSTITUTES A CLINICAL DIAGNOSIS** | **REFER FOR ADJUDICATION** | **DOES NOT CONSTITUTE A CLINICAL DIAGNOSIS** |
| --- | --- | --- |
| - The presence of an eligible diagnosis within the diagnosis tab of the clinical record. - The presence of an eligible diagnosis in the clinical record preceded by the heading ‘diagnosis’. - The presence of an eligible diagnosis in the clinical record preceded by a heading such as ‘current difficulties’ or ‘presenting problems’, except where this has been documented in the write up of the first appointment or in reference to the information received at referral (as this may simply reflect a pre-existing or referrer-made diagnosis). - A clear confirmatory statement including use of an eligible diagnosis, for example:   *Meets the diagnostic criteria for…*  *Presentation is explained by a diagnosis of…* | - The presence of similar diagnostic terms within the diagnosis tab of the clinical record. - The presence of an eligible diagnosis preceded by a heading such as ‘current difficulties’ or ‘presenting problems’, documented in the write up of the first appointment or in reference to the information received at referral. - The presence of similar diagnostic terms preceded by a heading such as ‘diagnosis’, ‘current difficulties’ or ‘presenting problems’. - Reference to an eligible diagnosis or similar diagnostic terms, but where the context does not provide a clear confirmatory statement, for example:   *?...*  *Possible…*  *Assessed for…*  *…-type symptoms / behaviour*  *…-like symptoms / behaviour*  *Symptoms of…*  *History of…* | - No reference to an eligible diagnosis or similar diagnostic terms. - A clear statement about the absence of an eligible diagnosis or similar diagnostic terms, for example:   *No evidence of…*  *…not meeting criteria for disorder* |
| *Data collection and entry: instructions for researchers* | | |
| *Use the checklist of eligible emotional disorder diagnoses.* | *Document these as other emotional disorders.* | *Check ‘none of the above’ in the checklist of emotional disorder diagnoses and answer ‘no’ to ‘other emotional disorder diagnoses’.* |

*Note: For definition of underlined terms see the Glossary below.*

**NO EMOTIONAL DISORDER**

If there are no emotional disorder diagnoses documented in the CAMHS notes in 12 months post-randomisation, researchers will select one of the following options:

1. A clinician has documented the absence of emotional disorder.
2. Uncertainty about the presence of an emotional disorder is documented in the notes (for example, reflecting ongoing assessment / investigation).
3. There is no diagnostic information relating to emotional disorders documented in the CAMHS record.

| **A clinician has documented the absence of emotional disorder.** | **Uncertainty about the presence of an emotional disorder is documented in the notes (for example, reflecting ongoing assessment / investigation).** | **There is no diagnostic information relating to emotional disorders documented in the CAMHS record.** |
| --- | --- | --- |
| - Nothing in the clinical record is assessed to constitute a documented clinical diagnosis, AND - There is a clear statement about the absence of one or more of the eligible diagnoses or similar diagnostic terms, for example:   *No evidence of…*  *…not meeting criteria for disorder* | - Nothing in the clinical record is assessed to constitute a documented clinical diagnosis, AND - Reference to an eligible diagnosis or similar diagnostic terms, but where the context does not provide a clear confirmatory statement, for example:   *?...*  *Possible…*  *Assessed for…*  *…-type symptoms / behaviour*  *…-like symptoms / behaviour*  *Symptoms of…*  *History of…* | - Nothing in the clinical record is assessed to constitute a documented clinical diagnosis, AND - There is no reference to an eligible diagnosis or similar diagnostic terms.* - If emotional difficulties are identified they are described only by reference to the presenting symptoms with no attempt made to link these to an eligible diagnosis, for example:   *Presenting issue - Mood swings*  *Describing examples of ruminating thoughts.*  * Note that this includes children/young people who have not been seen by CAMHS in the 12-months post-randomisation. |
| *Document these as absence of emotional disorder.* | **REFER FOR ADJUDICATION** | **MAY REQUIRE ADJUDICATION** |

| **EMOTIONAL DISORDER DIAGNOSIS ADJUDICATION OUTCOME** | |
| --- | --- |
| The Adjudication Committee will first consider whether the record:   1. Constitutes a clinical diagnosis 2. Does not constitute a clinical diagnosis | If (1) then the Adjudication Committee will determine which of the eligible emotional disorder diagnoses apply. |
|  | If (2) then the Adjudication Committee will determine whether the record constitutes:   1. Absence of emotional disorder 2. Uncertainty about the presence of emotional disorder 3. No diagnostic information |

**TREATMENTS / INTERVENTIONS GIVEN**

| **CONSTITUTES A TREATMENT / INTERVENTION** | **REFER FOR ADJUDICATION** |
| --- | --- |
| - The presence of an eligible treatment / intervention documented within the clinical record. | - Documented intervention by CAMHS where the description does not include an eligible treatment / intervention. |
| *Data collection and entry: instructions for researchers* | |
| *Use the checklist of eligible treatments / interventions.* | *Document these as other treatments / interventions.* |

| **TREATMENTS / INTERVENTIONS ADJUDICATION OUTCOME** | |
| --- | --- |
| The Adjudication Committee will first consider whether the record:   1. Constitutes a treatment / intervention 2. Does not constitute a treatment / intervention | If (1) then the Adjudication Committee will determine whether the record should be categorised:   1. As an existing treatment / intervention 2. As an ‘other’ treatment / intervention |

| **GLOSSARY** | |
| --- | --- |
| Eligible diagnosis | One of the pre-specified diagnoses listed on the data collection form. These should be considered present only when the exact phrase and/or corresponding ICD/DSM code is documented. |
| Similar diagnostic terms | Words or phrases which are similar to the eligible diagnoses, but without use of the exact wording or corresponding ICD/DSM code (e.g., separation anxiety WITHOUT use of the term disorder) or where the exact words are used alongside additional phrases (e.g., OCD-type behaviour or OCD-like symptoms). |
| Eligible treatment / intervention | One of the pre-specified treatments / interventions listed on the data collection form. |
| Adjudication Committee | The Adjudication Committee will comprise the clinician members of the Trial Management Group. A minimum of two clinicians will review terms referred for adjudication, with a third consulted if a consensus is not reached.  The Adjudication Committee will be blinded to treatment allocation for the purposes of adjudication. |

Table 1.5: Summary of assessments

| **Time-point** | **Maximum 10 working days from referral receipt^1^** | | | | | **6 months post-randomisation** | **12 months post-randomisation** | **18 months post-randomisation** |
| --- | --- | --- | --- | --- | --- | --- | --- | --- |
| **Activity** | **Screening and invitation** | **Eligibility and enrolment** | **Consent and baseline** | **Randomisation** | **Intervention**  DAWBA in addition to assessment-as-usual  Or  Assessment-as-usual | **Follow-Up** | |  |
| Initial eligibility screen of referral information | X |  |  |  |  |  |  |  |
| Telephone invitation to participate | X |  |  |  |  |  |  |  |
| Verbal agreement to participate |  | X |  |  |  |  |  |  |
| Confirm eligibility |  | X |  |  |  |  |  |  |
| Obtain enrolment data |  | X |  |  |  |  |  |  |
| Participant enrolment |  | X |  |  |  |  |  |  |
| Written informed consent/assent (online) |  |  | X |  |  |  |  |  |
| Baseline demographics (parent/carer and CYP aged 16 & 17) |  |  | X |  |  |  |  |  |
| Mood and Feelings Questionnaire (MFQ) |  |  | X |  |  | X | X |  |
| Revised Child’s Anxiety Depression Scale (RCADS) |  |  | X |  |  | X | X |  |
| Strengths and Difficulties Questionnaire (SDQ)^2^ |  |  | X |  |  | X | X |  |
| Child Revised Impact of Events Scale (CRIES-8)(Perrin, Meiser-Stedman, & Smith, 2005)^3^ |  |  | X |  |  | X | X |  |
| CYP self-report self-harm measure |  |  | X |  |  | X | X |  |
| Patient Health Questionnaire (PHQ-9) - parent/carer only |  |  | X |  |  | X | X |  |
| Generalised Anxiety Disorder Assessment (GAD-7) - parent/carer only |  |  | X |  |  | X | X |  |
| Child Health Utility 9D (CHU9D) |  |  | X |  |  | X | X |  |
| EuroQol-5D youth (EQ-5D-Y) |  |  | X |  |  | X | X |  |
| EuroQol-5D five level (EQ-5D-5L) |  |  | X |  |  | X | X |  |
| Resource Use Questionnaire - parent/carer and CYP aged 16 & 17 |  |  | X |  |  | X | X |  |
| Data collection from records^4^ |  |  | X |  |  | X | X | X |
| **^1^** For sites where the waiting time for the CAMHS acceptance decision usually exceeds 10 working days from referral receipt, recruitment activities may start and/or continue beyond 10 working days from referral receipt, providing the intervention period can be completed prior to the CAMHS referral decision.  ^2^ For participants in the intervention arm, the baseline SDQ will be collected as part of the DAWBA, completed post-randomisation.  ^3^ Additional data collection undertaken to explore post-traumatic stress disorder symptoms in CYP during the Covid-19 pandemic  ^4^ Data collection from records completed periodically throughout the 12 and 18 month follow-up period. | | | | | | | | |

Figure 1.3: DAWBA report template

**DAWBA Report**

The DAWBA collects information about a range of common emotional and behavioural difficulties, and uses this information to produce a report to highlight the level of difficulties.

**How to understand the ratings**

These ratings compare your responses with the responses from large numbers of other parents and young people across the UK. Many parents and young people find this sort of comparison helpful, but it is just a guide and not the same as a face-to-face assessment with a specialist.

To make it easier to read, we have grouped the ratings into four categories. Each category is different. This shows how your [child’s] *(delete as appropriate)* difficulties compare with other children / young people:

| 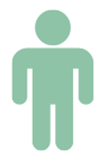 | **Close to average**  In the general population most children/ young people (roughly 80 out of 100) are in the “close to average” category. | **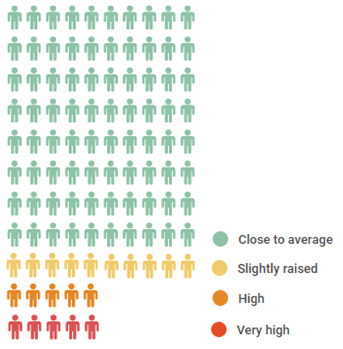** |
| --- | --- | --- |
| 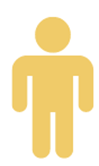 | **Slightly raised**  If the ratings are in the “slightly raised” category this means the difficulties are slightly higher than average. Roughly 10 out of 100 children / young people are in this category. |  |
| 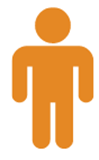 | **High**  Around 5 in 100 children / young people score in the “high” category. This means that the difficulties are more severe than average. |  |
| 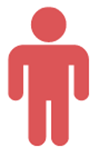 | **Very high**  Around 5 in 100 children score in the “very high” category. This means that the difficulties appear to be more severe than we find in 95 out of every 100 children / young people. |  |

The rating is only a rough guide. As high ratings can be a "false alarm", please use your own judgement. Not all difficulties need treating. Some difficulties get better by themselves, particularly if they are mild or if they have only been there for a short time.

Most strengths and difficulties lie on a scale. There will be children / young people at each end of the scale but most children / young people will fall somewhere in between.

**Your [child’s]** *(delete as appropriate)* **ratings:**

- **Close to** **average / Slightly raised / High / Very high** for worrying a lot about different things (general fears and worries)
- **Close to** **average / Slightly raised / High / Very high** for worries about separation from key "attachment figures" such as parents (separation anxiety)
- **Close to** **average / Slightly raised / High / Very high** for specific fears (specific phobia)
- **Close to** **average / Slightly raised / High / Very high** for social fears (social anxiety)
- **Close to** **average / Slightly raised / High / Very high** for panic attacks
- **Close to** **average / Slightly raised / High / Very high** for fears of crowds, public places, open spaces etc (agoraphobia)
- **Close to** **average / Slightly raised / High / Very high** for stress linked to particularly frightening events (post-traumatic stress)
- **Close to** **average / Slightly raised / High / Very high** for obsessions or compulsions
- **Close to** **average / Slightly raised / High / Very high** for depression or loss of interest
- **Close to** **average / Slightly raised / High / Very high** for disruptive and uncooperative behaviours (troublesome behaviour)
- **Close to** **average / Slightly raised / High / Very high** for antisocial or aggressive behaviours that can get people into serious trouble (troublesome behaviour)

Table 1.6: Eligible emotional disorder diagnoses

*Added following agreement from adjudication committee.

| Anxiety disorder |
| --- |
| Separation anxiety disorder |
| Specific phobia (any) |
| Social phobia or Social anxiety disorder |
| Agoraphobia |
| Panic disorder (DSM5 additionally has Panic Attack with a specifier) |
| Phobic anxiety disorder (unspecified) |
| Selective mutism |
| Generalized anxiety disorder |
| Obsessive-compulsive and related disorders |
| Body dysmorphic disorder |
| Acute stress reaction |
| Acute Stress Disorder |
| Post-traumatic stress disorder |
| Adjustment Disorder |
| Other anxiety disorder |
| Mixed anxiety and depressive disorder |
| Depression |
| Depressive episode (any / mild / moderate / severe) |
| Depressive disorder |
| Recurrent depressive disorder (any / mild / moderate / severe) |
| Major Depressive disorder |
| Persistent Depressive disorder |
| Other depressive episode |
| Persistent mood (affective) disorder  (including cyclothymic disorder / dysthymic disorder) |
| Other / Unspecified mood (affective) disorder |
| Bipolar disorder |
| Bipolar affective disorder |
| Manic episode |
| Childhood emotional disorder unspecified (F93.9) |
| Trichotillomania* |

Table 1.7: Secondary Outcome Measures

| **Outcome** | **Measurement** | **Definition** | **Location** |
| --- | --- | --- | --- |
| Clinician-made diagnosis decision about the presence of an emotional disorder within 18 months of randomisation | Collected from records | The diagnosis must be documented in the clinical record within 18 months of randomisation by a mental health services clinician in an NHS-delivered or NHS-commissioned service. Any eligible diagnosis made within 18 months included. | Table 2.21 in appendix |
| Acceptance of index referral | Collected from records | Whether the index referral (i.e., the referral made to CAMHS at the point of recruitment to the STADIA trial) was accepted or declined.  Acceptance is defined as being offered an appointment within CAMHS, whether or not the initial appointment was attended or subsequent appointments were offered/attended. | Table 2.22 in appendix |
| Acceptance of any referral within 12 and 18 months | Collected from records | Whether the index referral or any subsequent referral to CAMHS (if made) was accepted or not. Acceptance as defined above for index referral. Collected within 12 and 18 months of randomisation. | Tables 2.22 and 2.23 in appendix |
| Discharge from CAMHS within 12 and 18 months | Collected from records | Whether the child/young person was discharged from CAMHS (following acceptance of the index referral) during the 12 and 18-months post-randomisation. | Tables 2.22 and 2.23 in appendix |
| Re-referral to CAMHS within 12 and 18 months | Collected from records | Whether the child/young person was re-referred to CAMHS (for those whose index referral was turned down by CAMHS or those whose index referral was accepted but were subsequently discharged) during the 12 and 18-months post-randomisation. | Tables 2.22 and 2.23 in appendix |
| Confirmed diagnosis decision | Collected from records | Diagnosis of an emotional disorder or confirmed absence of an emotional disorder coded as ‘yes’ vs. uncertainty about the presence of an emotional disorder coded as ‘no’. Diagnosis as defined for primary outcome, collected within 12 and 18 months of randomisation. | Tables 2.18 and 2.21 in appendix |
| Time from randomisation to diagnosis of emotional disorder | Collected from records | Date of diagnosis will be the first documented eligible diagnosis. Diagnosis as defined above for primary outcome collected within 12 and 18 months of randomisation | Tables 2.18 and 2.21 in appendix |
| Diagnoses made over the 12 and 18 period from randomisation | Collected from records | The diagnosis must be documented in the clinical record within 12 and 18 months of randomisation by a mental health services clinician in an NHS-delivered or NHS-commissioned service. All diagnoses made within 12 and 18 months will be included. | Table 2.45 in appendix |
| Treatment offered for diagnosed emotional disorder | Collected from records | Whether treatment was offered for a diagnosed emotional disorder, as defined for primary outcome above, collected within 12 and 18 months of randomisation. | Tables 2.24 and 2.25 in appendix |
| Treatments / interventions given | Collected from records | All treatments/interventions offered by CAMHS for any reason within 12 and 18 months of randomisation, whether or not there is a documented diagnosis will be included. | Tables 2.47-2.52 in appendix |
| Time from randomisation to the decision to offer treatment for a diagnosed emotional disorder | Collected from records | Date of decision will be the first date that the decision to offer treatment for a diagnosed emotional disorder is documented in the clinical note, collected within 12 and 18 months of randomisation. | Tables 2.24 and 2.25 in appendix |
| Time from randomisation to start of first treatment for a diagnosed emotional disorder | Collected from records | Date of treatment will be the first date that any treatment offered for a diagnosed emotional disorder is started. Treatment and diagnosed emotional disorder as defined as above, collected within 12 and 18 months of randomisation. | Tables 2.24 and 2.25 in appendix |
| Time from randomisation to the decision to offer any treatment | Collected from records | Date of decision will be the first date that the decision to offer any treatment is documented in the clinical notes, collected within 12 and 18 months of randomisation. | Tables 2.24 and 2.25 in appendix |
| Time from randomisation to start of any treatment | Collected from records | Date of treatment will be the first date that any treatment offered is started. Treatment as defined as above, collected within 12 and 18 months of randomisation. | Tables 2.24 and 2.25 in appendix |
| Participant-reported diagnoses | Participant self-report | Participants will be asked to report whether or not they received a diagnosis of the child/young person’s difficulties from CAMHS in the 12 months post-randomisation and if so, what diagnosis was given and by whom. | Table 2.46 in appendix |
| Depression symptoms in the child/young person | Mood and Feelings Questionnaire (MFQ) | Mood and Feelings Questionnaire (MFQ) (Angold, Costello, Messer, & Pickles, 1995) is a valid and reliable measure of depression in CYP.(Daviss et al., 2006; Wood, Kroll, Moore, & Harrington, 1995) 33-items are answered on a 3-point scale. Scores range from 0 to 66 with higher scores indicating more severe depressive symptoms. A score of 27 or higher may be indicative of depression. MFQ collected at baseline, 6 and 12 months post-randomisation. | Table 2.26 in appendix |
| Anxiety symptoms in the child/young person | Revised CYP’s Anxiety Depression Scale (RCADS) | Revised CYP’s Anxiety and Depression Scale (RCADS)(Chorpita, Yim, Moffitt, Umemoto, & Francis, 2000)  RCADS is a 47-item questionnaire that measures the reported frequency of various symptoms of anxiety and low mood. Each item is rated on a 4-point scale. An overall anxiety and low mood score is generated, with separate sub-scale scores for separation anxiety, social phobia, generalised anxiety, panic, obsessive compulsive disorder and major depression.  Total anxiety and depression scores range from 0 to 141.    We will record scores for each of the 6 sub-scales. For analysis metric, we will use the total anxiety score. RCADS collected at baseline, 6 and 12 months post-randomisation. | Tables 2.26 and 2.27 in appendix |
| Comorbid oppositional defiant / conduct disorder in the child/young person | Strengths and Difficulties Questionnaire (SDQ) | Strengths & Difficulties Questionnaire (SDQ):(Goodman, 1999) A 25-item emotional and behavioural screening questionnaire for CYP.  Each item is rated on a 3-point scale.  SDQ comprises 5 sub-scales and an impact supplement. The impact supplement asks effect of difficulties on homelife, friendships, education and leisure activities.  Scores on the ‘conduct problems’ subscale will be used in the analysis of this outcome.  Sub-scale scores range from 0 to 10. SDQ collected at baseline, 6 and 12 months post-randomisation. | Tables 2.26 and 2.27 in appendix |
| Functional Impairment in the child/young person | Strengths and Difficulties Questionnaire (SDQ) | Impact supplement scores will be used to determine functional impairment. Impact scores range from 0 to 10. Collected at baseline, 6 and 12-months post-randomisation. | Tables 2.26 and 2.27 in appendix |
| Self-harm thoughts in the child/young person | CYP self-report self-harm measure | CYP will be asked to report the frequency of thoughts of self-harm.  Frequency of thoughts of self-harm are rated over the last 6 months in the following categories and scored accordingly:  Not at all (0)  Once or twice (1)  Three or more times (2)  Collected at baseline, 6 months and 12-months post-randomisation. | Table 2.35 in appendix |
| Self-harm behaviours in the child/young person | CYP self-report self-harm measure | CYP will be asked to report frequency of instances of self-harm behaviour.  Frequency of self-harm behaviour are rated over the last 6 months in the following categories and scored accordingly:  Not at all (0)  Once (1)  Two or more times (2)  Collected at baseline, 6 months and 12-months post-randomisation. | Table 2.35 in appendix |
| Depression symptoms in the parent/carer | Patient Health Questionnaire (PHQ-9) | PHQ-9:(Kroenke, Spitzer, & Williams, 2001) PHQ-9 is frequently used as a screening tool for depression in general populations. Each of the nine DSM-IV depression criteria are scored as "0" (not at all) to "3" (nearly every day) depending on the frequency with which they were experienced over the last 2 weeks. Total scores range from 0 to 27 with higher scores indicating increased severity of depression, collected at baseline, 6 and 12-months post-randomisation. | Table 2.28 in appendix |
| Anxiety symptoms in the parent/carer | Generalised Anxiety Disorder Assessment (GAD-7) | GAD-7:(Spitzer, Kroenke, Williams, & Löwe, 2006) is a measure of the severity of anxiety in general populations. 7 items are rated according to the frequency with which they have been experienced over the past 2 weeks Total scores range from 0 to 21 with higher scores indicating more severe anxiety. Collected at baseline, 6 and 12-months post-randomisation. | Table 2.28 in appendix |
| Time out of education, employment or training for the child/young person | Resource use questionnaire | Days missed from education, employment or training (as applicable) for the CYP due to emotional difficulties. Collected at baseline, 6 and 12-months post-randomisation. | Table 2.29 in appendix |
| *Safety Outcomes* |  |  |  |
| A significant deterioration in depression for the child/young person | MFQ (as above) | A score indicative of depression (27 or above) on the Mood and Feelings Questionnaire (MFQ) completed at follow-up, where this represents a deterioration from baseline of 5 points or more. | Tables 2.35 and 2.36 in appendix |
| A significant deterioration in depression for the parent/carer | PHQ-9 (as above) | A score indicative of depression (15 or above) on the Patient Health Questionnaire (PHQ-9) completed at follow-up, where this represents a deterioration from baseline of 5 points or more. | Table 2.38 in appendix |
| Frequency of self-harm | Self-harm measure (as above) | Frequency of self-harm behaviour are rated over the last 6 months in the following categories and scored accordingly:  Not at all (0)  Once (1)  Two or more times (2)  Collected at baseline, 6 months and 12-months post-randomisation. | Table 2.35 in appendix |
| Hospital admissions | Participant self reported | The number of children/young people admitted to hospital (either physical or mental health-related) due to emotional difficulties as reported in the resource use questionnaire completed at 6- and 12 months post-randomisation. | Table 2.37 in appendix |
| A&E attendances | Resource use questionnaire | The number of children/young people attending A&E (either physical or mental health-related) due to emotional difficulties as reported in the resource use questionnaire completed at 6- and 12 months post-randomisation. | Table 2.37 in appendix |
| Deaths | Data collected from records | The number of deaths of participating children/young people from randomisation until 18-month follow-up. | Tables 2.39 and 2.40 in appendix |
| *Additional data collection* |  |  |  |
| Post-traumatic stress disorder symptoms in the child/young person | The Children’s Revised Impact of Event Scale (CRIES-8) | Scores range from 0 to 40 with higher scores indicating more severe symptoms.  There are two subscales: Intrusion and avoidance. Sub-scale scores range from 0 to 20.  There are 8 items that are scored on a four point scale. | Table 2.33 and 2.34 in appendix |
| *Health economic outcome measures* |  |  |  |
| Health related quality of life in the CYP | Child Health Utility 9D (CHU9D) and EuroQol Quality of Life Questionnaire 5 Domains for Young People (EQ-5D-Y) | CHU9D (Stevens, 2009) consists of nine individual items with five levels of response per question (scored 1-5), that assess the CYP functioning “today”. The following domains are included; worry, sadness, pain, tiredness, annoyance, school, sleep, daily routine and activities.  EuroQol-5D youth descriptive system (Wille et al., 2010) comprises 5 domains; mobility, looking after myself, doing usual activities, having pain or discomfort and feeling worried, sad or unhappy, values of 1, 2 or 3 are assigned to each response. The EuroQol Visual Analogue Scale (EQ-VAS) asks recipients to self-assess their health state ‘today’ from 0 (worst imaginable health) to 100 (best imaginable health), representing individual preferences.  These measures will be self-reported by CYP aged 11+, with proxy versions also completed by the parent/carer for CYP <16. Both collected at baseline, 6 and 12-months post-randomisation. | Table 3.5 in appendix |
| Health-related quality of life in the parent/carer | EuroQol Quality of Life Questionnaire 5 Domains, 5 Levels (EQ-5D-5L) | The EuroQol 5-dimension multi attribute utility instrument (Herdman et al., 2011)comprises 5 domains; mobility, self-care, usual activities, pain/discomfort and anxiety/depression. Each domain is scored between 1 and 5. This descriptive profile, in combination with a valuation set, produces a single index for health status representing societal preferences. The index score ranges from -0.59 to 1, with 0 representing death, 1 of-perfect health, and <0 of health states worse than death. The EQ-VAS is again included within the EQ-5D instrument Collected at baseline, 6 and 12-months post-randomisation. | Table 3.5  in appendix |

# STADIA Trial Results

## Summary of Recruitment and Analysis Sets

### CONSORT diagram

The full participant flow diagram (CONSORT) is shown in Figure 2.1.

**Number of eligible referrals screened for STADIA trial**

**(n = 5023)**

**Excluded (n = 3798)**

- Unable to contact (n = 1100)

- Referral details not obtained (n=158)

- Not sent written information (n = 507)

- Did not proceed after being sent written information (n = 1525)

- Did not proceed after being sent link to electronic consent form (n = 393)

- Did not proceed following consent
(n = 115)

**Allocated to Intervention Group (n = 615)**

**Primary participant**

Parent/carer n = 536

Young person n = 79

- DAWBA fully or partially completed by either young person or parent/carer (n = 494)

**Allocated to Control Group (n = 610)**

**Primary participant**

Parent/carer n = 530

Young person n = 80

**Number randomised**

**(n = 1225 children/young people)**

(n = 1121 parents/carers)

**6 month follow-up questionnaire returned by**

Primary participant (n = 480)

Primary/secondary participant (n = 483)

**12 month follow-up questionnaire returned by** Primary participant (n = 477)

Primary/secondary participant (n = 478)

**6 month follow-up questionnaire returned by**

Primary participant (n = 493)

Primary/secondary participant (n = 495)

**12 month follow-up questionnaire returned by**

Primary participant (n = 481)

Primary/secondary participant (n = 482)

**Primary outcome not available (n =1)**

*Withdrawn consent (n = 1)*

**Included in 18-month secondary analysis
(n = 609)**

**Data collected from records at 12 months
(n = 615)**

**Primary outcome not available (n = 5)**

*Withdrawn consent (n = 5)*

**Data collected from records at 12 months
(n = 610)**

**Primary outcome not available (n = 6)**

*Withdrawn consent (n = 6)*

**Primary outcome not available (n = 4)**

*Withdrawn consent (n = 4)*

**Included in primary analysis (n = 609)**

**Data collected from records at 18 months
(n = 610)**

**Included in primary analysis (n = 610)**

**Data collected from records at 18 months
(n = 615)**

**Included in 18-month secondary analysis
(n = 606)**

Figure 2.1: Participant flow diagram (CONSORT)

### Analysis set

**Table 2.1: Analysis set definitions and numbers in each group**

| **Analysis set** | **Definition** | **Intervention Group** | **Control Group** | **Total** |
| --- | --- | --- | --- | --- |
| **Randomised** | | | | |
| **Total randomised** | All participants randomised | n = 615 | n = 610 | n = 1225 |
| **Child self-report** | Children aged 11-15 whose parents consent to child participating and children aged 16-17 | n = 252 | n = 250 | n = 502 |
| **Parent/carer report** | Children aged 5-15 and children aged 16-17 with a parent/carer taking part | n = 560 | n = 561 | n = 1121 |
| **Analysis set** | | | | |
| **Primary** | Participants analysed in the group to which they were randomly allocated with no imputation of missing data. | n = 610 | n = 609 | n = 1219 |

### Trial recruitment

Table 2.2: Trial recruitment by intervention arm and participating site

| **Site** | **Intervention Group** | **Control Group** | **Total** |
| --- | --- | --- | --- |
| Nottinghamshire Healthcare NHS Foundation Trust | 126 | 126 | 252 |
| Berkshire Healthcare NHS Foundation Trust | 101 | 103 | 204 |
| Cambridgeshire & Peterborough NHS Foundation Trust | 160 | 159 | 319 |
| Central & North West London NHS Foundation Trust | 80 | 84 | 164 |
| Pennine Care NHS Foundation Trust | 105 | 109 | 214 |
| Gloucestershire Health and Social Care NHS Foundation Trust | 22 | 19 | 41 |
| Surrey and Borders NHS Foundation Trust | 18 | 8 | 26 |
| Rotherham, Doncaster and South Humber NHS Foundation Trust | 3 | 2 | 5 |
| **All sites** | **615** | **610** | **1225** |

All data are N (%) unless otherwise indicated.

## Baseline characteristic

### Baseline characteristics of the child/young person

**Table 2.3: Child baseline characteristics data (minimisation factors highlighted)**

| **Characteristic** | **Intervention Group**  **(n = 615)** | **Control Group**  **(n = 610)** | **Total randomised (n = 1225)** |
| --- | --- | --- | --- |
| **Age at randomisation (years)** |  |  |  |
| Mean [sd] | 11.9 [3.1] | 12 [3.1] | 12 [3.1] |
| Median [25th, 75th centile] | 12 [10, 14] | 13 [9, 15] | 12 [9, 15] |
| Min, max | 5, 17 | 5, 17 | 5, 17 |
| 5-10 | 212 (34%) | 210 (34%) | 422 (34%) |
| 11-15 | 324 (53%) | 320 (52%) | 644 (53%) |
| 16-17 | 79 (13%) | 80 (13%) | 159 (13%) |
| **Sex*** |  |  |  |
| Male | 257 (42%) | 254 (42%) | 511 (42%) |
| Female | 358 (58%) | 356 (58%) | 714 (58%) |
| **Gender**** |  |  |  |
| Male | 256 (42%) | 252 (42%) | 508 (42%) |
| Female | 344 (57%) | 339 (57%) | 683 (57%) |
| Other | 6 (1%) | 7 (1%) | 13 (1%) |
| Missing | 9 | 12 | 21 |
| **Ethnicity** |  |  |  |
| White | 524 (86%) | 516 (86%) | 1040 (86%) |
| Indian | 10 (2%) | 12 (2%) | 22 (2%) |
| Pakistani | 9 (1%) | 6 (1%) | 15 (1%) |
| Bangladeshi | 1 (<1%) | 0 (0%) | 1 (<1%) |
| Black Caribbean | 6 (1%) | 6 (1%) | 12 (1%) |
| Black African | 0 (0%) | 2 (<1%) | 2 (<1%) |
| Black (other) | 1 (<1%) | 0 (0%) | 1 (<1%) |
| Chinese | 2 (<1%) | 0 (0%) | 2 (<1%) |
| Other Asian (Non-Chinese) | 6 (1%) | 6 (1%) | 12 (1%) |
| Dual/mixed heritage | 41 (7%) | 45 (8%) | 86 (7%) |
| Other | 7 (1%) | 5 (1%) | 12 (1%) |
| Missing | 8 | 12 | 20 |
| **In education (e.g. school or college)** |  |  |  |
| No | 13 (2%) | 23 (4%) | 36 (3%) |
| Yes | 550 (98%) | 530 (96%) | 1080 (97%) |
| Missing | 52 | 57 | 109*** |
| **Type of school** |  |  |  |
| Mainstream school or college | 520 (96%) | 511 (97%) | 1031 (96%) |
| Special unit in mainstream school | 6 (1%) | 4 (1%) | 10 (1%) |
| Special needs school | 10 (2%) | 8 (2%) | 18 (2%) |
| Home educated | 0 (0%) | 1 (<1%) | 1 (<1%) |
| Other | 8 (1%) | 5 (1%) | 13 (1%) |
| Missing | 71 | 81 | 152*** |
| **Doing an apprenticeship** |  |  |  |
| No | 614 (100%) | 609 (100%) | 1223 (100%) |
| Yes | 1 (<1%) | 1 (<1%) | 2 (<1%) |
| **In paid employment** |  |  |  |
| No | 602 (98%) | 596 (98%) | 1198 (98%) |
| Yes | 12 (2%) | 14 (2%) | 26 (2%) |
| Missing | 1 | 0 | 1 |
| **Index of Multiple Deprivation quintile (child’s primary residence)** |  |  |  |
| 1st quintile (most deprived) | 112 (18%) | 102 (17%) | 214 (17%) |
| 2nd quintile | 116 (19%) | 122 (20%) | 238 (19%) |
| 3rd quintile | 143 (23%) | 132 (22%) | 275 (22%) |
| 4th quintile | 105 (17%) | 104 (17%) | 209 (17%) |
| 5th quintile (least deprived) | 138 (22%) | 149 (24%) | 287 (23%) |
| Missing | 1 | 1 | 2 |
| **Prior CAMHS referral****** |  |  |  |
| No | 416 (68%) | 429 (70%) | 845 (69%) |
| Yes | 199 (32%) | 181 (30%) | 380 (31%) |
| **Previous or existing diagnosis of an emotional disorder in CAMHS records** |  |  |  |
| No | 577 (94%) | 572 (94%) | 1149 (94%) |
| Yes | 38 (6%) | 38 (6%) | 76 (6%) |

All data are N (%) unless otherwise indicated. *Collected from CAMHS records **Self-reported by participant. ***Missing data primarily for participants recruited prior to 21-Aug-2020 due to incorrect wording of participant questionnaires prior to this. ****Predominantly self-reported by participant, however if this was missing then this was augmented using data from CAMHS records.

### Baseline characteristics of the parent/carer

**Table 2.4: Parent/carer baseline characteristics data**

| **Characteristic** | **Intervention Group**  **(n = 560)** | **Control Group**  **(n = 561)** | **Total randomised (n = 1121)** |
| --- | --- | --- | --- |
| **Relationship to child** |  |  |  |
| Mother | 510 (91%) | 521 (93%) | 1031 (92%) |
| Father | 41 (7%) | 27 (5%) | 68 (6%) |
| Grandparent | 6 (1%) | 4 (1%) | 10 (1%) |
| Other | 2 (<1%) | 6 (1%) | 8 (1%) |
| Missing | 1 | 3 | 4 |
| **Age at randomisation (years)** |  |  |  |
| < 20 | 1 (<1%) | 2 (<1%) | 3 (<1%) |
| 20-29 | 22 (4%) | 31 (6%) | 53 (5%) |
| 30-39 | 208 (37%) | 176 (31%) | 384 (34%) |
| 40-49 | 249 (44%) | 254 (45%) | 503 (45%) |
| 50-59 | 76 (14%) | 88 (16%) | 164 (15%) |
| 60 or over | 4 (1%) | 8 (1%) | 12 (1%) |
| Missing | 0 | 2 | 2 |
| **Gender** |  |  |  |
| Male | 44 (8%) | 31 (6%) | 75 (7%) |
| Female | 516 (92%) | 528 (94%) | 1044 (93%) |
| Missing | 0 | 2 | 2 |
| **Ethnicity** |  |  |  |
| White | 514 (92%) | 508 (91%) | 1022 (91%) |
| Indian | 8 (1%) | 13 (2%) | 21 (2%) |
| Pakistani | 8 (1%) | 6 (1%) | 14 (1%) |
| Black Caribbean | 6 (1%) | 6 (1%) | 12 (1%) |
| Black African | 0 (0%) | 1 (<1%) | 1 (<1%) |
| Black (other) | 0 (0%) | 2 (<1%) | 2 (<1%) |
| Chinese | 2 (<1%) | 0 (0%) | 2 (<1%) |
| Other Asian | 4 (1%) | 6 (1%) | 10 (1%) |
| Dual/mixed heritage | 11 (2%) | 10 (2%) | 21 (2%) |
| Other | 6 (1%) | 6 (1%) | 12 (1%) |
| Missing | 1 | 3 | 4 |

All data are N (%) unless otherwise indicated.

Table 2.5: Child baseline assessment data (self-reported)

| **Characteristic** | **Intervention Group**  **(n = 252)** | **Control Group**  **(n = 250)** | **Total**  **(n=502)** |
| --- | --- | --- | --- |
| Depression symptoms (MFQ) |  |  |  |
| Mean[sd] | 38.7 [13.8] | 37.7 [13.3] | 38.2 [13.5] |
| Median [25^th^, 75^th^ centile] | 40 [30, 50] | 39 [27, 48] | 40 [28, 49] |
| Min, max | 2, 64 | 4, 62 | 2, 64 |
| n | 237 | 236 | 473 |
| Anxiety symptoms (RCADS) |  |  |  |
| Mean[sd] | 55.8 [21.9] | 57.7 [20.6] | 56.7 [21.2] |
| Median [25^th^, 75^th^ centile] | 57 [43, 70] | 57 [43, 74] | 57 [43, 72] |
| Min, max | 2, 100 | 9, 111 | 2, 111 |
| n | 237 | 236 | 473 |
| Separation anxiety disorder (RCADS) |  |  |  |
| Mean[sd] | 7.7 [4.6] | 8.1 [4.5] | 7.9 [4.5] |
| Median [25^th^, 75^th^ centile] | 7 [4, 11] | 7 [5, 11] | 7 [5, 11] |
| Min, max | 0, 20 | 0, 21 | 0, 21 |
| n | 238 | 236 | 474 |
| Social phobia (RCADS) |  |  |  |
| Mean[sd] | 17.6 [6.7] | 18.5 [6.4] | 18 [6.6] |
| Median [25^th^, 75^th^ centile] | 19 [13, 23] | 20 [14, 24] | 19 [14, 24] |
| Min, max | 0, 27 | 0, 27 | 0, 27 |
| n | 237 | 236 | 473 |
| Generalized anxiety disorder (RCADS) |  |  |  |
| Mean[sd] | 10.2 [4.7] | 10.3 [4.1] | 10.2 [4.4] |
| Median [25^th^, 75^th^ centile] | 10 [7, 14] | 11 [7, 13] | 10 [7, 14] |
| Min, max | 0, 18 | 1, 18 | 0, 18 |
| n | 238 | 236 | 474 |
| Panic disorder (RCADS) |  |  |  |
| Mean[sd] | 12.5 [6.9] | 12.8 [7] | 12.7 [7] |
| Median [25^th^, 75^th^ centile] | 12 [7, 17] | 13 [7.5, 18.5] | 13 [7, 18] |
| Min, max | 0, 27 | 0, 27 | 0, 27 |
| n | 238 | 236 | 474 |
| Obsessive compulsive disorder (RCADS) |  |  |  |
| Mean[sd] | 7.9 [4.5] | 8 [4.4] | 7.9 [4.4] |
| Median [25^th^, 75^th^ centile] | 8 [4, 11] | 7.5 [5, 11] | 8 [4, 11] |
| Min, max | 0, 18 | 0, 18 | 0, 18 |
| n | 237 | 236 | 473 |
| Low mood (major depressive disorder) (RCADS) |  |  |  |
| Mean[sd] | 17 [6.7] | 16.4 [6.6] | 16.7 [6.6] |
| Median [25^th^, 75^th^ centile] | 18 [12, 22] | 16.5 [11.5, 21] | 17 [12, 22] |
| Min, max | 0, 30 | 1, 30 | 0, 30 |
| n | 238 | 236 | 474 |
| Total Internalising Scale (RCADS) |  |  |  |
| Mean[sd] | 72.8 [27] | 74.1 [25.5] | 73.5 [26.2] |
| Median [25^th^, 75^th^ centile] | 75 [58, 89.7] | 75 [57, 93] | 75 [57, 92] |
| Min, max | 2, 127 | 13, 140 | 2, 140 |
| n | 237 | 236 | 473 |
| Emotional symptoms subscale (SDQ) |  |  |  |
| Mean[sd] | 7.1 [2.4] | 7 [2.2] | 7 [2.3] |
| Median [25^th^, 75^th^ centile] | 8 [6, 9] | 7 [6, 9] | 7 [6, 9] |
| Min, max | 0, 10 | 1, 10 | 0, 10 |
| n | 150 | 234 | 384 |
| Oppositional defiant / conduct disorder subscale (SDQ conduct problems) |  |  |  |
| Mean[sd] | 3.2 [2.1] | 2.9 [2.1] | 3 [2.1] |
| Median [25^th^, 75^th^ centile] | 3 [2, 5] | 3 [1, 4] | 3 [1, 4] |
| Min, max | 0, 9 | 0, 10 | 0, 10 |
| n | 150 | 234 | 384 |
| Hyperactivity/inattention subscale (SDQ) |  |  |  |
| Mean[sd] | 6.4 [2.4] | 6.3 [2.5] | 6.3 [2.4] |
| Median [25^th^, 75^th^ centile] | 7 [5, 8] | 6 [5, 9] | 6 [5, 8] |
| Min, max | 0, 10 | 0, 10 | 0, 10 |
| n | 150 | 234 | 384 |
| Peer relationships problem subscale (SDQ) |  |  |  |
| Mean[sd] | 3.6 [2.2] | 3.8 [2.1] | 3.7 [2.1] |
| Median [25^th^, 75^th^ centile] | 3 [2, 5] | 4 [2, 5] | 4 [2, 5] |
| Min, max | 0, 8 | 0, 10 | 0, 10 |
| n | 150 | 234 | 384 |
| Total symptoms (SDQ) |  |  |  |
| Mean[sd] | 20.3 [5.8] | 19.9 [5.7] | 20 [5.8] |
| Median [25^th^, 75^th^ centile] | 20 [16, 24] | 20 [16, 24] | 20 [16, 24] |
| Min, max | 5, 34 | 2, 35 | 2, 35 |
| n | 150 | 234 | 384 |
| Prosocial behaviour (SDQ) |  |  |  |
| Mean[sd] | 6.9 [2.1] | 6.7 [2.3] | 6.8 [2.2] |
| Median [25^th^, 75^th^ centile] | 7 [6, 8] | 7 [5, 8] | 7 [5, 8] |
| Min, max | 1, 10 | 0, 10 | 0, 10 |
| n | 150 | 234 | 384 |
| Functional Impairment (SDQ) |  |  |  |
| Mean[sd] | 4.9 [2.6] | 3.9 [2.5] | 4.2 [2.6] |
| Median [25^th^, 75^th^ centile] | 5 [3, 7] | 4 [2, 6] | 4 [2, 6] |
| Min, max | 0, 10 | 0, 10 | 0, 10 |
| n | 139 | 231 | 370 |

*Secondary outcomes are highlighted

Table 2.6: Child baseline assessment data (parent/carer-reported)

| **Characteristic** | **Intervention Group**  **(n = 560)** | **Control Group**  **(n = 561)** | **Total**  **(n=1121)** |
| --- | --- | --- | --- |
| Depression symptoms (MFQ) |  |  |  |
| Mean[sd] | 31.6 [13.3] | 31.5 [14.1] | 31.5 [13.7] |
| Median [25^th^, 75^th^ centile] | 32.5 [22, 41] | 31 [20, 43] | 32 [21, 42] |
| Min, max | 0, 63 | 1, 61 | 0, 63 |
| n | 556 | 554 | 1110 |
| Anxiety symptoms (RCADS) |  |  |  |
| Mean[sd] | 45.3 [19.7] | 46.6 [20.8] | 46 [20.3] |
| Median [25^th^, 75^th^ centile] | 44 [30, 60] | 46 [31, 61] | 45 [31, 60] |
| Min, max | 4, 99 | 1, 106 | 1, 106 |
| n | 553 | 548 | 1101 |
| Separation anxiety disorder (RCADS) |  |  |  |
| Mean[sd] | 8.6 [5.3] | 8.9 [5.2] | 8.8 [5.3] |
| Median [25^th^, 75^th^ centile] | 8 [4, 12] | 8.2 [5, 13] | 8 [4, 13] |
| Min, max | 0, 21 | 0, 21 | 0, 21 |
| n | 557 | 556 | 1113 |
| Social phobia (RCADS) |  |  |  |
| Mean[sd] | 15.2 [6.6] | 15.4 [6.7] | 15.3 [6.6] |
| Median [25^th^, 75^th^ centile] | 15 [10, 20] | 15 [10, 20] | 15 [10, 20] |
| Min, max | 0, 27 | 0, 27 | 0, 27 |
| n | 558 | 551 | 1109 |
| Generalized anxiety disorder (RCADS) |  |  |  |
| Mean[sd] | 8.6 [4.3] | 8.8 [4.4] | 8.7 [4.4] |
| Median [25^th^, 75^th^ centile] | 8 [5, 12] | 8 [6, 12] | 8 [5, 12] |
| Min, max | 0, 18 | 0, 18 | 0, 18 |
| n | 558 | 554 | 1112 |
| Panic disorder (RCADS) |  |  |  |
| Mean[sd] | 7.7 [5.5] | 8.4 [6.1] | 8 [5.8] |
| Median [25^th^, 75^th^ centile] | 7 [4, 11] | 7 [3, 13] | 7 [3.4, 11.6] |
| Min, max | 0, 27 | 0, 27 | 0, 27 |
| n | 558 | 550 | 1108 |
| Obsessive compulsive disorder (RCADS) |  |  |  |
| Mean[sd] | 5.3 [4.1] | 5.2 [4] | 5.2 [4.1] |
| Median [25^th^, 75^th^ centile] | 4 [2, 7.5] | 4.8 [2, 7] | 4 [2, 7] |
| Min, max | 0, 18 | 0, 18 | 0, 18 |
| n | 556 | 555 | 1111 |
| Low mood (major depressive disorder) (RCADS) |  |  |  |
| Mean[sd] | 13.2 [6] | 12.9 [6.2] | 13 [6.1] |
| Median [25^th^, 75^th^ centile] | 13 [9, 17] | 12.5 [8, 17] | 13 [9, 17] |
| Min, max | 0, 29 | 0, 30 | 0, 30 |
| n | 556 | 552 | 1108 |
| Total Internalising Scale (RCADS) |  |  |  |
| Mean[sd] | 58.5 [23.9] | 59.5 [25.4] | 59 [24.6] |
| Median [25^th^, 75^th^ centile] | 58.3 [41, 74] | 59 [40.6, 76] | 59 [41, 75] |
| Min, max | 4, 123 | 2, 125 | 2, 125 |
| n | 551 | 548 | 1099 |
| Emotional symptoms subscale (SDQ) |  |  |  |
| Mean[sd] | 6.9 [2.4] | 6.5 [2.5] | 6.7 [2.4] |
| Median [25^th^, 75^th^ centile] | 7 [5, 9] | 7 [5, 8.8] | 7 [5, 9] |
| Min, max | 0, 10 | 0, 10 | 0, 10 |
| n | 425 | 554 | 979 |
| Oppositional defiant / conduct disorder subscale (SDQ conduct problems) |  |  |  |
| Mean[sd] | 3.5 [2.3] | 3.5 [2.5] | 3.5 [2.4] |
| Median [25^th^, 75^th^ centile] | 3 [2, 5] | 3 [2, 5] | 3 [2, 5] |
| Min, max | 0, 10 | 0, 10 | 0, 10 |
| n | 425 | 554 | 979 |
| Hyperactivity/inattention subscale (SDQ) |  |  |  |
| Mean[sd] | 6.1 [2.6] | 5.9 [2.8] | 5.9 [2.8] |
| Median [25^th^, 75^th^ centile] | 6 [4, 8] | 6 [4, 8] | 6 [4, 8] |
| Min, max | 0, 10 | 0, 10 | 0, 10 |
| n | 425 | 554 | 979 |
| Peer relationships problem subscale (SDQ) |  |  |  |
| Mean[sd] | 3.8 [2.3] | 3.9 [2.4] | 3.9 [2.4] |
| Median [25^th^, 75^th^ centile] | 4 [2, 6] | 4 [2, 6] | 4 [2, 6] |
| Min, max | 0, 10 | 0, 10 | 0, 10 |
| n | 425 | 554 | 979 |
| Total symptoms (SDQ) |  |  |  |
| Mean[sd] | 20.3 [6.7] | 19.8 [7.1] | 20 [6.9] |
| Median [25^th^, 75^th^ centile] | 21 [16, 25] | 20 [15, 25] | 20 [15, 25] |
| Min, max | 1, 36 | 1, 37 | 1, 37 |
| n | 425 | 554 | 979 |
| Prosocial behaviour (SDQ) |  |  |  |
| Mean[sd] | 6.2 [2.6] | 6.3 [2.7] | 6.3 [2.6] |
| Median [25^th^, 75^th^ centile] | 6 [4, 8] | 7 [4, 9] | 7 [4, 8] |
| Min, max | 0, 10 | 0, 10 | 0, 10 |
| n | 425 | 554 | 979 |
| Functional Impairment (SDQ) |  |  |  |
| Mean[sd] | 5.8 [2.8] | 4.9 [2.9] | 5.3 [2.9] |
| Median [25^th^, 75^th^ centile] | 6 [4, 8] | 5 [3, 7] | 5 [3, 8] |
| Min, max | 0, 10 | 0, 10 | 0, 10 |
| n | 424 | 551 | 975 |

Table 2.7: Parent/carer self-report baseline assessment data

|  | **Intervention Group**  **(n = 560)** | **Control Group**  **(n = 561)** | **Total**  **(n=1121)** |
| --- | --- | --- | --- |
| Depression symptoms (PHQ-9) |  |  |  |
| Mean[sd] | 9.5 [6.6] | 9.1 [6.3] | 9.3 [6.4] |
| Median [25^th^, 75^th^ centile] | 8 [4, 14] | 8 [4, 14] | 8 [4, 14] |
| Min, max | 0, 27 | 0, 27 | 0, 27 |
| n | 557 | 555 | 1112 |
| Anxiety symptoms (GAD-7) |  |  |  |
| Mean[sd] | 8.7 [6] | 8.3 [5.9] | 8.5 [5.9] |
| Median [25^th^, 75^th^ centile] | 8 [4, 13] | 7 [3.5, 13] | 7 [4, 13] |
| Min, max | 0, 21 | 0, 21 | 0, 21 |
| n | 559 | 556 | 1115 |

Table 2.8: Child self-harm (self-report) at baseline

|  | **Intervention Group**  **(n = 252)** | **Control Group**  **(n = 250)** | **Total**  **(n=502)** |
| --- | --- | --- | --- |
| Have you thought about hurting yourself, even if you would not do it, in the last 6 months? |  |  |  |
| Not at all | 59 (25%) | 75 (32%) | 134 (28%) |
| Once or twice | 72 (30%) | 55 (23%) | 127 (27%) |
| Three or more times | 106 (45%) | 105 (45%) | 211 (45%) |
| Missing | 15 | 15 | 30 |
| Have you hurt yourself on purpose in anyway in the last 6 months? |  |  |  |
| Not at all | 132 (58%) | 151 (67%) | 283 (63%) |
| Once | 28 (12%) | 20 (9%) | 48 (11%) |
| Two or more times | 66 (29%) | 55 (24%) | 121 (27%) |
| Missing | 26 | 24 | 50 |

## Study quality summaries

### Process outcomes

Table 2.9: Process outcomes

|  | **Intervention Group**  **(n = 615)** | **Control Group**  **(n = 610)** | **Total**  **(n = 1225)** |
| --- | --- | --- | --- |
| **Time from referral receipt to randomisation (days)** |  |  |  |
| Mean [sd] | 7.5 [4] | 7.6 [4.1] | 7.6 [4.1] |
| Median [25^th^, 75^th^ centile] | 7 [4, 11] | 7 [5, 11] | 7 [4, 11] |
| Min, max | 1, 27 | 1, 33 | 1, 33 |
| n | 615 | 610 | 1225 |
| **Time from randomisation to referral decision (days)*** |  |  |  |
| Mean [sd] | 33.9 [58.3] | 32.8 [61.6] | 33.4 [60] |
| Median [25^th^, 75^th^ centile] | 13 [7, 30] | 11.5 [4, 30] | 12 [6, 30] |
| Min, max | -13, 450 | -13, 392 | -13, 450 |
| n | 614 | 610 | 1224 |

All data are N (%) unless otherwise indicated. *In a minority of cases (n= 34) the referral decision was made before randomisation.

### Adherence to the intervention

**Table 2.10: Adherence to intervention**

|  | **Intervention Group** |
| --- | --- |
|  | **(n = 615)** |
| **DAWBA fully or partially completed by either child or parent/carer** |  |
| Yes | 494 (80%) |
| No | 121 (20%) |
| **DAWBA report generated** |  |
| Yes | 494 (80%) |
| No | 121 (20%) |
| *Reason* |  |
| *DAWBA not completed sufficiently to generate any diagnostic predictions* | 107 (88%) |
| *Participant withdrew consent* | 3 (2%) |
| *Other* | 11 (9%) |
|  |  |
| **DAWBA report sent to primary participant** |  |
| Yes | 493 (80%) |
| No | 122 (20%) |
| *Reason* |  |
| *DAWBA not completed* | 40 (33%) |
| *DAWBA not generated* | 74 (61%) |
| *Participant withdrew consent* | 3 (2%) |
| *Unknown* | 4 (3%) |
| *Other* | 1 (1%) |
|  |  |
| **DAWBA report uploaded to CAMHS records** |  |
| Yes | 490 (80%) |
| No | 125 (20%) |
| *Reason* |  |
| *DAWBA not completed* | 41 (33%) |
| *DAWBA not generated* | 75 (60%) |
| *Participant withdrew consent* | 3 (2%) |
| *Unknown* | 2 (2%) |
| *Other* | 4 (3%) |
|  |  |
| **Time from randomisation to DAWBA report uploaded to CAMHS records (days)** |  |
| Mean [sd] | 6.3 [6.9] |
| Median [25^th^, 75^th^ centile] | 5 [3, 8] |
| Min, max | 0, 72 |
| n | 490 |

All data are N (%) unless otherwise indicated.

**Table 2.11: DAWBA Summary**

|  | **n** | **Close to Average** | **Slightly raised** | **High** | **Very High** |
| --- | --- | --- | --- | --- | --- |
| **Separation Anxiety** | 461 | 223 (48%) | 178 (39%) | 1 (<1%) | 59 (13%) |
| **Specific Phobias** | 479 | 323 (67%) | 1 (<1%) | 52 (11%) | 103 (22%) |
| **Social Phobia** | 478 | 187 (39%) | 90 (19%) | 87 (18%) | 114 (24%) |
| **Panic Attacks** | 478 | 298 (62%) | 84 (18%) | 65 (14%) | 31 (6%) |
| **Agoraphobia** | 478 | 277 (58%) | 97 (20%) | 81 (17%) | 23 (5%) |
| **Generalised Anxiety** | 479 | 48 (10%) | 141 (29%) | 116 (24%) | 174 (36%) |
| **Obsessive Compulsive Disorder** | 480 | 368 (77%) | 41 (9%) | 46 (10%) | 25 (5%) |
| **Post-Traumatic Stress Disorder** | 477 | 410 (86%) | 20 (4%) | 34 (7%) | 13 (3%) |
| **Depression** | 484 | 186 (38%) | 10 (2%) | 93 (19%) | 195 (40%) |
| **At least one emotional disorder domain scoring “Very High”*** | 494 |  |  |  | 332 (67%) |
| **Oppositional Defiant Disorder** | 428 | 74 (17%) | 101 (24%) | 90 (21%) | 163 (38%) |
| **Conduct Disorder** | 474 | 235 (50%) | 132 (28%) | 40 (8%) | 67 (14%) |

All data are N (%) unless otherwise indicated. *ODD and CD not included and denominator is the number of participants who completed/ partially completed a DAWBA

NB: DAWBA questions reflect ICD-10 & DSM-IV; the algorithm is based on ICD-10 diagnostic criteria

### Completeness of follow-up

**Table 2.12: Summary of baseline and follow up questionnaires for child/young person**

|  | **Baseline** | | **6 months** | | **12 months** | |
| --- | --- | --- | --- | --- | --- | --- |
|  | **Intervention Group (n = 252)** | **Control Group (n = 250)** | **Intervention Group (n = 252)** | **Control Group (n = 250)** | **Intervention Group (n = 252)** | **Control Group (n = 250)** |
| Questionnaire returned | 244 (97%) | 241 (96%) | 140 (56%) | 140 (56%) | 157 (62%) | 156 (62%) |
| Reason not returned |  |  |  |  |  |  |
| No response | 8 (100%) | 9 (100%) | 111 (99%) | 110 (100%) | 84 (88%) | 89 (95%) |
| Withdrawn* | 0 (0%) | 0 (0%) | 1 (1%) | 0 (0%) | 11 (12%) | 5 (5%) |
| Time from randomisation to follow up questionnaire finished (days) |  |  |  |  |  |  |
| Mean[sd] | .4 [1.4] | .4 [1.4] | 212.2 [28.5] | 207.5 [23.8] | 389.9 [27] | 390.8 [23.9] |
| Median [25^th^, 75^th^ centile] | 0 [0, 0] | 0 [0, 0] | 204 [186, 234] | 201 [186, 222] | 382 [366, 405] | 386 [368, 406] |
| Min, max** | -3, 9 | -4, 10 | 181, 278 | 181, 270 | 365, 457 | 365, 451 |
| n | 236 | 235 | 134 | 117 | 141 | 145 |

All data are N (%) unless otherwise indicated. *Withdrawn from questionnaire completion **Baseline questionnaires were completed by the primary participant before randomisation. Children aged 11-15 were optional secondary participants and may have also completed their baseline questionnaire before or after randomisation.

**Table 2.13: Summary of baseline and follow up questionnaires for parent/carer**

|  | **Baseline** | | **6 months** | | **12 months** | |
| --- | --- | --- | --- | --- | --- | --- |
|  | **Intervention Group (n = 560)** | **Control Group (n = 561)** | **Intervention Group (n = 560)** | **Control Group (n = 561)** | **Intervention Group (n = 560)** | **Control Group (n = 561)** |
| Questionnaire returned | 560 (100%) | 559 (100%) | 434 (78%) | 440 (78%) | 431 (77%) | 434 (77%) |
| Reason not returned |  |  |  |  |  |  |
| No response | 0 (0%) | 2 (100%) | 122 (97%) | 121 (100%) | 108 (84%) | 112 (88%) |
| Withdrawn* | 0 (0%) | 0 (0%) | 4 (3%) | 0 (0%) | 21 (16%) | 15 (12%) |
| Time from randomisation to follow up questionnaire completion (days) |  |  |  |  |  |  |
| Mean[sd] | .1 [.4] | 0 [.4] | 212.2 [26] | 211.5 [26.4] | 391.8 [25.7] | 388.9 [23.9] |
| Median [25^th^, 75^th^ centile] | 0 [0, 0] | 0 [0, 0] | 207 [190, 226] | 206.5 [188, 227] | 385 [368, 406.5] | 384 [367, 401] |
| Min, max** | 0, 6 | 0, 8 | 181, 280 | 181, 292 | 365, 458 | 365, 462 |
| n | 557 | 554 | 378 | 384 | 400 | 397 |
| Relationship to child |  |  |  |  |  |  |
| Mother | 510 (91%) | 521 (93%) | 510 (91%) | 521 (93%) | 510 (91%) | 521 (93%) |
| Father | 41 (7%) | 27 (5%) | 41 (7%) | 27 (5%) | 41 (7%) | 27 (5%) |
| Grandparent | 6 (1%) | 4 (1%) | 6 (1%) | 4 (1%) | 6 (1%) | 4 (1%) |
| Missing | 2 (0%) | 6 (1%) | 2 (0%) | 6 (1%) | 2 (0%) | 6 (1%) |

All data are N (%) unless otherwise indicated. *Withdrawn from questionnaire completion. **Baseline questionnaires were completed by the primary participant before randomisation. Parents of children aged 16-17 were optional secondary participants and may have also completed their baseline questionnaire before or after randomisation.

**Table 2.14: Completeness of baseline and follow up questionnaires for child/young person (self-reported – 11+)**

|  | **Baseline** | | **6 months** | | **12 months** | |
| --- | --- | --- | --- | --- | --- | --- |
|  | **Intervention Group (n = 252)** | **Control Group (n = 250)** | **Intervention Group (n = 252)** | **Control Group (n = 250)** | **Intervention Group (n = 252)** | **Control Group (n = 250)** |
| Questionnaire completed |  |  |  |  |  |  |
| SDQ* | 150 (60%) | 234 (94%) | 135 (54%) | 125 (50%) | 147 (58%) | 150 (60%) |
| MFQ | 237 (94%) | 236 (94%) | 135 (54%) | 120 (48%) | 147 (58%) | 146 (58%) |
| RCADS | 237 (94%) | 236 (94%) | 135 (54%) | 119 (48%) | 144 (57%) | 145 (58%) |

All data are N (%) unless otherwise indicated. *Participants in the intervention group completed the Strengths and Difficulties Questionnaire (SDQ) as part of the DAWBA, therefore this questionnaire was omitted from the intervention group baseline questionnaire.

**Table 2.15: Completeness of baseline and follow up questionnaires for child/young person (parent/carer-reported)**

|  | **Baseline** | | **6 months** | | **12 months** | |
| --- | --- | --- | --- | --- | --- | --- |
|  | **Intervention Group (n = 60)** | **Control Group (n = 561)** | **Intervention Group (n = 560)** | **Control Group (n = 561)** | **Intervention Group (n = 560)** | **Control Group (n = 561)** |
| Questionnaire completed |  |  |  |  |  |  |
| SDQ | 425 (76%) | 554 (99%) | 410 (73%) | 417 (74%) | 412 (74%) | 412 (73%) |
| MFQ | 556 (99%) | 554 (99%) | 404 (72%) | 405 (72%) | 404 (72%) | 404 (72%) |
| RCADS | 551 (98%) | 548 (98%) | 393 (70%) | 391 (70%) | 399 (71%) | 403 (72%) |

All data are N (%) unless otherwise indicated. *Participants in the intervention group completed the Strengths and Difficulties Questionnaire (SDQ) as part of the DAWBA, therefore this questionnaire was omitted from the intervention group baseline questionnaire.

**Table 2.16: Completeness of baseline and follow up questionnaires for parent/carers**

|  | **Baseline** | | **6 months** | | **12 months** | |
| --- | --- | --- | --- | --- | --- | --- |
|  | **Intervention Group (n = 560)** | **Control Group (n = 561)** | **Intervention Group (n = 560)** | **Control Group (n = 561)** | **Intervention Group (n = 560)** | **Control Group (n = 561)** |
| Questionnaire completed |  |  |  |  |  |  |
| PHQ-9 | 557 (99%) | 555 (99%) | 385 (69%) | 388 (69%) | 402 (72%) | 401 (71%) |
| GAD-7 | 559 (100%) | 556 (99%) | 383 (68%) | 386 (69%) | 400 (71%) | 401 (71%) |
| EQ5D5L | 554 (99%) | 550 (98%) | 380 (68%) | 385 (69%) | 397 (71%) | 398 (71%) |

All data are N (%) unless otherwise indicated.

**Table 2.17: Completeness of health economics questionnaires for child/young person (self- or parent/carer- completed)**

|  | **Baseline** | | **6 months** | | **12 months** | |
| --- | --- | --- | --- | --- | --- | --- |
|  | **Intervention Group (n = 615)** | **Control Group (n = 610)** | **Intervention Group (n = 615)** | **Control Group (n = 610)** | **Intervention Group (n = 615)** | **Control Group (n = 610)** |
| Questionnaire completed |  |  |  |  |  |  |
| EQ5DY | 565 (92%) | 558 (91%) | 433 (70%) | 433 (71%) | 439 (71%) | 440 (72%) |
| CHU9 | 565 (92%) | 557 (91%) | 432 (70%) | 432 (71%) | 440 (72%) | 442 (72%) |

All data are N (%) unless otherwise indicated.

## Primary and secondary outcomes

### Primary analysis for the primary outcome

Table 2.18: Primary outcome

|  | **Intervention Group**  **(n = 615)** | **Control Group**  **(n = 610)** | **Adjusted risk ratio***  **(95% CI, p-value)** | **Adjusted risk difference***  **(95% CI)** |
| --- | --- | --- | --- | --- |
| **Primary outcome** |  |  |  |  |
|  |  |  |  |  |
| **Clinician-made diagnosis decision about the presence of an emotional disorder within 12 months of randomisation** |  |  |  |  |
| No (Either confirmed absence of disorder, or  uncertainty about the presence of an emotional disorder) | 542 (89%) | 537 (88%) | 0.94 (0.70, 1.28),  p = 0.710 | -0.63 (-3.99, 2.72) |
| Yes (Confirmed presence of disorder) | 68 (11%) | 72 (12%) |  |  |
| Missing | 5 | 1 |  |  |
|  |  |  |  |  |
| **Secondary outcomes** |  |  |  |  |
| **Confirmed diagnosis decision (diagnosis of an emotional disorder or confirmed absence of an emotional disorder) within 12 months** |  |  |  |  |
| No (neither confirmed present nor absent) | 516 (85%) | 520 (85%) | 1.07 (0.82, 1.39),  p = 0.625 | 0.92 (-2.77, 4.61) |
| Yes (confirmed present or absent) | 94 (15%) | 89 (15%) |  |  |
| Missing | 5 | 1 |  |  |
| **Diagnosis of emotional disorder (categorical)** |  |  |  |  |
| 1) Emotional disorder diagnosis documented in clinical records | 68 (11%) | 72 (12%) |  |  |
| 2) Having a clearly documented absence of an emotional disorder | 26 (4%) | 17 (3%) |  |  |
| 3) Uncertainty in presence of an emotional disorder | 170 (28%) | 135 (22%) |  |  |
| 4) No diagnostic information | 346 (57%) | 385 (63%) |  |  |
| Missing | 5 | 1 |  |  |
| **Time from randomisation to diagnosis of emotional disorder (days)** |  |  |  |  |
| Mean[sd] | 108.1 [109.7] | 99.8 [104.3] | HR**: 0.92 (0.66, 1.29) | |
| Median [25^th^, 75^th^ centile] | 54.5 [26, 168.5] | 47.5 [27, 143.5] |  |  |
| Min, max | 2, 356 | 3, 354 |  |  |
| n | 68 | 72 |  |  |

Data are n (%) unless otherwise indicated.

*Adjusted by site and other minimisation factors. **All participants are included in the Cox model; however, participants are censored at point of withdrawal if they withdrew prior to diagnosis or 12 months post-randomisation. HR: Hazard Ratio.

Figure 2.2: Time to first diagnosis of an emotional disorder within 12 months

### Subgroup analysis for the primary outcome

Table 2.19: Subgroup analysis of primary outcome

|  | **Intervention Group**  **(n = 615)** | **Control Group**  **(n = 610)** | **Adjusted risk ratio* (95% CI)** | **Adjusted interaction effect****  **(95% CI)** | **p-value for interaction** |
| --- | --- | --- | --- | --- | --- |
| **By sex***** |  |  |  |  |  |
| Female | 47/355 (13%) | 51 /355 (14%) | 0.93 (0.65, 1.34) | 0.97 (0.42, 2.11) | 0.891 |
| Male | 21/255 (8%) | 21/254 (8%) | 0.97 (0.56, 1.68) |  |  |
| **By age****** |  |  |  |  |  |
| 5–10 years | 7/212 (3%) | 12/209 (6%) | 0.65 (0.27, 1.58) | 1.62 (0.56, 4.62) | 0.371 |
| 11–17 years | 61/398 (15%) | 60/400 (15%) | 1.01 (0.73, 1.39) |  |  |

*Adjusted by site and other minimisation factors.

**Odds ratio taken from interaction term within the model. ***Missing outcome data for one female in the control group, two males and three females in the intervention group. ****Missing outcome data for one 5–10-year-old in the control group and five 11–17-year-olds in the intervention group.

### Secondary analysis for the primary outcome

Table 2.20: Secondary analysis of primary outcome (ordinal analysis)

|  | **Intervention Group**  **(n = 615)** | **Control Group**  **(n = 610)** | **Common odds ratio***  **(95% CI, p-value)** |
| --- | --- | --- | --- |
|  |  |  |  |
| **Clinician-made diagnosis decision about the presence of an emotional disorder** **within 12 months of** **randomisation** |  |  |  |
| Referral** not accepted | 261 (43%) | 270 (44%) | 1.07 (0.86, 1.33), |
| Referral accepted but no confirmed diagnosis | 281 (46%) | 267 (44%) | p = 0.557 |
| Confirmed diagnosis | 68 (11%) | 72 (12%) |  |
| Missing | 5 | 1 |  |
|  |  |  |  |

Data are n (%) unless otherwise indicated. *Adjusted by site and other minimisation factors. **Any referral accepted before 12 months

### Secondary outcomes

Table 2.21: Clinician-made diagnosis decision about the presence of an emotional disorder within 18 months of randomisation

|  | **Intervention Group**  **(n = 615)** | **Control Group**  **(n = 610)** | **Adjusted risk ratio***  **(95% CI, p-value)** | **Adjusted risk difference***  **(95% CI)** |
| --- | --- | --- | --- | --- |
| **Secondary outcomes** |  |  |  |  |
|  |  |  |  |  |
| **Clinician-made diagnosis decision about the presence of an emotional disorder within 18 months of randomisation** |  |  |  |  |
| No (Either confirmed absence of disorder, or  uncertainty about the presence of an emotional disorder) | 525 (86%) | 515 (85%) | 0.92 (0.71, 1.19,  p = 0.520 | -1.25 (-5.06, 2.57) |
| Yes (Confirmed presence of disorder) | 84 (14%) | 91 (15%) |  |  |
| Missing | 6 | 4 |  |  |
| **Confirmed diagnosis decision (diagnosis of an emotional disorder or confirmed absence of an emotional disorder) within 18 months** |  |  |  |  |
| No (neither confirmed present nor absent) | 493 (81%) | 493 (81%) | 1.03 (0.82, 1.29), p = 0.793 | 0.55 (-3.55, 4.65) |
| Yes (confirmed present or absent) | 116 (19%) | 113 (19%) |  |  |
| Missing | 6 | 4 |  |  |
| **Diagnosis of emotional disorder (categorical)** |  |  |  |  |
| 1) Emotional disorder diagnosis documented in clinical records | 84 (14%) | 91 (15%) |  |  |
| 2) Having a clearly documented absence of an emotional disorder | 32 (5%) | 22 (4%) |  |  |
| 3) Uncertainty in presence of an emotional disorder | 179 (29%) | 144 (24%) |  |  |
| 4) No diagnostic information | 314 (52%) | 349 (58%) |  |  |
| Missing | 6 | 4 |  |  |
| **Time from randomisation to diagnosis of emotional disorder (days)** |  |  |  |  |
| Mean[sd] | 139.5 [143.1] | 140.1 [151.9] | HR**: 0.91 (0.67, 1.22) | |
| Median [25^th^, 75^th^ centile] | 69.5 [31.5, 248] | 62 [32, 233] |  |  |
| Min, max | 2, 512 | 3, 525 |  |  |
| n | 84 | 91 |  |  |

Data are n (%) unless otherwise indicated.

*Adjusted by site and other minimisation factors. ** All participants are included in the Cox model; however, participants are censored at point of withdrawal if they withdrew prior to diagnosis or 18 months post-randomisation.

Table 2.22: Secondary outcomes – referral acceptance within 12 months

Figure 2.3: Time to first diagnosis of an emotional disorder within 18 months

|  | **Intervention Group**  **(n = 615)** | **Control Group**  **(n = 610)** | **Adjusted* risk ratio**  **(95% CI)** | **Adjusted* risk difference**  **(95% CI)** |
| --- | --- | --- | --- | --- |
| **Acceptance of index referral** |  |  |  |  |
| No | 335 (55%) | 348 (57%) | 1.06 (0.94, 1.19) | 2.58 (-2.72, 7.89) |
| Yes | 277 (45%) | 262 (43%) |  |  |
| Missing | 3 | 0 |  |  |
| **Acceptance of any referral within 12 months** |  |  |  |  |
| No | 263 (43%) | 273 (45%) | 1.03 (0.94, 1.13) | 1.97 (-3.31, 7.25) |
| Yes | 349 (57%) | 337 (55%) |  |  |
| Missing | 3 | 0 |  |  |
| **Discharge from CAMHS within 12 months**** | **(n = 349)** | **(n = 337)** |  |  |
| No | 224 (64%) | 215 (64%) |  |  |
| Yes | 125 (36%) | 122 (36%) |  |  |
| **Re-referral to CAMHS within 12 months** |  |  |  |  |
| No | 436 (71%) | 454 (75%) |  |  |
| Yes | 174 (29%) | 155 (25%) |  |  |
| Missing | 5 | 1 |  |  |

Data are n (%) unless otherwise indicated.

*Adjusted by site and other minimisation factors. **Participants can only be discharged if they have had any referral accepted

**Table 2.23: Secondary outcomes – referral acceptance within 18 months**

|  | **Intervention Group**  **(n = 615)** | **Control Group**  **(n = 610)** | **Adjusted* risk ratio**  **(95% CI)** | **Adjusted* risk difference**  **(95% CI)** |
| --- | --- | --- | --- | --- |
| **Acceptance of any referral within 18 months** |  |  |  |  |
| No | 237 (39%) | 256 (42%) | 1.06 (0.97, 1.16) | 3.58 (-1.67, 8.83) |
| Yes | 374 (61%) | 352 (58%) |  |  |
| Missing | 4 | 2 |  |  |
| **Discharge from CAMHS within 18 months**** | **(n = 374)** | **(n = 352)** |  |  |
| No | 179 (48%) | 180 (51%) |  |  |
| Yes | 195 (52%) | 172 (49%) |  |  |
| **Re-referral to CAMHS within 18 months** |  |  |  |  |
| No | 382 (63%) | 405 (67%) |  |  |
| Yes | 227 (37%) | 201 (33%) |  |  |
| Missing | 6 | 4 |  |  |

Data are n (%) unless otherwise indicated.

*Adjusted by site and other minimisation factors. **Participants can only be discharged if they have had any referral accepted

Table 2.24: Secondary outcomes – treatment within 12 months

|  | **Intervention Group**  **(n = 615)** | **Control Group**  **(n = 610)** | **Adjusted* risk ratio****  **(95% CI)** | **Adjusted* risk difference**  **(95% CI)** |
| --- | --- | --- | --- | --- |
| **Any treatment offered**** for diagnosed emotional disorder within 12 months** |  |  |  |  |
| No | 577 (95%) | 571 (94%) | 0.87 (0.59, 1.27) | -0.93 (-3.49, 1.63) |
| Yes | 33 (5%) | 38 (6%) |  |  |
| Missing | 5 | 1 |  |  |
| **Time from randomisation to the decision to offer treatment for a diagnosed emotional disorder (weeks)** |  |  | HR*** 0.81 (0.51, 1.30) | |
| Mean[sd] | 26.2 [14.4] | 19.6 [14.5] |  |  |
| Median [25^th^, 75^th^ centile] | 23 [14, 37] | 15 [7, 33] |  |  |
| Min, max | 3, 52 | 1, 45 |  |  |
| n | 33 | 38 |  |  |
| **Any treatment started**** for diagnosed emotional disorder within 12 months** |  |  |  |  |
| No | 594 (97%) | 584 (96%) | 0.63 (0.36, 1.12) | -1.78 (-4.42, 0.87) |
| Yes | 16 (3%) | 25 (4%) |  |  |
| Missing | 5 | 1 |  |  |
| **Time from randomisation to start of treatment for a diagnosed emotional disorder (weeks)** |  |  | HR 0.59 (0.31, 1.10) | |
| Mean[sd] | 31.1 [14.3] | 27.8 [17.2] |  |  |
| Median [25^th^, 75^th^ centile] | 32 [20, 45] | 31 [12, 44] |  |  |
| Min, max | 4, 48 | 1, 51 |  |  |
| n | 16 | 25 |  |  |
| **Any treatment offered within 12 months** |  |  |  |  |
| No | 356 (58%) | 371 (61%) | 1.05 (0.93, 1.18) | 2.34 (-3.07, 7.74) |
| Yes | 254 (42%) | 238 (39%) |  |  |
| Missing | 5 | 1 |  |  |
| **Time from randomisation to the decision to offer any treatment (weeks)** |  |  | HR 1.05 (0.88, 1.26) | |
| Mean[sd] | 19.3 [14.9] | 17.1 [13.7] |  |  |
| Median [25^th^, 75^th^ centile] | 16.5 [6, 32] | 13.5 [6, 25] |  |  |
| Min, max | 0, 52 | 0, 52 |  |  |
| N | 254 | 238 |  |  |
| **Any treatment started within 12 months** |  |  |  |  |
| No | 446 (73%) | 440 (72%) | 0.97 (0.81, 1.16) | -0.75 (-5.70, 4.20) |
| Yes | 164 (27%) | 169 (28%) |  |  |
| Missing | 5 | 1 |  |  |
| **Time from randomisation to start of any treatment (weeks)** |  |  | HR 0.95 (0.77, 1.18) | |
| Mean[sd] | 22.1 [14.4] | 20.4 [13.6] |  |  |
| Median [25^th^, 75^th^ centile] | 20 [10, 33.5] | 19 [9, 32] |  |  |
| Min, max | 0, 52 | 0, 49 |  |  |
| N | 164 | 169 |  |  |

Data are n (%) unless otherwise indicated.

*Adjusted by site and other minimisation factors. **Risk ratio unless otherwise specified. ***Hazard ratios (HR) from a Cox model which censors outcomes at 12 months, unless the participants withdraws prior to 12 months. Nelson-Aalen plots can be found in Appendix D. ****Treatment offered includes medications and non-pharmacological interventions, whereas treatment started only includes non-pharmacological interventions as data about start dates were not collected for medications.

Table 2.25: Secondary outcomes – treatment within 18 months

|  | **Intervention Group**  **(n = 615)** | **Control Group**  **(n = 610)** | **Adjusted* risk ratio****  **(95% CI)** | **Adjusted* risk difference**  **(95% CI)** |
| --- | --- | --- | --- | --- |
| **Any treatment offered**** for diagnosed emotional disorder within 18 months** |  |  |  |  |
| No | 565 (93%) | 557 (92%) | 0.89 (0.66, 1.21) | -1.19 (-4.47, 2.10) |
| Yes | 44 (7%) | 49 (8%) |  |  |
| Missing | 6 | 4 |  |  |
| **Time from randomisation to the decision to offer treatment for a diagnosed emotional disorder (weeks)** |  |  | HR*** 0.84 (0.56, 1.27) | |
| Mean[sd] | 35.5 [20.9] | 29.2 [22.3] |  |  |
| Median [25^th^, 75^th^ centile] | 34 [16, 52] | 22 [9, 44] |  |  |
| Min, max | 3, 76 | 1, 77 |  |  |
| n | 44 | 49 |  |  |
| **Any treatment started**** for diagnosed emotional disorder within 18 months** |  |  |  |  |
| No | 586 (96%) | 575 (95%) | 0.73 (0.45, 1.17) | -1.75 (-4.64, 1.15) |
| Yes | 23 (4%) | 31 (5%) |  |  |
| Missing | 6 | 4 |  |  |
| **Time from randomisation to start of treatment for a diagnosed emotional disorder (weeks)** |  |  | HR 0.68 (0.39, 1.17) | |
| Mean[sd] | 40.6 [19.3] | 35.1 [21.7] |  |  |
| Median [25^th^, 75^th^ centile] | 43 [28, 53] | 38 [13, 51] |  |  |
| Min, max | 4, 71 | 1, 75 |  |  |
| n | 23 | 31 |  |  |
| **Any treatment offered within 18 months** |  |  |  |  |
| No | 317 (52%) | 328 (54%) | 1.03 (0.93, 1.15) | 1.73 (-3.62, 7.08) |
| Yes | 292 (48%) | 278 (46%) |  |  |
| Missing | 6 | 4 |  |  |
| **Time from randomisation to the decision to offer any treatment (weeks)** |  |  | HR 1.04 (0.88, 1.23) | |
| Mean[sd] | 25.2 [20.9] | 23.7 [20.8] |  |  |
| Median [25^th^, 75^th^ centile] | 19.5 [8, 38.5] | 17 [7, 37] |  |  |
| Min, max | 0, 78 | 0, 76 |  |  |
| N | 292 | 278 |  |  |
| **Any treatment started within 18 months** |  |  |  |  |
| No | 411 (67%) | 411 (68%) | 1.01 (0.86, 1.19) | 0.39 (-4.76, 5.55) |
| Yes | 198 (33%) | 195 (32%) |  |  |
| Missing | 6 | 4 |  |  |
| **Time from randomisation to start of any treatment (weeks)** |  |  | HR 1.00 (0.82, 1.21) | |
| Mean[sd] | 29.4 [21] | 26.1 [19.4] |  |  |
| Median [25^th^, 75^th^ centile] | 25.5 [12, 44] | 21 [10, 39] |  |  |
| Min, max | 0, 78 | 0, 76 |  |  |
| N | 198 | 195 |  |  |

Data are n (%) unless otherwise indicated.

*Adjusted by site and other minimisation factors. **Risk ratio unless otherwise specified. ***Hazard ratios (HR) from a Cox model which censors outcomes at 18 months, unless the participants withdraws prior to 18 months. Nelson-Aalen plots can be found in Appendix D. ****Treatment offered includes medications and non-pharmacological interventions, whereas treatment started only includes non-pharmacological interventions as data about start dates were not collected for medications.

Table 2.26: Child symptoms and functional impairment (self-reported)

|  | **Baseline**  Mean [sd] | **6 months**  Mean [sd] | **12 months**  Mean [sd] |
| --- | --- | --- | --- |
| Depression symptoms (MFQ) |  |  |  |
| Intervention group | 38.7 [13.8] (n=237) | 36.8 [16] (n=135) | 34.2 [15.9] (n=147) |
| Control Group | 37.7 [13.3] (n=236) | 34.4 [14.9] (n=120) | 33 [16.5] (n=146) |
| Adjusted difference in means*  (95% CI) |  | **2.19 (-1.10, 5.48)** | **-0.06 (-3.51, 3.39)** |
| Anxiety symptoms (RCADS) |  |  |  |
| Intervention group | 55.8 [21.9] (n=237) | 54.2 [23.8] (n=135) | 51.6 [24.3] (n=144) |
| Control Group | 57.7 [20.6] (n=236) | 54.9 [22.2] (n=119) | 52.1 [22.8] (n=145) |
| Adjusted difference in means*  (95% CI) |  | **-2.92 (-7.70, 1.87)** | **-1.80 (-6.52, 2.92)** |
| Separation anxiety disorder (RCADS) |  |  |  |
| Intervention group | 7.7 [4.6] (n=238) | 7.4 [4.7] (n=135) | 7.3 [4.8] (n=144) |
| Control Group | 8.1 [4.5] (n=236) | 7.5 [4.5] (n=119) | 7.2 [4.6] (n=145) |
| Social phobia (RCADS) |  |  |  |
| Intervention group | 17.6 [6.7] (n=237) | 17.8 [6.9] (n=135) | 16.9 [7.2] (n=144) |
| Control Group | 18.5 [6.4] (n=236) | 18.2 [6.1] (n=119) | 17.3 [6.5] (n=145) |
| Generalized anxiety disorder (RCADS) |  |  |  |
| Intervention group | 10.2 [4.7] (n=238) | 9.6 [4.8] (n=135) | 8.9 [4.9] (n=146) |
| Control Group | 10.3 [4.1] (n=236) | 9.5 [4.6] (n=120) | 9 [4.6] (n=145) |
| Panic disorder (RCADS) |  |  |  |
| Intervention group | 12.5 [6.9] (n=238) | 12.1 [7.3] (n=135) | 11.6 [7.2] (n=144) |
| Control Group | 12.8 [7] (n=236) | 13 [6.8] (n=119) | 12.3 [6.6] (n=145) |
| Obsessive compulsive disorder (RCADS) |  |  |  |
| Intervention group | 7.9 [4.5] (n=237) | 7.3 [4.9] (n=135) | 6.9 [4.8] (n=144) |
| Control Group | 8 [4.4] (n=236) | 6.8 [4.5] (n=119) | 6.4 [4.6] (n=145) |
| Low mood (major depressive disorder) (RCADS) |  |  |  |
| Intervention group | 17 [6.7] (n=238) | 16.7 [7.5] (n=135) | 15.9 [7.2] (n=144) |
| Control Group | 16.4 [6.6] (n=236) | 16.5 [6.8] (n=119) | 14.9 [7.3] (n=145) |
| Total Internalising Scale (RCADS) |  |  |  |
| Intervention group | 72.8 [27] (n=237) | 71 [29.6] (n=135) | 67.4 [30.5] (n=144) |
| Control Group | 74.1 [25.5] (n=236) | 71.4 [27.9] (n=119) | 67 [29] (n=145) |
| Emotional symptoms subscale (SDQ) |  |  |  |
| Intervention group | 7.1 [2.4] (n=150) | 7 [2.3] (n=135) | 6.7 [2.5] (n=147) |
| Control Group | 7 [2.2] (n=234) | 7.2 [1.9] (n=125) | 6.8 [2.4] (n=150) |
| Oppositional defiant / conduct disorder subscale (SDQ conduct problems) |  |  |  |
| Intervention group | 3.2 [2.1] (n=150) | 3 [2.1] (n=135) | 3.2 [2.2] (n=147) |
| Control Group | 2.9 [2.1] (n=234) | 2.6 [2] (n=125) | 2.5 [2] (n=150) |
| Adjusted difference in means*  (95% CI) |  | **0.36 (-0.09, 0.81)** | **0.41 (-0.03, 0.84)** |
| Hyperactivity/inattention subscale (SDQ) |  |  |  |
| Intervention group | 6.4 [2.4] (n=150) | 6.4 [2.4] (n=135) | 6.6 [2.6] (n=147) |
| Control Group | 6.3 [2.5] (n=234) | 6.1 [2.4] (n=125) | 6 [2.4] (n=150) |
| Peer relationships problem subscale (SDQ) |  |  |  |
| Intervention group | 3.6 [2.2] (n=150) | 3.7 [2.4] (n=135) | 3.7 [2.1] (n=147) |
| Control Group | 3.8 [2.1] (n=234) | 3.5 [2] (n=125) | 3.6 [2.2] (n=150) |
| Total symptoms (SDQ) |  |  |  |
| Intervention group | 20.3 [5.8] (n=150) | 20.2 [6] (n=135) | 20.2 [6.7] (n=147) |
| Control Group | 19.9 [5.7] (n=234) | 19.4 [5.9] (n=125) | 19 [6.7] (n=150) |
| Prosocial behaviour subscale (SDQ) |  |  |  |
| Intervention group | 6.9 [2.1] (n=150) | 6.9 [2.1] (n=135) | 6.9 [2.1] (n=147) |
| Control Group | 6.7 [2.3] (n=234) | 7 [1.9] (n=125) | 7.4 [1.8] (n=150) |
| Functional Impairment (SDQ impact supplement) |  |  |  |
| Intervention group | 4.9 [2.6] (n=139) | 3.8 [2.8] (n=132) | 3.8 [3.1] (n=147) |
| Control Group | 3.9 [2.5] (n=231) | 3.8 [2.8] (n=122) | 3.7 [2.9] (n=147) |
| Adjusted difference in means*  (95% CI) |  | **-0.03 (-0.66, 0.59)** | **0.07 (-0.57, 0.72)** |

*Adjusted by site and other minimisation factors and score at baseline.

Table 2.27: Child symptoms and functional impairment (parent/carer-reported)

|  | **Baseline**  Mean [sd] | **6 months**  Mean [sd] | **12 months**  Mean [sd] |
| --- | --- | --- | --- |
| Depression symptoms (MFQ) |  |  |  |
| Intervention group | 31.6 [13.3] (n=556) | 25.3 [14.7] (n=404) | 23.5 [15.7] (404) |
| Control Group | 31.5 [14.1] (n=554) | 26.6 [15.4] (n=405) | 23.9 [15.2] (n=404) |
| Adjusted difference in means* (95% CI) |  | **-1.02 (-2.89, 0.86)** | **-0.30 (-2.29, 1.68)** |
| Anxiety symptoms (RCADS) |  |  |  |
| Intervention group | 45.3 [19.7] (n=553) | 41.2 [21.4] (n=393) | 39.3 [22] (n=400) |
| Control Group | 46.6 [20.8] (n=548) | 42.3 [21.6] (n=391) | 39.7 [22.3] (n=403) |
| Adjusted difference in means* (95% CI) |  | **-1.50 (-4.17, 1.18)** | **-0.63 (-3.38, 2.11)** |
| Separation anxiety disorder (RCADS) |  |  |  |
| Intervention group | 8.6 [5.3] (n=557) | 8.1 [5.4] (n=395) | 7.6 [5.4] (n=402) |
| Control Group | 8.9 [5.2] (n=556) | 8.2 [5.1] (n=394) | 7.7 [5.3] (n=403) |
| Social phobia (RCADS) |  |  |  |
| Intervention group | 15.2 [6.6] (n=558) | 14.1 [7] (n=395) | 13.5 [6.9] (n=401) |
| Control Group | 15.4 [6.7] (n=551) | 14.3 [7.1] (n=394) | 13.8 [7.1] (n=403) |
| Generalized anxiety disorder (RCADS) |  |  |  |
| Intervention group | 8.6 [4.3] (n=558) | 7.6 [4.4] (n=398) | 7.1 [4.4] (n=404) |
| Control Group | 8.8 [4.4] (n=554) | 7.8 [4.4] (n=398) | 7.1 [4.4] (n=403) |
| Panic disorder (RCADS) |  |  |  |
| Intervention group | 7.7 [5.5] (n=558) | 6.9 [5.4] (n=394) | 6.8 [5.8] (n=402) |
| Control Group | 8.4 [6.1] (n=550) | 7.4 [5.9] (n=395) | 6.9 [5.7] (n=403) |
| Obsessive compulsive disorder (RCADS) |  |  |  |
| Intervention group | 5.3 [4.1] (n=556) | 4.6 [4.3] (n=395) | 4.3 [4.1] (n=402) |
| Control Group | 5.2 [4] (n=555) | 4.5 [4.1] (n=392) | 4.3 [4.2] (n=403) |
| Low mood (major depressive disorder) (RCADS) |  |  |  |
| Intervention group | 13.2 [6] (n=556) | 11.3 [6.2] (n=395) | 10.7 [6.6] (n=402) |
| Control Group | 12.9 [6.2] (n=552) | 11.5 [6.4] (n=394) | 10.7 [6.5] (n=403) |
| Total Internalising Scale (RCADS) |  |  |  |
| Intervention group | 58.5 [23.9] (n=551) | 52.4 [25.9] (n=393) | 50 [27.2] (n=399) |
| Control Group | 59.5 [25.4] (n=548) | 53.8 [26.5] (n=391) | 50.5 [27.8] (n=403) |
| Emotional symptoms subscale (SDQ) |  |  |  |
| Intervention group | 6.9 [2.4] (n=425) | 6.4 [2.6] (n=410) | 6 [2.7] (n=412) |
| Control Group | 6.5 [2.5] (n=554) | 6.5 [2.6] (n=417) | 6 [2.7] (n=412) |
| Oppositional defiant / conduct disorder subscale (SDQ conduct problems) |  |  |  |
| Intervention group | 3.5 [2.3] (n=425) | 3.3 [2.4] (n=410) | 3.3 [2.4] (n=412) |
| Control Group | 3.5 [2.5] (n=554) | 3.1 [2.4] (n=417) | 3.1 [2.5] (n=412) |
| Adjusted difference in means* (95% CI) |  | **0.09 (-0.21, 0.38)** | **0.09 (-0.21, 0.39)** |
| Hyperactivity/inattention subscale (SDQ) |  |  |  |
| Intervention group | 6.1 [2.6] (n=425) | 5.9 [2.9] (n=410) | 6 [2.8] (n=412) |
| Control Group | 5.9 [2.8] (n=554) | 5.9 [2.8] (n=417) | 5.7 [2.9] (n=412) |
| Peer relationships problem subscale (SDQ) |  |  |  |
| Intervention group | 3.8 [2.3] (n=425) | 4 [2.4] (n=410) | 3.9 [2.5] (n=412) |
| Control Group | 3.9 [2.4] (n=554) | 3.9 [2.4] (n=417) | 3.8 [2.5] (n=412) |
| Total symptoms (SDQ) |  |  |  |
| Intervention group | 20.3 [6.7] (n=425) | 19.6 [7.3] (n=410) | 19.2 [7.7] (n=412) |
| Control Group | 19.8 [7.1] (n=554) | 19.4 [7.5] (n=417) | 18.6 [7.9] (n=412) |
| Prosocial behaviour subscale (SDQ) |  |  |  |
| Intervention group | 6.2 [2.6] (n=425) | 6.3 [2.6] (n=410) | 6.2 [2.6] (n=412) |
| Control Group | 6.3 [2.7] (n=554) | 6.5 [2.5] (n=417) | 6.5 [2.5] (n=412) |
| Functional Impairment (SDQ impact supplement) |  |  |  |
| Intervention group | 5.8 [2.8] (n=424) | 4.7 [3.1] (n=405) | 4.4 [3.2] (n=405) |
| Control Group | 4.9 [2.9] (n=551) | 4.8 [3.1] (n=410) | 4.5 [3.3] (n=406) |
| Adjusted difference in means* (95% CI) |  | **-0.06 (-0.45, 0.33)** | **-0.07 (-0.49, 0.34)** |

*Adjusted by site and other minimisation factors and score at baseline.

Table 2.28: Parent/carer self-reported outcomes

|  | Baseline  Mean [sd] | 6 months  Mean [sd] | 12 months  Mean [sd] |
| --- | --- | --- | --- |
| Depression symptoms (PHQ-9) |  |  |  |
| Intervention group | 9.5 [6.6] (n=557) | 7.9 [6.1] (n=385) | 7.6 [6.2] (n=402) |
| Control Group | 9.1 [6.3] (n=555) | 8.1 [6.2] (n=388) | 7.8 [6.2] (n=401) |
| Adjusted difference in means*  (95% CI) |  | **-0.26 (-1.08, 0.56)** | **-0.22 (-1.04, 0.60)** |
| Anxiety symptoms (GAD-7) |  |  |  |
| Intervention group | 8.7 [6] (n=559) | 7 [5.6] (n=383) | 6.7 [5.5] (n=400) |
| Control Group | 8.3 [5.9] (n=556) | 7.2 [5.6] (n=386) | 6.7 [5.6] (n=401) |
| Adjusted difference in means*  (95% CI) |  | **-0.16 (-0.92, 0.59)** | **0.01 (-0.73, 0.75)** |

*Adjusted by site and other minimisation factors and score at baseline.

Table 2.29: Secondary outcomes for the child – other*

|  | **6 months** | | **12 months** | |
| --- | --- | --- | --- | --- |
|  | **Intervention Group**  **(n = 615)** | **Control Group**  **(n = 610)** | **Intervention Group**  **(n = 615)** | **Control Group**  **(n = 610)** |
|  |  |  |  |  |
| Questionnaire returned by parent/carer or young person | 483 (79%) | 495 (81%) | 478 (78%) | 482 (79%) |
| Any time out of education, employment or training (in last 3 months) due to emotional difficulties |  |  |  |  |
| Yes | 130 (39%) | 133 (41%) | 158 (45%) | 153 (46%) |
| No | 201 (61%) | 190 (59%) | 194 (55%) | 180 (54%) |
| Missing | 152 | 172 | 126 | 149 |
| Time out of education, employment or training (in previous 3 months) due to emotional difficulties (days) |  |  |  |  |
| Mean[sd] | 3.5 [9] | 4.2 [12.5] | 4.5 [11.4] | 3.7 [9.1] |
| Median [25^th^, 75^th^ centile] | 0 [0, 3] | 0 [0, 3] | 0 [0, 3] | 0 [0, 3] |
| Min, max | 0, 65 | 0, 100** | 0, 90 | 0, 63 |
| n | 331 | 323 | 352 | 333 |

*Note where responses are discrepant between child and parent, the child report is given precedence **Participant reported data so could not be queried

### Sensitivity analysis for participant reported outcomes

Table 2.30: Imputation of Child symptoms and functional impairment (self-reported)

|  | **6 months**  Mean [sd] | **12 months**  Mean [sd] |
| --- | --- | --- |
| Depression symptoms (MFQ) |  |  |
| Adjusted difference in means* (95% CI) |  |  |
| Observed data, n=483 | 2.19 (-1.10, 5.48) | -0.06 (-3.51, 3.39) |
| Imputed data, n=502 | 1.30 (-1.60, 4.21) | 0.44 (-2.70, 3.58) |
| Anxiety symptoms (RCADS) |  |  |
| Adjusted difference in means* (95% CI) |  |  |
| Observed data, n=483 | -2.92 (-7.70, 1.87) | -1.80 (-6.52, 2.92) |
| Imputed data, n=502 | -2.16 (-6.54, 2.22) | -1.61 (-5.88, 2.66) |
| Comorbid oppositional defiant / conduct disorder symptoms (SDQ conduct problems) |  |  |
| Adjusted difference in means* (95% CI) |  |  |
| Observed data, n=446 | 0.36 (-0.09, 0.81) | 0.41 (-0.03, 0.84) |
| Imputed data, n=502 | 0.29 (-0.12, 0.71) | 0.30 (-0.10, 0.69) |
| Functional Impairment (SDQ impact supplement) |  |  |
| Adjusted difference in means* (95% CI) |  |  |
| Observed data, n=438 | -0.03 (-0.66, 0.59) | 0.07 (-0.57, 0.72) |
| Imputed data, n=502 | 0.14 (-0.42, 0.70) | 0.13 (-0.49, 0.76) |

*Adjusted by site and other minimisation factors and score at baseline.

Table 2.31: Imputation of Child symptoms and functional impairment (parent/carer-reported)

|  | **6 months**  Mean [sd] | **12 months**  Mean [sd] |
| --- | --- | --- |
| Depression symptoms (MFQ) |  |  |
| Adjusted difference in means* (95% CI) |  |  |
| Observed data, n= 1116 | -1.02 (-2.89, 0.86) | -0.30 (-2.29, 1.68) |
| Imputed data, n= 1121 | -0.67 (-2.52, 1.17) | -0.17 (-2.12, 1.78) |
| Anxiety symptoms (RCADS) |  |  |
| Adjusted difference in means* (95% CI) |  |  |
| Observed data, n= 1116 | -1.50 (-4.17, 1.18) | -0.63 (-3.38, 2.11) |
| Imputed data, n= 1121 | -1.23 (-3.93, 1.48) | -0.73 (-3.60, 2.14) |
| Comorbid oppositional defiant / conduct disorder symptoms (SDQ conduct problems) |  |  |
| Adjusted difference in means* (95% CI) |  |  |
| Observed data, n= 1077 | 0.09 (-0.21, 0.38) | 0.09 (-0.21, 0.39) |
| Imputed data, n=1121 | 0.08 (-0.22, 0.37) | 0.06 (-0.24, 0.36) |
| Functional Impairment (SDQ impact supplement) |  |  |
| Adjusted difference in means* (95% CI) |  |  |
| Observed data, n= 1077 | -0.06 (-0.45, 0.33) | -0.07 (-0.49, 0.34) |
| Imputed data, n= 1121 | 0.09 (-0.29, 0.47) | 0.06 (-0.33, 0.44) |

*Adjusted by site and other minimisation factors and score at baseline.

Table 2.32: Imputation of Parent/carer self-reported outcomes

|  | **6 months**  Mean [sd] | **12 months**  Mean [sd] |
| --- | --- | --- |
| Depression symptoms (PHQ-9) |  |  |
| Adjusted difference in means* (95% CI) |  |  |
| Observed data, n= 1117 | -0.26 (-1.08, 0.56) | -0.22 (-1.04, 0.60) |
| Imputed data, n= 1121 | -0.13 (-0.93, 0.66) | -0.12 (-0.91, 0.66) |
| Anxiety symptoms (GAD-7) |  |  |
| Adjusted difference in means* (95% CI) |  |  |
| Observed data, n= 1119 | -0.16 (-0.92, 0.59) | 0.01 (-0.73, 0.75) |
| Imputed data, n=1121 | -0.02 (-0.74, 0.70) | 0.09 (-0.64, 0.81) |

*Adjusted by site and other minimisation factors and score at baseline.

### Additional data collection

Table 2.33: Post-traumatic stress disorder symptoms in the child/young person (self-reported)

|  | **Baseline**  Mean [sd] | **6 months**  Mean [sd] | **12 months**  Mean [sd] |
| --- | --- | --- | --- |
| PTSD symptoms (CRIES-8) |  |  |  |
| Intrusion subscale | 11.7 [6.3] (n = 247) | 10.6 [6.5] (n = 215) | 10.7 [6.6] (n = 278) |
| Avoidance subscale | 12.3 [6.4] (n = 247) | 11.2 [6.5] (n = 215) | 11.4 [6.6] (n = 278) |
| Total score | 24.0 [12.0] (n = 247) | 21.9 [12.2] (n = 215) | 22.0 [12.6] (n = 278) |

Formal analyses investigating the effects of the pandemic on CRIES will be reported separately.

Table 2.34: Post-traumatic stress disorder symptoms in the child/young person (parent/carer-reported)

|  | **Baseline**  Mean [sd] | **6 months**  Mean [sd] | **12 months**  Mean [sd] |
| --- | --- | --- | --- |
| PTSD symptoms (CRIES-8) |  |  |  |
| Intrusion subscale | 8.6 [6.8] (n = 497) | 7.1 [6.8] (n = 575) | 6.8 [6.5] (n = 738) |
| Avoidance subscale | 7.5 [6.3] (n = 497) | 6.7 [6.3] (n = 576) | 6.6 [6.3] (n = 738) |
| Total score | 16.1 [12.2] (n = 497) | 13.8 [12.3] (n = 575) | 13.4 [12.0] (n = 738) |

Formal analyses investigating the effects of the pandemic on the CRIES-8 will be reported separately.

### Safety

Table 2.35: Summary of child safety outcomes (self-report)

|  | **6 months** | | **12 months** | |
| --- | --- | --- | --- | --- |
|  | **Intervention Group**  **(n = 252)** | **Control Group**  **(n = 250)** | **Intervention Group**  **(n = 252)** | **Control Group**  **(n = 250)** |
| Questionnaire returned* | 140 (56%) | 140 (56%) | 157 (62%) | 156 (62%) |
| A significant deterioration in depression (compared to baseline)** (MFQ) |  |  |  |  |
| No | 100 (77%) | 89 (76%) | 109 (77%) | 108 (75%) |
| Yes | 30 (23%) | 28 (24%) | 33 (23%) | 36 (25%) |
| Missing | 10 | 23 | 15 | 12 |
| Have you thought about hurting yourself, even if you would not do it, in the last 6 months? |  |  |  |  |
| Not at all | 42 (32%) | 35 (30%) | 48 (34%) | 48 (34%) |
| Once or twice | 33 (25%) | 27 (23%) | 35 (25%) | 35 (24%) |
| Three or more times | 58 (44%) | 56 (47%) | 59 (42%) | 60 (42%) |
| Missing | 7 | 22 | 15 | 13 |
| Have you hurt yourself on purpose in anyway in the last 6 months? |  |  |  |  |
| Not at all | 79 (59%) | 74 (64%) | 84 (59%) | 94 (66%) |
| Once | 18 (14%) | 13 (11%) | 22 (15%) | 16 (11%) |
| Two or more times | 36 (27%) | 28 (24%) | 36 (25%) | 33 (23%) |
| Missing | 7 | 25 | 15 | 13 |

*The denominator for 6 months and 12 months are participants who have started the 6 month or 12 month questionnaire, respectively. **Defined as a score indicative of depression (27 or above) on the Mood and Feelings Questionnaire (MFQ) completed at follow-up, where this represents a deterioration from baseline of 5 points or more

Table 2.36: Summary of child safety outcomes (parent/carer reported)

|  | **6 months** | | **12 months** | |
| --- | --- | --- | --- | --- |
|  | **Intervention Group**  **(n = 560)** | **Control Group**  **(n = 561)** | **Intervention Group**  **(n = 560)** | **Control Group**  **(n = 561)** |
| Questionnaire returned* | 434 (78%) | 440 (78%) | 431 (77%) | 434 (77%) |
| A significant deterioration in depression (compared to baseline)** (MFQ) |  |  |  |  |
| Yes | 359 (89%) | 339 (84%) | 346 (86%) | 349 (87%) |
| No | 43 (11%) | 63 (16%) | 56 (14%) | 52 (13%) |
| Missing | 32 | 38 | 29 | 33 |

*The denominator for 6 months and 12 months are parents who have started the 6 month or 12 month questionnaire, respectively. **Defined as a score indicative of depression (27 or above) on the Mood and Feelings Questionnaire (MFQ) completed at follow-up, where this represents a deterioration from baseline of 5 points or more

Table 2.37: Summary of child safety outcomes (reported by either child or parent/carer)**

|  | **6 months** | | **12 months** | |
| --- | --- | --- | --- | --- |
|  | **Intervention Group**  **(n = 615)** | **Control Group**  **(n = 610)** | **Intervention Group**  **(n = 615)** | **Control Group**  **(n = 610)** |
| Questionnaire returned* | 483 (79%) | 495 (81%) | 478 (78%) | 482 (79%) |
| Any overnight hospital admissions due to emotional difficulties in the previous 3 months? |  |  |  |  |
| No | 402 (98%) | 398 (99%) | 407 (99%) | 423 (100%) |
| Yes | 7 (2%) | 6 (1%) | 6 (1%) | 1 (0%) |
| Missing | 74 | 91 | 65 | 58 |
| Any visits to A&E due to emotional difficulties in the previous 3 months? |  |  |  |  |
| No | 392 (95%) | 391 (93%) | 402 (94%) | 425 (97%) |
| Yes | 20 (5%) | 29 (7%) | 25 (6%) | 12 (3%) |
| Missing | 71 | 75 | 51 | 45 |

*The denominator for 6 months and 12 months are whether the child or parent started the 6 month or 12 month questionnaire, respectively. ** Note, hospital admissions or A&E visits defined as either parent or child has reporting a visit

Table 2.38: Summary of parent/carer self-reported safety outcomes

|  | **6 months** | | **12 months** | |
| --- | --- | --- | --- | --- |
|  | **Intervention Group**  **(n = 560)** | **Control Group**  **(n = 561)** | **Intervention Group**  **(n = 560)** | **Control Group**  **(n = 561)** |
| Questionnaire returned* | 434 (78%) | 440 (78%) | 431 (77%) | 434 (77%) |
| A significant deterioration in depression compared to baseline** (PHQ-9) |  |  |  |  |
| No | 365 (95%) | 354 (92%) | 373 (93%) | 371 (93%) |
| Yes | 19 (5%) | 32 (8%) | 27 (7%) | 28 (7%) |
| Missing | 50 | 54 | 31 | 35 |

*The denominator for 6 months and 12 months are parents who have started the 6 month or 12 month questionnaire, respectively. **Defined as a score indicative of depression (15 or above) on the Patient Health Questionnaire (PHQ-9) completed at follow-up, where this represents a deterioration from baseline of 5 points or more.

Table 2.39: Summary of safety outcomes from records within 12 months

|  | **Intervention Group**  **(n = 615)** | **Control Group**  **(n = 610)** |
| --- | --- | --- |
| Death of child within 12 months |  |  |
| No | 610 (100%) | 609 (100%) |
| Yes | 0 (0%) | 0 (0%) |
| Missing* | 5 | 1 |

*Participants are categorised as missing if they withdrew consent to access records prior to 12 months.

Table 2.40: Summary of safety outcomes from records within 18 months*

|  | **Intervention Group**  **(n = 615)** | **Control Group**  **(n = 610)** |
| --- | --- | --- |
| Death of child within 18 months |  |  |
| No | 609 (100%) | 606 (100%) |
| Yes | 0 (0%) | 0 (0%) |
| Missing | 6 | 4 |

*Participants are categorised as missing if they withdrew consent to access records prior to 18 months.

**Table 2.41: Withdrawal summary**

|  | **Intervention Group**  **(n = 615)** | **Control Group**  **(n = 610)** |
| --- | --- | --- |
| **Withdrawn** |  |  |
| No | 569 (93%) | 579 (95%) |
| Yes | 46 (7%) | 31 (5%) |
| **Withdrawn from completing questionnaires** |  |  |
| No | 569 (93%) | 579 (95%) |
| Yes | 46 (7%) | 31 (5%) |
| **Withdrawn consent to access records** |  |  |
| No | 609 (99%) | 606 (99%) |
| Yes | 6 (1%) | 4 (1%) |
| **If yes, timepoint of withdrawing consent to access records** |  |  |
| Before 12 months | 5 (1%) | 1 (<1%) |
| After 12 months | 1 (<1%) | 3 (<1%) |

All data are N (%) unless otherwise indicated. Primary participants only are included in this summary.

Participants who have withdrawn at any point are included in this table.

## Final Analysis Appendix: Adherence to intervention

Table 2.42: Adherence to intervention (self-reported DAWBA – 11-15)

|  | **Intervention Group** |
| --- | --- |
|  | **(n =233)** |
| **DAWBA completion** |  |
| Fully | 91 (39%) |
| Partially | 4 (2%) |
| Not at all | 138 (59%) |
| n | 233 |
| **Reason if DAWBA not fully completed** |  |
| Time elapsed | 47 (33%) |
| Withdrew consent | 2 (1%) |
| Declined | 17 (12%) |
| Unable to contact | 11 (8%) |
| Unknown | 46 (32%) |
| Other | 19 (13%) |
| **Time from randomisation to DAWBA completion (days)** |  |
| Mean[sd] | 4.6 [3.4] |
| Median [25^th^, 75^th^ centile] | 4 [2, 6] |
| Min, max | 0, 17 |
| n | 95 |
| **Method of DAWBA completion** |  |
| Online | 94 (99%) |
| Telephone | 0 (0%) |
| Both | 1 (1%) |
| Missing | 95 |
| **Number of DAWBA modules completed (out of 13)** |  |
| Mean [sd] | 12.9 [1] |
| Median [25^th^, 75^th^ centile] | 13 [13, 13] |
| Min, max | 3, 13 |
| n | 95 |

All data are N (%) unless otherwise indicated.

Table 2.43: Adherence to intervention (self-reported DAWBA – 16-17)

|  | **Intervention Group** |
| --- | --- |
|  | **(n = 79)** |
| **DAWBA completion** |  |
| Fully | 60 (76%) |
| Partially | 3 (4%) |
| Not at all | 16 (20%) |
| n | 79 |
| **Reason if DAWBA not fully completed** |  |
| Time elapsed | 6 (32%) |
| Declined | 2 (11%) |
| Unable to contact | 2 (11%) |
| Unknown | 9 (47%) |
| **Time from randomisation to DAWBA completion (days)** |  |
| Mean[sd] | 2.8 [3.1] |
| Median [25^th^, 75^th^ centile] | 2 [0, 5] |
| Min, max | 0, 12 |
| n | 63 |
| **Method of DAWBA completion** |  |
| Online | 63 (100%) |
| Telephone | 0 (0%) |
| Both | 0 (0%) |
| **Number of DAWBA modules completed (out of 13)** |  |
| Mean [sd] | 11.9 [1.5] |
| Median [25^th^, 75^th^ centile] | 12 [12, 12] |
| Min, max | 3, 13 |
| n | 63 |

All data are N (%) unless otherwise indicated.

Table 2.44: Adherence to intervention (parent/carer-reported DAWBA)

|  | **Intervention Group** |
| --- | --- |
|  | **(n = 569)** |
| **DAWBA completion** |  |
| Fully | 421 (74%) |
| Partially | 23 (4%) |
| Not at all | 125 (22%) |
| n | 569 |
| **Reason if DAWBA not fully completed** |  |
| Time elapsed | 71 (48%) |
| Withdrew consent | 2 (1%) |
| Declined | 2 (1%) |
| Unable to contact | 19 (13%) |
| Unknown | 33 (22%) |
| Other | 21 (14%) |
| **Time from randomisation to DAWBA completion (days)** |  |
| Mean[sd] | 3.1 [3.6] |
| Median [25^th^, 75^th^ centile] | 2 [0, 5] |
| Min, max | 0, 34 |
| n | 444 |
| **Method of DAWBA completion** |  |
| Online | 440 (99%) |
| Telephone | 4 (1%) |
| Both | 0 (0%) |
| **Number of DAWBA modules completed (out of 13)** |  |
| Mean [sd] | 12.6 [1.7] |
| Median [25^th^, 75^th^ centile] | 13 [13, 13] |
| Min, max | 3, 13 |
| n | 444 |

All data are N (%) unless otherwise indicated.

## Final Analysis Appendix: Diagnoses

Table 2.45: Secondary outcomes – diagnoses of emotional disorders from records within 12 and 18 months

|  | **12 Months** | | **18 Months*** | |
| --- | --- | --- | --- | --- |
|  | **Intervention Group**  **(n = 615)** | **Control Group**  **(n = 610)** | **Intervention Group**  **(n = 615)** | **Control Group**  **(n = 610)** |
| **Diagnoses** |  |  |  |  |
| *Acute stress reaction* | 1 (<1%) | 0 (0%) | 1 (<1%) | 0 (0%) |
| *Adjustment disorder* | 1 (<1%) | 1 (<1%) | 1 (<1%) | 2 (<1%) |
| *Agoraphobia* | 0 (0%) | 0 (0%) | 0 (0%) | 1 (<1%) |
| *Anxiety disorder* | 4 (<1%) | 1(<1%) | 6 (1%) | 3 (<1%) |
| *Body dysmorphic disorder (bdd)* | 1 (0%) | 0 (<1%) | 2 (<1%) | 0 (0%) |
| *Childhood emotional disorder unspecified (f93.9)* | 4 (1%) | 5 (<1%) | 4 (1%) | 5 (1%) |
| *Depression* | 10 (1%) | 8 (2%) | 17 (3%) | 14 (2%) |
| *Depressive disorder* | 0 (0%) | 0 (0%) | 0 (0%) | 1 (<1%) |
| *Depressive episode (mild)* | 0 (<1%) | 1 (0%) | 0 (0%) | 1 (<1%) |
| *Depressive episode (moderate)* | 2 (0%) | 4 (1%) | 5 (1%) | 4 (1%) |
| *Depressive episode (severe)* | 0 (0%) | 1 (<1%) | 1 (<1%) | 1 (<1%) |
| *Depressive episode (severity not specified)* | 1 (<1%) | 2 (<1%) | 1 (<1%) | 2 (<1%) |
| *Generalized anxiety disorder (gad)* | 15 (3%) | 12 (2%) | 19 (3%) | 22 (4%) |
| *Manic episode* | 0 (0%) | 0 (0%) | 1 (<1%) | 0 (0%) |
| *Mixed anxiety and depressive disorder* | 5 (<1%) | 7 (1%) | 9 (1%) | 9 (1%) |
| *Obsessive compulsive disorder (ocd)* | 16 (2%) | 13 (3%) | 27 (4%) | 21 (3%) |
| *Trichotillomania* | 0 (0%) | 3 (<1%) | 0 (0%) | 5 (1%) |
| *Other/unspecified mood (affective) disorder* | 1 (0%) | 0 (<1%) | 1 (<1%) | 0 (0%) |
| *Other anxiety disorder* | 5 (<1%) | 5 (<1%) | 6 (1%) | 6 (1%) |
| *Other depressive episode* | 0 (0%) | 0 (0%) | 1 (<1%) | 0 (0%) |
| *Panic disorder* | 1 (<1%) | 5 (<1%) | 4 (1%) | 7 (1%) |
| *Persistent depressive disorder* | 0 (0%) | 0 (0%) | 0 (0%) | 1 (<1%) |
| *Post-traumatic stress disorder (ptsd)* | 4 (<1%) | 3 (<1%) | 6 (1%) | 4 (1%) |
| *Recurrent depressive disorder (moderate)* | 0 (0%) | 1 (<1%) | 0 (0%) | 1 (<1%) |
| *Selective mutism* | 1 (<1%) | 0 (0%) | 1 (<1%) | 0 (0%) |
| *Separation anxiety disorder* | 2 (<1%) | 2 (<1%) | 3 (<1%) | 2 (<1%) |
| *Social anxiety disorder* | 6 (<1%) | 5 (1%) | 7 (1%) | 7 (1%) |
| *Social phobia* | 2 (<1%) | 0 (<1%) | 4 (1%) | 0 (0%) |
| *Specific phobia (any)* | 4 (<1%) | 7 (1%) | 6 (1%) | 10 (2%) |
|  |  |  |  |  |

Data are n (%) unless otherwise indicated.

*18-month column includes all diagnoses between randomisation and 18 months. Child / young person could receive more than one emotional disorder diagnosis.

Table 2.46: Secondary outcomes – child/young person and parent/carer reported diagnoses within 12 months

|  | **Child/young person reported** | | **Parent/carer reported** | |
| --- | --- | --- | --- | --- |
|  | **Intervention Group**  **(n = 615)** | **Control Group**  **(n = 610)** | **Intervention Group**  **(n = 615)** | **Control Group**  **(n = 610)** |
| **Reported diagnoses within 12 months** |  |  |  |  |
| *Anxiety disorder* | 0 (0%) | 1 (<1%) | 1 (<1%) | 1 (<1%) |
| *Depression* | 13 (2%) | 14 (2%) | 12 (2%) | 11 (2%) |
| *Generalized anxiety disorder* | 5 (1%) | 5 (1%) | 2 (<1%) | 8 (1%) |
| *Mixed anxiety and depressive disorder* | 0 (0%) | 1 (<1%) | 0 (0%) | 0 (0%) |
| *Obsessive compulsive disorder* | 4 (1%) | 4 (1%) | 7 (1%) | 14 (2%) |
| *Panic disorder* | 5 (1%) | 1 (<1%) | 3 (<1%) | 0 (0%) |
| *Persistent depressive disorder* | 0 (0%) | 0 (0%) | 0 (0%) | 1 (<1%) |
| *Post-traumatic stress disorder* | 2 (<1%) | 1 (<1%) | 3 (<1%) | 0 (0%) |
| *Separation anxiety disorder* | 0 (0%) | 0 (0%) | 2 (<1%) | 0 (0%) |
| *Social phobia* | 1 (<1%) | 0 (0%) | 1 (<1%) | 0 (0%) |
| *Specific phobia (any)* | 1 (<1%) | 0 (0%) | 1 (<1%) | 2 (<1%) |
| *Trichotillomania* | 0 (0%) | 0 (0%) | 0 (0%) | 1 (<1%) |

Data are n (%) unless otherwise indicated.

## Final Analysis Appendix: Interventions

Table 2.47: Secondary outcomes – Treatments offered for a diagnosed emotional disorder within 12 and 18 months

|  | **12 Months** | | **18 Months*** | |
| --- | --- | --- | --- | --- |
|  | **Intervention Group**  **(n = 615)** | **Control Group**  **(n = 610)** | **Intervention Group**  **(n = 615)** | **Control Group**  **(n = 610)** |
| **Treatments/Interventions**, ***** |  |  |  |  |
| Advise/encourage parent/carer to access own psychological support | 1 | 0 | 1 | 0 |
| *Art therapy* | 1 | 0 | 1 | 0 |
| *Brief psychological intervention* | 1 | 5 | 1 | 6 |
| *Cognitive behavioural therapy* | 14 | 18 | 19 | 26 |
| *Dialectical behaviour therapy* | 1 | 0 | 1 | 0 |
| *Drama therapy* | 0 | 1 | 0 | 1 |
| *Family therapy* | 1 | 2 | 1 | 2 |
| *Group primarily for the child/young person* | 2 | 2 | 3 | 2 |
| *Group primarily for the parent/carer* | 1 | 1 | 1 | 1 |
| *Iapt interventions (if not included in other categories)* | 1 | 2 | 2 | 3 |
| *Individual session with parent/carer alongside specific psychological therapy for the child/young person* | 0 | 1 | 0 | 1 |
| *Online/computerised cbt without therapist support* | 1 | 0 | 1 | 0 |
| *Psycho-education* | 3 | 2 | 3 | 2 |
| *Psychodynamic / psychoanalytic therapy* | 0 | 1 | 1 | 1 |
| *Resources recommended* | 10 | 6 | 15 | 8 |
| *Support and information for parents and family* | 0 | 3 | 0 | 3 |
| *Therapist-supported online/computerised intervention* | 0 | 1 | 0 | 1 |
|  |  |  |  |  |

Data are n (%) unless otherwise indicated.

*18 month column includes all treatments offered between randomisation and 18 months **Numbers within rows show the number of participants who were offered each of the treatments/interventions. Participants can have more than one treatment/intervention so may appear in more than one row. ***Please note, all children/young people accepted into CAMHS are offered active case management/care coordination as part of routine care, however, active case management/care coordination was underreported during data collection. The numbers of CYP offered active case management/care coordination for a diagnosed emotional disorder within 12 months in the intervention group and control group respectively were 4 and 9 and the numbers within 18 months were 4 and 11.

Table 2.48: Secondary outcomes – Treatments/interventions started for a diagnosed emotional disorder within 12 and 18 months

|  | **12 Months** | | **18 Months*** | |
| --- | --- | --- | --- | --- |
|  | **Intervention Group**  **(n = 615)** | **Control Group**  **(n = 610)** | **Intervention Group**  **(n = 615)** | **Control Group**  **(n = 610)** |
| **Treatments**,***** |  |  |  |  |
| *Art therapy* | 1 | 0 | 1 | 0 |
| *Brief psychological intervention* | 2 | 1 | 2 | 3 |
| *Cognitive behavioural therapy* | 6 | 11 | 10 | 17 |
| *Dialectical behaviour therapy* | 1 | 0 | 1 | 0 |
| *Drama therapy* | 0 | 1 | 0 | 1 |
| *Group primarily for the child/young person* | 1 | 2 | 2 | 2 |
| *Group primarily for the parent/carer* | 0 | 1 | 0 | 1 |
| *Iapt interventions (if not included in other categories)* | 0 | 2 | 1 | 2 |
| *Individual session with parent/carer alongside specific psychological therapy for the child/young person* | 0 | 1 | 0 | 1 |
| *Online/computerised cbt without therapist support* | 1 | 0 | 1 | 0 |
| *Psycho-education* | 3 | 2 | 4 | 2 |
| *Psychodynamic / psychoanalytic therapy* | 0 | 1 | 0 | 1 |
| *Resources recommended* | 2 | 1 | 2 | 1 |
| *Support and information for parents and family* | 0 | 1 | 0 | 1 |
| *Therapist-supported online/computerised intervention* | 0 | 1 | 0 | 1 |
|  |  |  |  |  |

Data are n (%) unless otherwise indicated.*18 month column includes all treatments/interventions started between randomisation and 18 months **Numbers within rows show the number of participants who started each of the treatments/interventions. Participants can have more than one treatment/intervention so may appear in more than one row. ***Please note, all children/young people accepted into CAMHS will have started active case management/care coordination as part of routine care, however, active case management/care coordination was underreported during data collection. The numbers of CYP starting active case management/care coordination for a diagnosed emotional disorder within 12 months in the intervention group and control group respectively were 4 and 8 and the numbers within 18 months were 4 and 11.

Table 2.49: Secondary outcomes – Any treatments/interventions offered within 12 and 18 months

|  | **12 Months** | | **18 Months*** | |
| --- | --- | --- | --- | --- |
|  | **Intervention Group**  **(n = 615)** | **Control Group**  **(n = 610)** | **Intervention Group**  **(n = 615)** | **Control Group**  **(n = 610)** |
| **Treatments/interventions**, ***** |  |  |  |  |
| Advise/encourage parent/carer to access own psychological support | 3 | 3 | 5 | 4 |
| Applied relaxation | 1 | 2 | 1 | 2 |
| *Art therapy* | 3 | 0 | 4 | 0 |
| *Attachment based interventions* | 0 | 0 | 1 | 0 |
| *Brief psychological intervention* | 14 | 10 | 20 | 16 |
| *Cognitive behavioural therapy* | 61 | 58 | 74 | 74 |
| *Crisis support from specialist teams* | 9 | 4 | 14 | 6 |
| *Day patient admission (mental health unit)* | 1 | 0 | 1 | 0 |
| *Dialectical behaviour therapy* | 4 | 3 | 5 | 5 |
| *Drama therapy* | 1 | 2 | 1 | 2 |
| *Eye movement desensitization and reprocessing* | 0 | 0 | 1 | 0 |
| *Family therapy* | 13 | 9 | 16 | 12 |
| *Group for both the young person and parent/carer* | 0 | 2 | 0 | 2 |
| *Group primarily for the child/young person* | 10 | 8 | 15 | 11 |
| *Group primarily for the parent/carer* | 15 | 23 | 21 | 26 |
| *Iapt interventions (if not included in other categories)* | 7 | 6 | 10 | 9 |
| *Individual session with parent/carer alongside specific psychological therapy for the child/young person* | 0 | 6 | 0 | 7 |
| *Inpatient admission (mental health unit)* | 2 | 0 | 2 | 0 |
| *Intervention provided by peer support workers and family support workers* | 2 | 1 | 2 | 2 |
| *Online/computerised cbt without therapist support* | 3 | 0 | 3 | 0 |
| *Online/computerised cbt-based supported self-help* | 0 | 1 | 0 | 1 |
| *Other online/computerised intervention without therapist support* | 3 | 3 | 3 | 3 |
| *Paediatric ward admission (for mental health reason)* | 3 | 1 | 4 | 1 |
| *Play therapy* | 0 | 1 | 0 | 2 |
| *Psycho-education* | 28 | 30 | 34 | 36 |
| *Psychodynamic / psychoanalytic therapy* | 3 | 2 | 3 | 2 |
| *Resources recommended* | 86 | 75 | 106 | 98 |
| *Support and information for parents and family* | 32 | 33 | 39 | 43 |
| *Therapist-supported online/computerised intervention* | 3 | 5 | 4 | 8 |
|  |  |  |  |  |
|  |  |  |  |  |

Data are n (%) unless otherwise indicated.

*18 month column includes all treatments/interventions offered between randomisation and 18 months **Numbers within rows show the number of participants who were offered each of the treatments/interventions. Participants can have more than one treatment/intervention so may appear in more than one row. ***Please note, all children/young people accepted into CAMHS are offered active case management/care coordination as part of routine care, however, active case management/care coordination was underreported during data collection. The numbers of CYP offered active case management within 12 months in the intervention group and control group respectively were 90 and 78 and the numbers within 18 months were 113 and 105.

Table 2.50: Secondary outcomes – Any treatments/interventions started within 12 and 18 months

|  | **12 Months** | | **18 Months*** | |
| --- | --- | --- | --- | --- |
|  | **Intervention Group**  **(n = 615)** | **Control Group**  **(n = 610)** | **Intervention Group**  **(n = 615)** | **Control Group**  **(n = 610)** |
| **Treatments/interventions**,***** |  |  |  |  |
| Applied relaxation | 1 | 2 | 1 | 2 |
| *Art therapy* | 2 | 0 | 2 | 0 |
| *Brief psychological intervention* | 13 | 5 | 19 | 11 |
| *Cognitive behavioural therapy* | 34 | 45 | 52 | 53 |
| *Crisis support from specialist teams* | 9 | 4 | 14 | 6 |
| *Day patient admission (mental health unit)* | 1 | 0 | 1 | 0 |
| *Dialectical behaviour therapy* | 3 | 1 | 4 | 3 |
| *Drama therapy* | 2 | 1 | 2 | 2 |
| *Eye movement desensitization and reprocessing* | 0 | 1 | 0 | 1 |
| *Family therapy* | 9 | 5 | 10 | 6 |
| *Group for both the young person and parent/carer* | 1 | 1 | 1 | 2 |
| *Group primarily for the child/young person* | 5 | 4 | 8 | 6 |
| *Group primarily for the parent/carer* | 7 | 16 | 8 | 18 |
| *Iapt interventions (if not included in other categories)* | 1 | 5 | 3 | 6 |
| *Individual session with parent/carer alongside specific psychological therapy for the child/young person* | 0 | 3 | 0 | 6 |
| *Inpatient admission (mental health unit)* | 1 | 0 | 1 | 0 |
| *Intervention provided by peer support workers and family support workers* | 1 | 0 | 1 | 1 |
| *Online/computerised cbt without therapist support* | 1 | 0 | 1 | 0 |
| *Online/computerised cbt-based supported self-help* | 0 | 1 | 0 | 1 |
| *Other online/computerised intervention without therapist support* | 2 | 1 | 2 | 1 |
| *Paediatric ward admission (for mental health reason)* | 4 | 0 | 5 | 0 |
| *Play therapy* | 0 | 1 | 0 | 2 |
| *Psycho-education* | 25 | 25 | 32 | 28 |
| *Psychodynamic / psychoanalytic therapy* | 3 | 2 | 3 | 2 |
| *Resources recommended* | 24 | 19 | 30 | 23 |
| *Support and information for parents and family* | 17 | 17 | 21 | 21 |
| *Therapist-supported online/computerised intervention* | 2 | 2 | 2 | 3 |

Data are n (%) unless otherwise indicated.*18 month column includes all treatments/interventions started between randomisation and 18 months **Numbers within rows show the number of participants who were offered each of the treatments/interventions. Participants can have more than one treatment/intervention so may appear in more than one row. ***Please note, all children/young people accepted into CAMHS start active case management/care coordination as part of routine care, however, active case management/care coordination was underreported during data collection. The numbers of CYP starting active case management/care coordination within 12 months in the intervention group and control group respectively were 84 and 68 and the numbers within 18 months were 101 and 92.

Table 2.51: Secondary outcomes – Medications offered* for an emotional disorder within 12 and 18 months

|  | **12 Months** | | **18 Months**** | |
| --- | --- | --- | --- | --- |
|  | **Intervention Group**  **(n = 615)** | **Control Group**  **(n = 610)** | **Intervention Group**  **(n = 615)** | **Control Group**  **(n = 610)** |
| **Medications***** |  |  |  |  |
| *Atomoxetine* | 1 | 0 | 1 | 0 |
| *Fluoxetine* | 2 | 4 | 4 | 6 |
| *Melatonin* | 0 | 0 | 1 | 0 |
| *Mirtazapine* | 0 | 1 | 0 | 1 |
| *Prazosin* | 0 | 1 | 0 | 1 |
| *Promethazine* | 1 | 2 | 1 | 2 |
| *Propranolol* | 0 | 0 | 0 | 1 |
| *Quetiapine* | 0 | 2 | 0 | 2 |
| *Risperidone* | 0 | 0 | 0 | 1 |
| *Sertraline* | 3 | 6 | 5 | 8 |
|  |  |  |  |  |

Data are n (%) unless otherwise indicated. *Offered is defined as medications prescribed within 12- or 18-months post randomisation **18-month column includes all medications offered between randomisation and 18 months ***Numbers within rows show the number of participants who were offered each of the medications. Participants can have more than one medication so may appear in more than one row.

Table 2.52: Secondary outcomes – Any medications offered* within 12 and 18 months

|  | **12 Months** | | **18 Months**** | |
| --- | --- | --- | --- | --- |
|  | **Intervention Group**  **(n = 615)** | **Control Group**  **(n = 610)** | **Intervention Group**  **(n = 615)** | **Control Group**  **(n = 610)** |
| **Medications***** |  |  |  |  |
| *Amitriptyline* | 0 | 0 | 0 | 1 |
| *Aripiprazole* | 1 | 0 | 1 | 0 |
| *Atomoxetine* | 1 | 0 | 1 | 0 |
| *Citalopram* | 0 | 1 | 0 | 1 |
| *Clondine* | 0 | 1 | 0 | 1 |
| *Desmopressin* | 0 | 1 | 0 | 1 |
| *Ensure compact* | 0 | 1 | 0 | 1 |
| *Fluoxetine* | 12 | 6 | 16 | 12 |
| *Fluvoxamine* | 1 | 0 | 1 | 0 |
| *Lisdexamfetamine* | 2 | 0 | 3 | 0 |
| *Lorazepam* | 2 | 0 | 2 | 0 |
| *Melatonin* | 5 | 11 | 7 | 17 |
| *Methylphenidate* | 0 | 8 | 2 | 13 |
| *Mirtazapine* | 0 | 1 | 0 | 1 |
| *Multi-vitamin* | 0 | 0 | 0 | 1 |
| *Otomize* | 1 | 0 | 1 | 0 |
| *Prazosin* | 0 | 1 | 0 | 1 |
| *Promethazine* | 5 | 6 | 8 | 6 |
| *Propranolol* | 1 | 0 | 1 | 1 |
| *Quetiapine* | 0 | 3 | 0 | 3 |
| *Risperidone* | 0 | 0 | 0 | 1 |
| *Sertraline* | 4 | 18 | 10 | 24 |
| *Vitamin D* | 2 | 0 | 2 | 0 |
|  |  |  |  |  |

Data are n (%) unless otherwise indicated.

*Offered is defined as medications prescribed within 12- or 18-months post randomisation **18-month column includes all medications offered between randomisation and 18 months ***Numbers within rows show the number of participants who were offered each of the medications. Participants can have more than one medication so may appear in more than one row.

## Final Analysis Appendix: Time to treatment figures

Figure 2.4: Time to first offered treatment/intervention for an emotional disorder within 12 months

Figure 2.5: Time to start of first treatment/intervention for an emotional disorder within 12 months

Figure 2.6: Time to first offer of any treatment/intervention within 12 months

Figure 2.7: Time to first starting any treatment/intervention within 12 months

Figure 2.8: Time to first offered treatment/intervention for an emotional disorder within 18 months

Figure 2.9: Time to start of first treatment/intervention for an emotional disorder within 18 months

Figure 2.10: Time to first offered any treatment/intervention within 18 months

Figure 2.11: Time to first starting any treatment/intervention within 18 months

## Final Analysis Appendix: Protocol deviations

Table 2.53: Protocol deviations

| Site ID | Deviation |
| --- | --- |
| 03 | Young person DAWBA completed before written assent form. Parental consent had been obtained prior to randomisation, in accordance with the protocol. Written assent from the young person was subsequently documented after DAWBA completion. |
| All | Two issues with the STADIA database were identified:   1) A small number of secondary questionnaires were not sent to participants in error. There was an error in the database script where these questionnaires were erroneously locked if consent from the secondary participant was obtained on a day after that from the primary participant. Questionnaires were sent out to the secondary participants that were still within the completion window.    2)6- and 12-month questionnaires were not automatically sent by the database between 28-Feb-2021 and 21-Mar-2021. Database script was modified and corrected. Database script end date of 27-Feb-2021 was removed so that 6-month and 12-month questionnaires are sent indefinitely.  All 6- and 12-month questionnaires due in that period were sent out to the participants. |
| 03 | Two participants were randomised at similar time to the STADIA trial at site 03.  As is standard practice in the trial at the first opportunity the Research Assistant (RA) printed the DAWBA report (the trial intervention) alongside a covering letter to post out to the trial participants.  The DAWBA report does not contain any directly identifiable information but is annotated with the participant’s ID number.  The cover letter included the parent/guardian’s name and young person’s NHS number as reference.  The DAWBA covering letter and report were accidently mixed up so 03402 received 03408’s documents and vice versa. The breach was reported to the Sponsor who deemed the breach non-serious, REC were informed and asked the trial team to contact both families and offer them the opportunity to meet with the Chief Investigator in order to apologise for the error personally. Both sets of parents were contacted by the site RA in the first instance and declined the opportunity for a further meeting with the Chief Investigator as they were happy that the breach had been dealt with appropriately. REC were satisfied with the actions that we had taken and considered the review of the breach closed with no further action required. |

# Health Economics Analysis

**Supplementary Appendix – Health Economics**

The following presents a more detailed description of the health economic methods and results of the STADIA trial.

**Methods:**

**Resource use and costs~~:~~**

The primary viewpoint for resource use collection and costs was from an NHS and Personal Social Services (PSS) perspective, in accordance with NICE guidance (National Institute for Health and Clinical Excellence, 2023). Resource use data were collected at baseline, 6-months and 12-months using a purposely designed resource use questionnaire informed by the STADIA PPI groups (parent/carer group and youth lab). STADIA NHS & PSS resource use comprise inpatient stays, outpatient attendances, community care (including social care services), prescribed medications and primary care visits. Broader societal costs consider time-off work (productivity losses) and out-of-pocket expenses (travel-related, medication) incurred by families (see Table 3.1). All resource use collection in STADIA related to condition-specific health-care utilisation and costs (i.e., those specifically pertaining to child emotional difficulties). The resource use questionnaire was completed for the child or young person (CYP) by the parent/carer in the 5-10 and 11-15 year age categories; in the 16-17 age category the resource use questionnaire was completed by the CYP.

Table 3.1 provides an overview of all the resource use and costs considered in the economic evaluation.

Table 3.1: Resource use and cost sources

|  | Resource use | Costing sources and technical notes |
| --- | --- | --- |
| **Health service** | | |
| **Intervention costs** | The DAWBA. | Cost taken directly from the youthinmind charity (Youth in Mind, 2012) |
| **Inpatient hospital services** | Hospital admission. | NHS Reference costs (2021-22) (NHS, 2022) |
| **Outpatient hospital services** | Psychologist visits; A&E attendances; Paediatric nurse visits; Paediatric doctor visits; Paediatric therapist visits CAMHS doctor visit; CAMHS nurse visit; CAMHS therapist visit. | NHS Reference costs (2021-22) (NHS, 2022)  PSSRU (2022) (PSSRU) |
| **Primary care** | GP surgery visits; GP home visits; Nurse home visit; nurse practice visit; face to face counsel; online counsel. | NHS Reference costs (2021-22) (NHS, 2022)  PSSRU (2022) (PSSRU) |
| **Community services** | Community health services nurse; health visitor centre; health visitor home visit; social worker visit; Home help/care worker; day care visit; family visit; volunteer visit; respite visit; self-help visit. | NHS Reference costs (2021-22) (NHS, 2022)  PSSRU (2022) (PSSRU) |
| **Medication** | All prescribed medicines related to emotional difficulties. | BNF and eMIT , medication costs interpolated between recorded follow-ups (GOV.UK, 2022; NICE, 2023) |
| **Societal** | | |
| **Productivity** | Time taken off work as a result of child/young person emotional difficulties. | Human capital approach with days off work multiplied by ONS average day salary data (linked to individuals' employment status and occupation) (OFNS, 2021)  Child/Young person’s hourly rate of £5.28 lost due to emotional difficulties (GOV.UK, 2024). |
| **Over-the-counter medications** | Self-reported out-of-pocket payments for over-the-counter medications (including homeopathic remedies and supplements) for the child/young person’s emotional difficulties. | Participant reported monetary price |
| **Out-of-pocket expenses** | Self-reported out-of-pocket payments due to child/young person’s emotional difficulties. | Costs as given. |

Intervention costs were related to the cost for the DAWBA. The current charge, at the time of the trial delivery, is £10 per individual assessed (Youth in Mind, 2012), and as such this cost was applied to each participant across the intervention group of the trial. No time costs were applied to the parents/carers or clinicians completing or reviewing the DAWBA.

Inpatient, outpatient, primary care, and community services were costed according to the number of recorded attendances, multiplied by the relevant unit costs from NHS reference costs (NHS, 2022)and the Personal Social Service Research Unit (PSSRU) (PSSRU) (see Table 3.2). Unit costs were inflated to 2022-23 prices using the PSSRU pay and prices index where necessary (PSSRU). Medication costs considered all medicines prescribed due to emotional difficulties during the trial using drug costs from eMIT and BNF databases (GOV.UK, 2022; NICE, 2023). It was assumed no medication wastage occurred. Since all resource use and broader out-of-pocket costs recorded in the STADIA trial related to “*the previous three months*”, not extending over the full-time horizon between follow-ups (baseline, 6-months, 12-months), costs were extrapolated to previous follow-up (i.e. costs 3-6 months representative of those at 0-3 months; costs 9-12 months representative of those incurred between 6-9 months).

Table 3.2: Unit cost

| **Healthcare unit** | **Cost** | **Source** |
| --- | --- | --- |
| Hospital admission | £729.32 | NHS reference costs "Non-Elective Inpatient - Short Stay (Average)" |
| A&E visit | £223.00 | PSSRU (A&E) |
| CAMHS nurse | £82.18 | PSSRU(2021) |
| CAMHS doctor | £367.07 | NHS reference costs (Child and Adolescent Psychiatry Service, Non-Admitted Non-Face-to-Face Attendance, Follow-up) |
| CAMHS Therapist | £154.64 | NHS ref costs(Other Therapist, Child, One to One) |
| Psychologist visit | £64.00 | PSSRU (Band 7 hospital based scientific and professional staff) |
| Paediatrics Nurse | £83.20 | PSSRU (2021) |
| Paediatrics Doctor | £284.62 | NHS reference costs (Paediatric Service) |
| Paediatrics Therapist | £154.64 | NHS reference costs (Other Therapist, Child, One to One) |
| GP practice visit | £41.00 | PSSRU (GP per hour of patient contact including direct care staff costs) |
| GP home visit | £159.00 | PSSRU (GP per time spent at home visit) |
| GP practice visit with nurse | £7.99 | PSSRU (Cost estimations for GP practice Nurse) |
| GP nurse home visit | £33.80 | PSSRU (Nurse spent at home visit) |
| School Nurse | £49.21 | NHS reference costs (School Based Children's Health Special Schools Nursing) |
| Community health service nurse visit | £49.21 | NHS reference costs (School Based Children's Health Special Schools Nursing) |
| Community health service health visitor (at the health centre) | £19.88 | (PSSRU 2022) |
| Community health service health visitor (home visit) | £32.80 | (PSSRU 2022) |
| Face to face counselling service meeting | £55.00 | PSSRU (Community based scientific and professional staff) |
| Online counselling meeting | £55.00 | PSSRU (Community based scientific and professional staff) |
| Community support social worker home visit | £53.41 | PSSRU (Social worker 2022) |
| Community support home help / care worker visit | £23.00 | PSSRU (Home care worker) |
| Day care centre visit | £412.92 | NHS reference costs (Children and Adolescent Mental Health Services, Day Care Facilities) |
| Volunteer visit | £30.56 | PSSRU (Transition services for children: low-cost) |
| Respite day | £167.30 | NHS ref costs (Specialist Nursing, Palliative/Respite Care, Child, Face to face) |
| Self-help group attendance | £154.64 | NHS ref costs (Other Therapist, Child, One to One) |
| Family worker | £59.76 | PSSRU (Family support worker) |

A&E: Accident and emergency; PSSRU: Personal Social Services Research Unit; GP: General practitioner; CAMHS: Child and Adolescent Mental Health Services

Broader societal out-of-pocket expenses and over-the-counter medication costs were taken from the costs directly reported by participants during the trial. Productivity costs were calculated for parents using the human capital approach, specifically multiplying parents’ self-reported absenteeism due to their child’s emotional difficulties by the Standard Occupational classifications (SOC) salary costs from the office for National Statistics (OFNS, 2021) (see Table 3.3 for SOCs). Participants in the 16-17 age group also had productivity losses from lost work or apprenticeship hours. This was calculated using the national minimum wage for those under the age of 18 (GOV.UK, 2024).

Table 3.3: Standard occupational classifications and costs (ONS, 2021) [18]

| Job title | Full-time | Part-time |
| --- | --- | --- |
| Manager/administrator | £171.40 | £48.20 |
| Professional (health, teaching, legal) | £159.40 | £81.40 |
| Associate professional (technical, nursing) | £121.60 | £50.00 |
| Clerical worker/ Secretary | £95.80 | £44.60 |
| Services/Sales | £83.20 | £36.80 |
| Skilled labourer (building, electrical) | £110.20 | £42.00 |
| Factory worker | £85.60 | £27.80 |
| Other | £128.00 | £45.60 |

**Outcomes:**

The primary outcome used in the cost effectiveness analysis was quality-adjusted life years (QALYS), a generic health measure in which the benefits, in terms of length of life, are adjusted to reflect quality of life (NICE). One quality adjusted life year (QALY) is equal to 1 year of life in perfect health. The health-related quality of life (HRQoL) of trial participants was assessed using the EQ-5D-Y, a questionnaire that is specifically adapted so the EQ-5D dimensions are appropriate for the measurement of health-related quality of life in young people. The EQ-5D-Y asks participants to rate their health according to five broad health dimensions (mobility, self-care, usual activity, pain/discomfort and anxiety/depression) at three levels of severity (no problems, some problems and a lot of problems) (Group.). Due to the absence of UK preferencing-based weighting for the EQ-5D-Y the health-related quality of life associated with each health state was calculated using the EQ-5D-3L value set (10). A proxy report where parents/carers asked rated their child’s health-related quality of life in their (the proxy’s) opinion was used for participants aged 5-15 years, while for participants aged 16-17 the results from the young adults EQ-5D-Y were used to inform the final analysis. Study outcomes were assessed at baseline, 6-month and 12-month follow-ups.

The HRQoL of trial participants was also assessed using the Child Health Utility 9 Dimensions (CHU9D), a paediatric generic preference-based measure of HRQoL. The CHU9D questionnaire consists of nine individual items (worried, sad, pain, tired, annoyed, schoolwork/homework, sleep, daily routine and able to join in activities) with five levels (no problems, few problems, some problems, many problems and can’t) (Stevens, 2009). It provides a descriptive profile and a set of preference weights, giving utility values for each health state described by the descriptive system, allowing the calculation of quality adjusted life years (QALYs). The CHU9D was completed by participants aged 11 and over at baseline, 6 months, and 12 months, with proxy versions completed by the parent/carer for all age groups.

The HRQoL of the parents/carers of trial participants was assessed using the EQ-5D-5L. The EQ-5D-5L includes five broad health dimensions (mobility, self-care, usual activity, pain/discomfort and anxiety/depression) at five levels of severity (no problems, slight problems, moderate problems, serve problems and I am unable to) (Group.). An English preferencing-based(Devlin, Shah, Feng, Mulhern, & van Hout, 2018) value set was used to calculate the health-related quality of the parents/carers.

QALYs for each participant were calculated using an area under the curve approach using linear interpolation in HRQoL between time points. Base case cost effectiveness analyses considered QALYs using general population UK EQ-5D-3L preference values for EQ-5D-Y responses(Dolan, 1997). The time horizon of the analysis was 12-months, the length of the STADIA study for the primary outcome. Costs and QALYs were not discounted as they accrue within a 12-month period.

**Economic analysis:**

The economic analysis compared health service (NHS & PSS) and societal costs both within resource categories and in total to give total aggregated costs for each perspective. Cost, QALY and cost effectiveness comparisons are presented using imputed data. Cost effectiveness was assessed using incremental cost effectiveness ratios and incremental net-monetary benefits (Drummond, Schulpher, Claxton, Stoddart, & Torrance, 2015).

With complete case data in costs and outcomes at only 29% and 53% of the trial population respectively (see Table 3.4), missing cost and outcome data were imputed to increase efficiency and reduce the risk of bias, (Faria, Gomes, Epstein, & White, 2014). To most accurately predict missing values, and to keep imputed values within their inherent limits, incomplete data were populated using multiple imputation by chained equations with predicted mean matching (White, Royston, & Wood, 2011). All subsequent analyses of multiple data sets followed Rubin’s rules (Little & Rubin, 2019).

Table 3.4: Missingness in costs & outcomes

| **Cost data** | | | | | | | | | | | | | | | | | |
| --- | --- | --- | --- | --- | --- | --- | --- | --- | --- | --- | --- | --- | --- | --- | --- | --- | --- |
|  | | Baseline | | | Week 26 | | | | Week 52 | | | | Total | | | | |
| **NHS Perspective** | | | | | | | | | | | | | | | | | |
|  | | N | | % | N | | % | | N | | % | | N | | % | | |
| Total inpatient | | 44 | | 4% | 414 | | 34% | | 392 | | 32% | | 527 | | 43% | | |
| Total outpatient | | 116 | | 9% | 482 | | 39% | | 436 | | 36% | | 617 | | 50% | | |
| Total community including social care services | | 126 | | 10% | 449 | | 37% | | 417 | | 34% | | 571 | | 47% | | |
| Total primary | | 154 | | 13% | 507 | | 41% | | 471 | | 38% | | 647 | | 54% | | |
| Total medication | | 6 | | 0% | 332 | | 27% | | 472 | | 39% | | 474 | | 39% | | |
| Total NHS costs | | 301 | | 25% | 615 | | 50% | | 661 | | 54% | | 780 | | 64% | | |
| **Broader Perspective** | | | | | | | | | | | | | | | | | |
|  | | N | | % | N | | % | | N | | % | | N | | % | | |
| Productivity loss | | 157 | | 13% | 489 | | 40% | | 456 | | 37% | | 591 | | 48% | | |
| Other medication | | 24 | | 2% | 385 | | 31% | | 353 | | 29% | | 479 | | 39% | | |
| Out-of-pocket expenses | | 116 | | 9% | 466 | | 38% | | 433 | | 35% | | 549 | | 45% | | |
| Total societal costs | | 183 | | 15% | 503 | | 41% | | 469 | | 38% | | 608 | | 50% | | |
| **Total costs** | | | | | | | | | | | | | | | | | |
| Total costs | | 449 | | 37% | 713 | | 58% | | 741 | | 60% | | 864 | | 71% | | |
| **Outcome data** | | | | | | | | | | | | | | | | |  |
|  | Week 0 | | | | | Week 26 | | | | Week 52 | | | | Total | | |  |
|  | N | | % | | | N | | % | | N | | % | | N | | % |  |
| EQ-5D-Y | 198 | | 16% | | | 364 | | 30% | | 346 | | 28% | | 572 | | 47% |  |
| CHU9D | 200 | | 16% | | | 363 | | 30% | | 345 | | 28% | | 576 | | 47% |  |
| EQ-5D-5L | 121 | | 10% | | | 460 | | 38% | | 429 | | 35% | | 548 | | 45% |  |

For the base case cost effectiveness analysis, between group differences in costs and outcomes were estimated using multiply imputed data and seemingly-unrelated regressions, a simultaneous method permitting baseline and covariate adjustments while capturing the multivariate distributions of outcomes by allowing correlation between the error terms of regressions. Regression analyses included site id, participant age (categorical 5-10, 10-15 and 16-17 years old), sex (trial minimisation variables) and treatment covariates. To control for broader imbalances in baseline HRQoL between arms, QALY regression analyses also controlled for baseline EQ-5D preference values (Manca, Hawkins, & Sculpher, 2005). Uncertainty in the estimated treatment regression coefficients in the models were simulated with 10,000 iterations assuming multivariate normality of the regression coefficients.

For each analytical approach cost effectiveness was assessed according to incremental cost effectiveness ratios (ICERs). The probability that an intervention is cost effective was calculated via the proportion of simulated costs and QALYs that would be judged as cost effective. The probability of being cost effective was calculated up to a £30,000 per QALY cost-effectiveness threshold, the upper-bound used by the National Institute for Health and Care Excellence (NICE, 2013). The probability that the DAWBA would be considered cost effective compared to control was plotted in a cost effectiveness acceptability curve (CEAC) (Fenwick, Claxton, & Sculpher, 2001)

**Results:**

**Outcomes:**

Table 3.5 presents the average imputed EQ-5D-Y, CHU9D and parental/carer EQ-5D-5L preference scores for the intervention and control group. Baseline EQ-5D-Y scores were higher for the control group relative to the intervention (-0.0106). Scores increased in both groups over the duration of the trial. The control group extended and then maintained higher scores relative to the intervention group over the course of the trial. EQ-5D-Y scores translated into an average QALY loss of 0.0232 for the intervention group relative to the control. In line with the EQ-5D-Y, differences in CHU9D preference scores between the arms did not exceed baseline differences for the intervention. Preference values were generally higher and similar between arms when using the CHU9D than the EQ-5D-Y. CHU9D scores translated into an average QALY gain of 0.0028 for the intervention group relative to the control group, driven by differences in baseline values. The EQ-5D-5L parental/carer preference scores have a trajectory similar to the EQ-5D-Y with the control group having higher scores throughout the trial (particularly at 6-months). The scores from the EQ-5D-5L translated to an average QALY loss of 0.0124 for the intervention group relative to the control group.

Table 3.5: Base case outcome table

|  | **Intervention group** | **Control group** | **Mean Difference** |
| --- | --- | --- | --- |
| **Outcomes:** | Mean values by timepoint | Mean values by timepoint | Mean values by timepoint  **(95% confidence interval)** |
| **EQ-5D-Y** |  |  |  |
| Baseline | 0.4862 | 0.4968 | -0.0106  (-0.0505, 0.0293) |
| 6 months | 0.5679 | 0.5993 | -0.0313  (-0.0742, 0.0116) |
| 12 months | 0.6072 | 0.6269 | -0.0197  (-0.0616, 0. 0221) |
| **QALYS (EQ-5D-Y)** | 0.5573 | 0.5805 | -0.0232  (-0.0572, 0. 0107) |
| **CHU9D** |  |  |  |
| Baseline | 0.7291 | 0.7247 | 0.0044  (-0.0109, 0.0196) |
| 6 months | 0.7617 | 0.7584 | 0.0033  (-0.0138, 0.0204) |
| 12 months | 0.7709 | 0.7706 | 0.0003  (-0.0173, .0180) |
| **QALYs (CHU9D)** | 0.7558 | 0.7530 | 0.0028  (-0.0110, 0.0167) |
| **EQ-5D-5L** |  |  |  |
| Baseline | 0.7856 | 0.7978 | -0.0122  (-0.0365, 0.0121) |
| 6 months | 0.7820 | 0.7971 | -0.0151  (-.04286, 0.0127) |
| 12 months | 0.7956 | 0.8028 | -0.0072  (-0.0349, 0.0205) |
| **QALYs (EQ-5D-5L)** | 0.7863 | 0.7987 | -0.0124  (-0.0349, 0.0101) |

**Sub-group analysis:**

Table 3.6 presents the costs and outcomes from participants who had completed all follow-up information for costs and outcomes respectively (i.e. complete data). Costs from an NHS & PSS perspective were £195.44 lower in the intervention arm compared to the control. This was mainly driven by differences in Outpatient care utilisation. When accounting for societal costs the intervention was found to be cost saving by £276.19. This was primarily driven by differences in non-medication related out-of-pocket expenses. In this sub-group, HRQoL measurements remain lower in the intervention arm compared to control (QALYs losses of 0.0196). Unlike the base case finding, the results for this subgroup analysis do find modest cost reductions associated with the intervention, albeit with inferior preference scores compared to control. It is important to note that the NHS & PSS cost savings associated with the DAWBA in this subgroup do not offset the QALY losses at a £20,000/QALY threshold (£9,975 saved per QALY lost). These results must be interpreted with caution given the smaller, and likely unrepresentative, sample sizes.

Table 3.6: Complete case results

|  | Intervention (n=325) | Usual care (n=325) | Difference |
| --- | --- | --- | --- |
| **Outcomes** |  |  |  |
| EQ-5D-Y Baseline | 0.4779 | 0.4903 | -0.0124 |
| EQ-5D-Y 6 month | 0.5644 | 0.5873 | -0.0229 |
| EQ-5D-Y 12 month | 0.5974 | 0.6177 | -0.0202 |
| QALYs | 0.5510 | 0.5706 | -0.0196 |
| **NHS and PSS costs** | (n=179) | (n=182) |  |
| Intervention | £10.00 | £0.00 | £10.00 |
| Inpatient | £8.15 | £0.00 | £8.15 |
| Outpatient | £623.65 | £848.71 | -£225.06 |
| Community including social care services | £123.05 | £82.02 | £41.03 |
| Primary | £432.15 | £472.27 | -£40.12 |
| Medication | £16.15 | £5.59 | £10.56 |
| **Broader costs** |  |  |  |
| Productivity losses | £387.45 | £361.54 | £25.92 |
| Over-the-counter medication | £16.32 | £18.93 | -£2.61 |
| Out-of-pocket expenses | £746.96 | £1,046.46 | -£299.50 |
| **Total costs** |  |  |  |
| **Total NHS and PSS costs** | £1,213.15 | £1,408.59 | -£195.44 |
| **Total broader costs** | £1,150.74 | £1,426.93 | -£276.19 |
| **Total societal costs**  **(NHS + broader)** | £2,363.89 | £2,835.52 | -£471.63 |

**CE outputs:**

Figure 3.1: Base case cost-effectiveness scatter plot


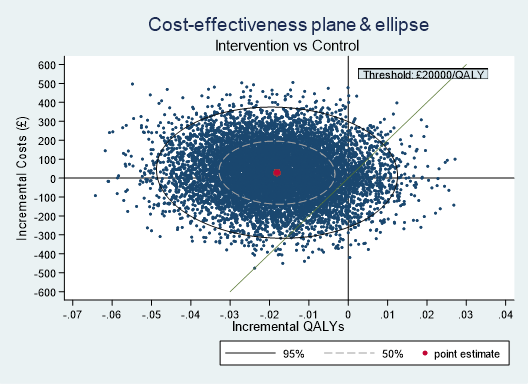


Figure 3.2: Societal perspective scatter plot


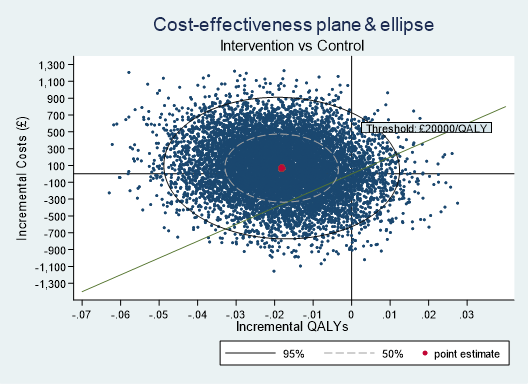


Figure 3.3: Base case Cost-Effectiveness Acceptability Curve


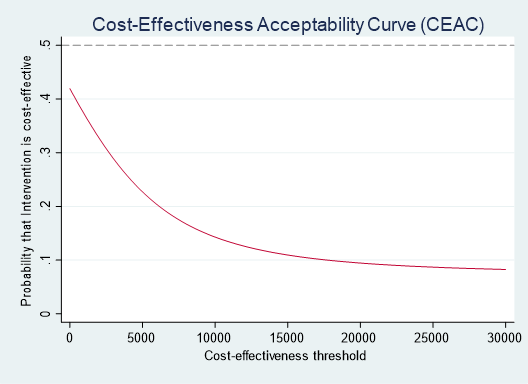


Figure 3.4: Societal perspective Cost-Effectiveness Acceptability Curve


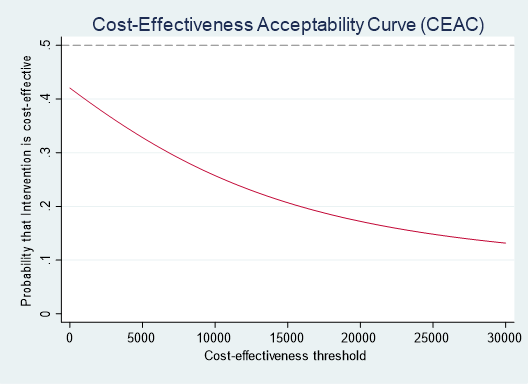


Table 3.7: Base case seemingly-unrelated regression analysis (NHS & PSS cost perspective)

| **Seemingly-unrelated QALY regression** | | | | | | |
| --- | --- | --- | --- | --- | --- | --- |
|  | Coefficient | Std. err. | t | P>t | [95% conf. | |
| Treatment | -0.0181 | 0.0125 | -1.45 | 0.149 | -0.0427 | 0.0065 |
| Baseline EQ5D-Y | 0.6022 | 0.0193 | 31.14 | 0 | 0.5641 | 0.6403 |
| Age^*^ |  |  |  |  |  |  |
| *10-15* | 0.0090 | 0.0140 | 0.64 | 0.52 | -0.0185 | 0.0365 |
| *16-17* | -0.0495 | 0.0215 | -2.3 | 0.022 | -0.0917 | -0.0073 |
| female | -0.0212 | 0.0134 | -1.58 | 0.115 | -0.0475 | 0.0052 |
| Site^**^ |  |  |  |  |  |  |
| *2* | 0.0004 | 0.0208 | 0.02 | 0.983 | -0.0404 | 0.0412 |
| *3* | -0.0170 | 0.0195 | -0.87 | 0.383 | -0.0555 | 0.0214 |
| *4* | 0.0476 | 0.0234 | 2.03 | 0.043 | 0.0014 | 0.0939 |
| *5* | -0.0049 | 0.0214 | -0.23 | 0.818 | -0.0470 | 0.0371 |
| *6* | 0.0236 | 0.0363 | 0.65 | 0.515 | -0.0477 | 0.0950 |
| *7* | 0.0848 | 0.0450 | 1.88 | 0.06 | -0.0037 | 0.1733 |
| *8* | -0.0699 | 0.0919 | -0.76 | 0.447 | -0.2501 | 0.1104 |
| Constant | 0.2926 | 0.0213 | 13.74 | 0 | 0.2506 | 0.3345 |
| **Seemingly-unrelated NHS & PSS cost regression** | | | | | | |
| Treatment | 28.80 | 141.53 | 0.2 | 0.839 | -249.41 | 307.01 |
| Age^*^ |  |  |  |  |  |  |
| *10-15* | 513.84 | 156.37 | 3.29 | 0.001 | 206.67 | 821.00 |
| *16-17* | 509.52 | 240.81 | 2.12 | 0.035 | 36.34 | 982.71 |
| female | 235.74 | 145.77 | 1.62 | 0.106 | -50.52 | 522.00 |
| Site^**^ |  |  |  |  |  |  |
| *2* | -152.25 | 247.91 | -0.61 | 0.54 | -641.02 | 336.51 |
| *3* | 343.03 | 227.07 | 1.51 | 0.133 | -105.08 | 791.15 |
| *4* | 248.40 | 265.34 | 0.94 | 0.35 | -274.84 | 771.64 |
| *5* | 12.08 | 240.84 | 0.05 | 0.96 | -462.03 | 486.20 |
| *6* | 135.55 | 428.92 | 0.32 | 0.752 | -708.71 | 979.81 |
| *7* | -256.45 | 526.18 | -0.49 | 0.626 | -1292.09 | 779.18 |
| *8* | 5467.09 | 1109.20 | 4.93 | 0 | 3287.42 | 7646.76 |
| Constant | 971.42 | 218.85 | 4.44 | 0 | 539.84 | 1403.01 |

^* Age reference category: <10 years old; ** Site reference category: site 1^

Table 3.8: Broader societal seemingly-unrelated regression analysis

| **Seemingly-unrelated QALY regression** | | | | | | |
| --- | --- | --- | --- | --- | --- | --- |
|  | Coefficient | Std. err. | t | P>t | [95% conf. | |
| Treatment | -0.0182 | 0.0125 | -1.45 | 0.147 | -0.0427 | 0.0064 |
| Baseline EQ5D-Y | 0.5976 | 0.0192 | 31.17 | 0 | 0.5599 | 0.6354 |
| Age^*^ |  |  |  |  |  |  |
| *10-15* | 0.0089 | 0.0140 | 0.64 | 0.524 | -0.0186 | 0.0364 |
| *16-17* | -0.0501 | 0.0215 | -2.33 | 0.02 | -0.0923 | -0.0079 |
| female | -0.0212 | 0.0134 | -1.58 | 0.114 | -0.0475 | 0.0051 |
| Site^**^ |  |  |  |  |  |  |
| *2* | 0.0006 | 0.0207 | 0.03 | 0.976 | -0.0402 | 0.0414 |
| *3* | -0.0172 | 0.0195 | -0.88 | 0.38 | -0.0556 | 0.0213 |
| *4* | 0.0481 | 0.0234 | 2.05 | 0.042 | 0.0019 | 0.0943 |
| *5* | -0.0047 | 0.0213 | -0.22 | 0.825 | -0.0468 | 0.0373 |
| *6* | 0.0239 | 0.0363 | 0.66 | 0.51 | -0.0474 | 0.0953 |
| *7* | 0.0856 | 0.0450 | 1.9 | 0.058 | -0.0030 | 0.1741 |
| *8* | -0.0697 | 0.0919 | -0.76 | 0.448 | -0.2499 | 0.1106 |
| Constant | 0.2949 | 0.0211 | 13.95 | 0 | 0.2533 | 0.3365 |
| **Seemingly-unrelated broader societal cost regression** | | | | | | |
| Treatment | 68.98 | 343.81 | 0.2 | 0.841 | -607.66 | 745.62 |
| Age^*^ |  |  |  |  |  |  |
| 10-15 | 780.68 | 352.68 | 2.21 | 0.027 | 88.95 | 1472.40 |
| 16-17 | 865.50 | 738.80 | 1.17 | 0.245 | -608.24 | 2339.24 |
| female | 380.49 | 357.51 | 1.06 | 0.288 | -322.63 | 1083.61 |
| Site^**^ |  |  |  |  |  |  |
| *2* | -288.76 | 620.40 | -0.47 | 0.642 | -1515.79 | 938.27 |
| *3* | 802.16 | 539.72 | 1.49 | 0.139 | -263.21 | 1867.53 |
| *4* | -1.35 | 632.93 | 0 | 0.998 | -1250.01 | 1247.30 |
| *5* | -106.59 | 569.70 | -0.19 | 0.852 | -1228.07 | 1014.88 |
| *6* | 94.68 | 1071.14 | 0.09 | 0.93 | -2019.67 | 2209.04 |
| *7* | -1206.04 | 1117.96 | -1.08 | 0.281 | -3398.03 | 985.96 |
| *8* | 8050.36 | 2859.34 | 2.82 | 0.005 | 2407.66 | 13693.06 |
| Constant | 1993.28 | 525.92 | 3.79 | 0 | 955.26 | 3031.29 |

^* Age reference category: <10 years old; ** Site reference category:site 1^

**HEAP Deviations:**

Table 3.9: HEAP deviations

| **Outlined in HEAP** | **Reason for deviation** |
| --- | --- |
| *“Potential additional analysis may occur at the 18-month time point regarding confirmed diagnosis and acceptance of referral and changes from the 12 month time point”* | Although the service-related outcome components were funded for additional analysis up to 18 months it was deemed an unnecessary expense for this to include health economics so further funding was not requested and deviation was unnecessary |
| *“In addition to time off education, the proforma collects further data such as a young person’s use of an after-school club or teaching assistant, and meetings with SENDCO (Special Education Needs & Disabilities Co-ordinator), head of year, or school counsellor. Other use is also requested as an open-ended question. These resources, while not traditionally considered within CEA, will be costed using staffing bands and assumptions of duration of contact, or other appropriate unit costs where available.”* | Data were collected on education, however due to the primary analysis study taking an NHS and personal social services perspective, educational costs and effects were not considered here. Poor data completion further justified this approach. |
| *“Administrative records of treatments/interventions offered by CAMHS during the trial period may be considered as a supplementary source of data”* | The trial focused on diagnostic outcomes. As CAMHS administrative records not capture detailed service use outside of CAMHS, participant-reported records were the primary source of data. |
| *“The qualitative research revealed that forms are filled in during participants’ leisure time and as such a value of 25% average wage rate could be used.”* | This was deemed an unnecessary inclusion and such the intervention cost remained at £10. Note this could be considered a conservative estimate of the intervention cost. |

Table 3.10: Medication unit costs

| **Primary name** | **Cost** | **Unit** | **Cost per pill** | **Dosage** | **Source** |
| --- | --- | --- | --- | --- | --- |
| Amitriptyline 10mg | £0.24 | 28 | £0.00873 | 10mg | eMIT |
| Amitriptyline 25mg | £0.29 | 28 | £0.01036 | 25mg | eMIT |
| Aripriprazole | £14.92 | 28 | £0.53303 | 5mg | eMIT |
| Atomoxetine | £4.11 | 28 | £0.14679 | 40mg | eMIT |
| Beta blockers | £0.77 | 28 | £0.02750 | 10mg | eMIT |
| Buscopan | £3.83 | 56 | £0.06839 | 10mg | BNF |
| Circadin 2mg | £6.53 | 30 | £0.21767 | 2mg | BNF |
| Circadin 3mg | £12.81 | 30 | £0.42700 | 3mg | BNF |
| Citalopram 10mg | £0.36 | 28 | £0.01291 | 10mg | eMIT |
| Citalopram 20mg | £0.54 | 28 | £0.01929 | 20mg | eMIT |
| Clonidine | £10.43 | 112 | £0.09309 | 0.025mg | eMIT |
| Concerta XL | £42.45 | 30 | £1.41500 | 36mg | BNF |
| Contraception pill | £3.40 | 84 | £0.04048 | 75mg | BNF |
| Cycilzine | £3.87 | 100 | £0.03870 | 50mg | BNF |
| Delmosart | £42.45 | 30 | £1.41500 | 36mg | BNF |
| Desmopressin | £13.25 | 10 | £1.32512 | 4mg | eMIT |
| Diazepam | £0.83 | 28 | £0.02964 | 2mg | BNF |
| elvanse 30mg | £58.24 | 28 | £2.08000 | 30mg | BNF |
| elvance 40mg | £62.82 | 28 | £2.24357 | 40mg | BNf |
| Equisym 10mg | £25.00 | 30 | £0.83333 | 10mg | BNF |
| Equisym 20mg | £30.00 | 30 | £1.00000 | 20mg | BNF |
| Fluoxetine 10mg | £19.68 | 30 | £0.65600 | 10mg | BNF |
| Fluoxetine 20mg | £1.24 | 30 | £0.04133 | 20mg | BNF |
| Fluoxetine 30mg | £3.18 | 30 | £0.10600 | 30mg | BNF |
| Fluoxetine 40mg | £2.50 | 30 | £0.08333 | 40mg | BNF |
| Fluoxetine 60mg | £2.06 | 30 | £0.06867 | 60mg | BNF |
| Fluoxetine 5ml | £12.77 | 70 | £0.18243 | 5ml | BNF |
| Folic acid | £3.52 | 90 | £0.03911 | 400mcg | BNF |
| Guanfacine | £56.00 | 28 | £2.00000 | 1mg | BNF |
| Imipramine | £2.23 | 28 | £0.07964 | 25mg | eMIT |
| intuniv 1mg | £56.00 | 28 | £2.00000 | 1mg | BNF |
| Intuniv 2mg | £58.52 | 28 | £2.09000 | 2mg | BNF |
| Lactulose | £2.51 | 1 | £2.51000 | 10ml | BNF |
| Lisdexamfetmine | £54.62 | 28 | £1.95071 | 20mg | BNF |
| Loperamide | £2.24 | 30 | £0.07467 | 2mg | BNF |
| Lorazepam | £1.53 | 28 | £0.05464 | 1mg | BNF |
| Medikinet 10mg | £2.88 | 30 | £0.09600 | 10mg | BNF |
| Medikinet 20mg | £10.92 | 30 | £0.36400 | 20mg | BNF |
| Medikinet 30mg | £35.00 | 30 | £1.16667 | 30mg | BNF |
| Medikinet 40mg | £57.72 | 30 | £1.92400 | 40mg | BNF |
| Melatonin 1mg | £29.01 | 60 | £0.48355 | 1mg | eMIT |
| Melatonin 2mg | £10.39 | 30 | £0.34649 | 2mg | eMIT |
| Melatonin 3mg | £19.81 | 30 | £0.66033 | 3mg | BNF |
| Melatonin 4mg | £20.23 | 30 | £0.67433 | 4mg | BNF |
| Melatonin 1ml | £129.18 | 150 | £0.86120 | 1ml | BNF |
| Methlyphenidate 5mg | £3.03 | 30 | £0.10100 | 5mg | BNF |
| Methlyphenidate 10mg | £2.88 | 30 | £0.09600 | 10mg | BNF |
| Mirtazapine | £1.01 | 28 | £0.03607 | 15mg | BNF |
| Nasonex spray 50mcg | £2.76 | 1 | £2.76000 | 50mcg | BNF |
| Omeprazole | £8.78 | 28 | £0.31357 | 10mg | BNF |
| Oxybutynin | £23.47 | 56 | £0.41911 | 3mg | BNF |
| Phenergan 25mg | £39.36 | 56 | £0.70286 | 25mg | BNF |
| Phenergan ml | £6.74 | 10 | £0.67400 | 5mg/5ml | BNF |
| Piraton | £4.74 | 10 | £0.47400 | 4mg | BNF |
| Pizotifen | £1.53 | 28 | £0.05464 | 500mcg | BNF |
| Prazosin | £2.69 | 60 | £0.04483 | 500mcg | BNF |
| Prochlorperazine 5mg | £1.31 | 28 | £0.04679 | 5mg | BNF |
| Promethazine 5mg | £3.28 | 100 | £0.03280 | 5mg | BNF |
| Promethazine 10mg | £31.83 | 56 | £0.56839 | 10mg | BNF |
| Promethazine 25mg | £29.26 | 56 | £0.52250 | 25mg | BNF |
| Promethazine Hydrocholride 10mg | £31.83 | 56 | £0.56839 | 10mg | BNF |
| Propanalol 10mg | £0.78 | 28 | £0.02786 | 10mg | BNF |
| Propranolol 40mg | £0.79 | 28 | £0.02821 | 40mg | BNF |
| Ranitidine | £0.65 | 60 | £0.01083 | 150mg | emit 2019 |
| Quetiapine 50mg | £4.69 | 60 | £0.07819 | 50mg | emit |
| Sertraline 25mg | £15.61 | 28 | £0.55750 | 25mg | BNF |
| Sertraline 50mg | £1.04 | 28 | £0.03714 | 50mg | BNF |
| Sertraline 100mg | £1.22 | 28 | £0.04357 | 100mg | BNF |
| Sertraline 150mg | £13.50 | 30 | £0.45000 | 150mg | BNF |
| sertraline 200mg | £18.00 | 30 | £0.60000 | 200mg | BNF |
| Skandishake | £11.20 | 1 | £11.20000 | 85mg | BNF |
| Strattera 25mg | £13.72 | 28 | £0.49000 | 25mg | BNF |
| Strattera 40mg | £13.40 | 28 | £0.47857 | 40mg | BNF |
| Sumatriptan | £1.38 | 6 | £0.23000 | 100mg | BNF |
| velafaxine | £3.74 | 56 | £0.06679 | 37.5mg | BNF |
| vitamin D | £8.42 | 84 | £0.10024 | 400 unit | BNF |
| Xaggitin XL | £42.45 | 30 | £1.41500 | 36mg | BNF |

**References**

Angold, A., Costello, E. J., Messer, S. C., & Pickles, A. (1995). Development of a short questionnaire for use in epidemiological studies of depression in children and adolescents. *International Journal of Methods in Psychiatric Research, 5*(4), 237-249.

Chorpita, B. F., Yim, L., Moffitt, C., Umemoto, L. A., & Francis, S. E. (2000). Assessment of symptoms of DSM-IV anxiety and depression in children: a revised child anxiety and depression scale. *Behaviour Research and Therapy, 38*(8), 835-855.

Daviss, W. B., Birmaher, B., Melhem, N. A., Axelson, D. A., Michaels, S. M., & Brent, D. A. (2006). Criterion validity of the Mood and Feelings Questionnaire for depressive episodes in clinic and non-clinic subjects. *47*, 927-934.

Devlin, N. J., Shah, K. K., Feng, Y., Mulhern, B., & van Hout, B. (2018). Valuing health-related quality of life: An EQ-5D-5L value set for England. *Health Econ, 27*(1), 7-22.

Dolan, P. (1997). Modeling valuations for EuroQol health states. *Med Care, 35*(11), 1095-1108.

Drummond, M., Schulpher, M., Claxton, K., Stoddart, L., & Torrance, G. (2015). *Methods for the Economic Evaluation of Health Care Programmes* (4th ed.): Oxford University Press

Faria, R., Gomes, M., Epstein, D., & White, I. R. (2014). A guide to handling missing data in cost-effectiveness analysis conducted within randomised controlled trials. *Pharmacoeconomics, 32*(12), 1157-1170.

Fenwick, E., Claxton, K., & Sculpher, M. (2001). Representing uncertainty: the role of cost-effectiveness acceptability curves. *Health Econ, 10*(8), 779-787.

Goodman, R. (1999). The Extended Version of the Strengths and Difficulties Questionnaire as a Guide to Child Psychiatric Caseness and Consequent Burden. *Journal of Child Psychology and Psychiatry, 40*(5), 791-799.

GOV.UK. (2022). Drugs and pharmaceutical electronic market information tool (eMIT). Retrieved 31/01/2024, from <https://www.gov.uk/government/publications/drugs-and-pharmaceutical-electronic-market-information-emit>

GOV.UK. (2024). National Minimum Wage and National Living Wage rates. Retrieved 31/01/2024, from <https://www.gov.uk/national-minimum-wage-rates>

Group., E. EQ-5D instruments – EQ-5D. from <https://euroqol.org/information-and-support/>

Herdman, M., Gudex, C., Lloyd, A., Janssen, M., Kind, P., Parkin, D., et al. (2011). Development and preliminary testing of the new five-level version of EQ-5D (EQ-5D-5L). [journal article]. *Quality of Life Research, 20*(10), 1727-1736.

Kroenke, K., Spitzer, R. L., & Williams, J. B. (2001). The PHQ-9: validity of a brief depression severity measure. *Journal of general internal medicine, 16*(9), 606-613.

Little, R., & Rubin, D. (2019). *Statistical Analysis with Missing Data*: Wiley.

Manca, A., Hawkins, N., & Sculpher, M. J. (2005). Estimating mean QALYs in trial-based cost-effectiveness analysis: the importance of controlling for baseline utility. *Health Econ, 14*(5), 487-496.

National Institute for Health and Clinical Excellence. (2023). NICE health technology evaluations: the manual. NICE process and methods [PMG36]. Retrieved 16/01/2024, from <https://www.nice.org.uk/process/pmg36/chapter/introduction-to-health-technology-evaluation>

NHS. (2022). National Health Service (NHS Improvement). Reference costs 2021-22. Retrieved 31/01/2024, from <https://www.england.nhs.uk/publication/2021-22-national-cost-collection-data-publication/>

NICE. National Institute for Health and Care Excellence (NICE): Glossary. from <https://www.nice.org.uk/glossary?letter=q>

NICE. (2013). National Institute for Health and Care Excellence. Guide to the methods of technology appraisal 2013. NICE process and methods [PMG9]. Retrieved 31/01/2024, from [www.nice.org.uk/process/pmg9](https://uniofnottm.sharepoint.com/sites/STADIATrialManagementGroup/Shared%20Documents/STADIA%20Write%20up/Main%20RCT%20write%20up/Journal%20of%20Child%20Psychology%20and%20Psychiarty/www.nice.org.uk/process/pmg9)

NICE. (2023). British National Formulary (BNF).

OFNS. (2021). Office for National Statistics. Census 2021. Earnings and working hours Retrieved 31/01/2024, from <https://www.ons.gov.uk/employmentandlabourmarket/peopleinwork/earningsandworkinghours>

Perrin, S., Meiser-Stedman, R., & Smith, P. (2005). The children's revised impact of event scale (CRIES): Validity as a screening instrument for PTSD. *Behavioural and Cognitive Psychotherapy, 33*(4), 487-498.

PSSRU. Personal Social Services Research Unit (PSSRU). Unit Costs of Health and Social Care. Retrieved 31/01/2024, from <https://www.pssru.ac.uk/project-pages/unit-costs/>

Spitzer, R. L., Kroenke, K., Williams, J. W., & Löwe, B. (2006). A brief measure for assessing generalized anxiety disorder: The gad-7. *Archives of Internal Medicine, 166*(10), 1092-1097.

Stevens, K. (2009). Developing a descriptive system for a new preference-based measure of health-related quality of life for children. [journal article]. *Quality of Life Research, 18*(8), 1105-1113.

White, I. R., Royston, P., & Wood, A. M. (2011). Multiple imputation using chained equations: Issues and guidance for practice. *Stat Med, 30*(4), 377-399.

Wille, N., Badia, X., Bonsel, G., Burström, K., Cavrini, G., Devlin, N., et al. (2010). Development of the EQ-5D-Y: a child-friendly version of the EQ-5D. *Qual Life Res, 19*(6), 875-886.

Wood, A., Kroll, L., Moore, A., & Harrington, R. (1995). Properties of the mood and feelings questionnaire in adolescent psychiatric outpatients: a research note. *36*, 327-334.

Youth in Mind. (2012). Information for researchers and clinicians about the Development and Well-Being Assessment. Retrieved 15 February 2024, from <http://www.dawba.info/>
